# Functional Domains of the Early Proteins and Experimental and Epidemiological Studies Suggest a Role for the Novel Human Polyomaviruses in Cancer

**DOI:** 10.3389/fmicb.2022.834368

**Published:** 2022-02-18

**Authors:** Ugo Moens, Carla Prezioso, Valeria Pietropaolo

**Affiliations:** ^1^Faculty of Health Sciences, Department of Medical Biology, University of Tromsø – The Arctic University of Norway, Tromsø, Norway; ^2^Microbiology of Chronic Neuro-Degenerative Pathologies, IRCSS San Raffaele Roma, Rome, Italy; ^3^Department of Public Health and Infectious Diseases, Sapienza University of Rome, Rome, Italy

**Keywords:** DDR, DnaJ, PP2A, p53, retinoblastoma, seroprevalence, signaling pathways

## Abstract

As their name indicates, polyomaviruses (PyVs) can induce tumors. Mouse PyV, hamster PyV and raccoon PyV have been shown to cause tumors in their natural host. During the last 30 years, 15 PyVs have been isolated from humans. From these, Merkel cell PyV is classified as a Group 2A carcinogenic pathogen (probably carcinogenic to humans), whereas BKPyV and JCPyV are class 2B (possibly carcinogenic to humans) by the International Agency for Research on Cancer. Although the other PyVs recently detected in humans (referred to here as novel HPyV; nHPyV) share many common features with PyVs, including the viral oncoproteins large tumor antigen and small tumor antigen, as their role in cancer is questioned. This review discusses whether the nHPyVs may play a role in cancer based on predicted and experimentally proven functions of their early proteins in oncogenic processes. The functional domains that mediate the oncogenic properties of early proteins of known PyVs, that can cause cancer in their natural host or animal models, have been well characterized and we examined whether these functional domains are conserved in the early proteins of the nHPyVs and presented experimental evidence that these conserved domains are functional. Furthermore, we reviewed the literature describing the detection of nHPyV in human tumors.

## Introduction

### The Polyomavirus Family: Genome Organization

Polyomaviruses (PyVs) are a family of small, non-enveloped viruses that can infect fish, birds, and mammals, including humans (Moens et al., 2017a). Characteristic for PyVs is the circular double-stranded DNA genome of approximately 5.0 kbp that encodes two major regulatory proteins, the large tumor antigen (LT-ag) and the small tumor antigen (sT-ag), and at least two structural proteins (VP1 and VP2). The regulatory genes and structural genes are separated by a non-coding control region (NCCR) encompassing the origin of replication and the promoter/enhancer sequences (DeCaprio and Garcea, 2013; Moens et al., 2017b). The regulatory proteins are expressed early during infection and participate in viral replication and viral transcription, while the structural proteins, which are expressed later in the infection cycle, form the capsid (DeCaprio and Garcea, 2013; Baez et al., 2017; Moens et al., 2017b). LT-ag contains an origin binding domain (OBD) that binds tandem repeated 5′-GRGGC-3′ motifs in the NCCR and a helicase/ATPase domain in its C-terminus. The OBD and helicase/ATPase domain are required for viral genome replication (Borowiec et al., 1990). Many PyVs encode additional regulatory and structural proteins (e.g., middle tumor antigen or MT-ag, ALTO, VP3, VP4, agnoprotein) (Carter et al., 2013; Moens et al., 2017b; Saribas et al., 2019).

To date, fifteen PyVs have been isolated from different human specimens (Moens et al., 2020). The first human polyomaviruses (HPyVs), BKPyV, and JCPyV, were identified in 1971 (Gardner et al., 1971; Padgett et al., 1971). In 2007, two new HPyVs, Karolinska Institute Polyomavirus (KIPyV) and Washington University Polyomavirus (Allander et al., 2007) (WUPyV) (Gaynor et al., 2007), were detected, and in the following years, Merkel cell Polyomavirus (MCPyV) (Feng et al., 2008), HPyV6 (Schowalter et al., 2010), HPyV7 (Schowalter et al., 2010), Trichodisplasia spinulosa polyomavirus (TSPyV) (van der Meijden et al., 2010), HPyV9 (Sauvage et al., 2011; Scuda et al., 2011), HPyV10 and its variants Malawi polyomavirus (MWPyV) and Mexico polyomavirus (MXPyV), (Buck et al., 2012; Siebrasse et al., 2012; Yu et al., 2012), Saint Louis polyomavirus (STLPyV) (Lim et al., 2013), HPyV12 (Korup et al., 2013), New Jersey Polyomavirus (NJPyV) (Mishra et al., 2014), and Lyon IARC polyomavirus (LIPyV) (Gheit et al., 2017) have been described. Recently in 2019, Ondov et al. (2019) identified the Quebec polyomavirus (QPyV) sequences in the stool from one patient through the MinHash algorithm. Not all PyVs, originally isolated from human specimens, may be true HPyVs. HPyV12, first described in a human liver sample, was later shown to infect shrews as its natural host and was therefore reclassified as a *Sorex araneus* PyV (Sara-PyV1) by the International Committee on Taxonomy of Viruses (ICTV) (Gedvilaite et al., 2017). LIPyV DNA was first amplified from human skin but is most likely a feline PyV (Fahsbender et al., 2019; Li et al., 2021). LIPyV and QPyV have not yet been listed as an HPyV by the ICTV. QPyV DNA has been detected in urine from systemic lupus erythematosus patients, multiple sclerosis patients, and pregnant women, but more studies are required to confirm whether this is a genuine HPyV (Prezioso et al., 2021).

In this review we define novel human polyomaviruses (nHPyVs) as KIPyV, WUPyV, MCPyV, HPyV6, HPyV7, TSPyV, HPyV9, HPyV10, STLPyV, HPyV12, LIPyV, and QPyV. Although HPyV12/Sara-PyV1 and LIPyV are not genuine HPyVs, and QPyV has not been classified as a HPyV, we will use the name HPyV12 and include HPyV12, LIPyV and QPyV as “nHPyVs”. Simian virus 40 (SV40) has also been detected in healthy and malignant samples from humans but is not considered a HPyV (Moens et al., 2017a). Because of its tremendous importance in understanding the oncogenic potentials of PyV, SV40 will be included as the prototype transforming PyV. Murine PyV (MPyV) and hamster Pyv (HaPyV) will also be discussed.

### Seroprevalence of Human Polyomaviruses in the Healthy Population

Serological studies mainly based on the presence of HPyV VP1 antibodies detected by a VP1- or virus-like particle-based ELISA have demonstrated that HPyV infection is very common in healthy individuals. For most HPyVs, seroprevalence in the adult healthy population is > 70%, and most individuals are infected with more than one HPyV (Kean et al., 2009; Gossai et al., 2016; Kamminga et al., 2018). Seroprevalences for HPyV12, NJPyV-2013, and LIPyV are < 10% in all age categories tested, whereas another study reported a prevalence of 97% and 58% for HPyV12 and NJPyV-2013, respectively (Gaboriaud et al., 2018; Kamminga et al., 2018). The seroprevalence of QPyV has not been examined. A significant number of individuals acquire HPyVs already during their early life, which might become a requirement for the onset of a possibly associated disease or cancer later in the lifecycle (Kean et al., 2009; Gossai et al., 2016; Kamminga et al., 2018).

### Human Polyomaviruses as Proven Causative Agents in Human Diseases

So far, six HPyVs are firmly associated with diseases. BKPyV can cause nephropathy and hemorrhagic cystitis in kidney and in hematopoietic stem cell transplants, respectively (Helle et al., 2017); JCPyV is a causative agent of progressive multifocal leukoencephalopathy (PML), primarily in HIV-positive patients (Cortese et al., 2021); TSPyV is linked to the rare skin disease trichodysplasia spinulosa (TS) (Kazem et al., 2013), and HPyV6 and HPyV7 are associated with pruritic rash (Klufah et al., 2021).

A human virus is considered a tumor virus when viral sequences or proteins are regularly and persistently present in tumors and there is epidemiological evidence that virus infection represents a major risk factor for cancer development. Moreover, it is demonstrated that the virus or specific virus genes can transform cells or induce tumors in suitable animal models and that transformation of cell and tumor induction in animals depends on continuous expression of viral protein(s) (zur Hausen, 2001). According to these criteria, IARC has classified the human viruses Epstein-Barr virus, Kaposi’s sarcoma-associated herpes virus, hepatitis B virus, hepatitis C virus, high-risk human papillomaviruses, human T-cell lymphotropic virus type 1, and human immunodeficiency virus as group 1 carcinogenic viruses (i.e., carcinogenic to humans) (Bouvard et al., 2009). As for HPyV, only 3 members may be associated with cancer. Presently, MCPyV is the only HPyV considered to cause cancer in its host. Approximately 80% of Merkel cell carcinomas (MCC) are positive for the MCPyV genome, which is typically integrated and encodes a truncated form of LT-ag (Chang and Moore, 2012). As early as in 2012, MCPyV has been categorized as a group 2A carcinogen by the International Agency for Research on Cancer (IARC) (Bouvard et al., 2012). MCPyV has also been discovered in non-neoplastic B cells and neoplastic B cells, thus suggesting a role in B-cell neoplasia (Pantulu et al., 2010; Teman et al., 2011; Imajoh et al., 2012). BKPyV and JCPyV have been suspected to be involved in renal, prostate, colon and brain cancer (Fioriti et al., 2005; Niv et al., 2005; White et al., 2005; Delbue et al., 2014, 2017; Keller et al., 2015; Starrett and Buck, 2019; Ahye et al., 2020). Both viruses are classified as possibly carcinogenic to humans by the International Agency for Research on Cancer (Bouvard et al., 2012).

The aim of this review is to provide an overview of the pro and contra evidence that argues for or against a possible role of nHPyVs in cancer. The implication of MCPyV in cancer has been extensively elaborated on in recent reviews (Chang and Moore, 2012; Becker et al., 2017; Csoboz et al., 2020; Pietropaolo et al., 2020; DeCaprio, 2021; Krump and You, 2021), so this virus will therefore only briefly be considered in this review. Although an emerging role for HPyV6 and HPyV7 in cancer was recently described in an excellent review (Klufah et al., 2021), we will include these two viruses.

## Conserved Domains in the Early Proteins of Novel Human Polyomaviruses That May Contribute to Oncogenesis

### Transformation Functions of the Early Proteins of SV40, MPyV, and MCPyV

The story of PyVs begins in the 1950s when Ludwik Gross showed that a filterable agent from leukemia extract from the inbred Ak mice, a strain that spontaneously developed leukemia, could cause tumors of the parotid when injected in newborn non-leukemic C3H mice. Hence, he named the virus parotid tumor virus. However, some mice developed additional tumors, and this was confirmed by work by Sarah Stewart and Bernice Eddy, who then renamed the virus SE polyomavirus reviewed in Morgan (2014). Our understanding of how PyV causes tumors came with the isolation of another polyomavirus from rhesus macaque origin, SV40. This virus was discovered in 1960 as a contaminant of poliovirus vaccines (Sweet and Hilleman, 1960; Hilleman, 1998). SV40 can transform cells, including human cells, induce tumors in animal models (but not its natural host), and can be detected in human tumors. As previously mentioned, a role of MCPyV in human cancer was demonstrated in 2008 (Feng et al., 2008).

The major oncoprotein of SV40 is LT which exerts is transforming functions by interfering with the tumor suppressors retinoblastoma protein and p53. SV40 LT has also been shown to bind the mitotic spindle checkpoint kinase Bub1, the E3 ubiquitin kinase Cul7, the insulin receptor substrate 1, and the DNA repair enzyme Nbs1. These interactions contribute to the oncogenic properties of SV40 LT [reviewed in Cheng et al. (2009)]. SV40 sT alone cannot transform cells but cooperates with LT. sT exerts its transforming role mostly by interacting with protein phosphatase 2A (PP2A) [reviewed in Cheng et al. (2009)]. The major transforming ability of MPyV depends on its middle T-antigen (MT) and sT. Both can impede the function of PP2A, whereas MT can bind and activate the tyrosine kinase Src (reviewed in Cheng et al. (2009)]. MCPyV-positive Merkel cell carcinomas express a truncated LT that can interact with retinoblastoma proteins, but not p53. *In vitro* studies have suggested that sT may be the major oncogenic protein (Chang and Moore, 2012; Pietropaolo et al., 2020; Ahmed et al., 2021). The functional motifs involved in transformation by LT, sT and MT will be described in more detail and their presence in the corresponding proteins of the nHPyVs will be discussed in the next sections.

### Functional Domains in the LT of Novel Human Polyomaviruses That May Be Involved in Oncogenic Processes

Cell culture studies with temperature sensitive mutants demonstrated that the oncogenic potential of SV40 primarily depends on its LT-ag and this was later confirmed by animal studies (Noonan and Butel, 1978; Sáenz Robles and Pipas, 2009; Hudson and Colvin, 2016). LT-ag of BKPyV and JCPyV are also strongly oncogenic in heterologous animal models (Small et al., 1986a,b; Dalrymple and Beemon, 1990; Noguchi et al., 2013; Del Valle and Khalili, 2021). SV40 LT-ag interferes with DNA repair, apoptosis, cellular transcription, protein degradation, telomerase activity, immune and inflammatory responses, and stimulate cell proliferation, angiogenesis, and cell migration. LT-ag of other PyVs such as mouse PyV (MPyV), BKPyV, and JCPyV have been shown to (at least partially) possess the same functions. The oncogenic potential of SV40 and other PyVs LT-ag predominantly depends on its ability to bind and impede the function of the tumor suppressor proteins p53 and retinoblastoma (Moens et al., 2007; Cheng et al., 2009; Topalis et al., 2013; Baez et al., 2017).

The retinoblastoma tumor suppressor family contains the proteins, pRb, p107, and p130, encoded by the *RB1*, *RBL1*, and *RBL2* genes, respectively. The retinoblastoma proteins (RB) are key proteins in regulating G1 to the S phase cell cycle progression through their interaction with the E2F transcription factors family (Genovese et al., 2006; Dick and Rubin, 2013). The interference with RB’s function by LT-ag requires a direct interaction mediated by the RB-binding motif (or pRb pocket) LXCXE (Stubdal et al., 1997; Sullivan et al., 2000; Brown and Gallie, 2002). The psycho (PTYGTX_9_F) motif is also important for RB binding. Moreover, an intact DnaJ domain, located in the N-terminal end of LT-ag, is also involved. The DnaJ domain contains the constant region 1 (CR1; LXXLL) and the Hsc70 binding motif HPDKGG^D^/_N_ (Campbell et al., 1997; Srinivasan et al., 1997; Sullivan and Pipas, 2002). The binding of LT-ag to RB promotes the activation of E2F, resulting in expression of genes required for S phase progression. Hsc70 is a chaperone with weak ATPase activity that binds to the DnaJ motif HPDKNG^N^/_D_. The binding of Hsc70 to LT-ag increases the intrinsic ATPase activity of Hsc70, with this interaction helping to disrupt pRb proteins/E2F complexes (Sullivan et al., 2000; Garimella et al., 2006; Salma et al., 2007). The binding of SV40 LT-ag and JCPyV LT-ag to Hsc70 stimulates cell cycle progression, and influences viral DNA replication, transformation, viral and cellular promoter activity, as well as virion assembly [reviewed in Frisque et al. (2006), Sullivan and Pipas (2002)]. LT-ags of SV40, BKPyV, and JCPyV bind all three retinoblastoma proteins, and may explain LT-ag’s transforming properties *in vitro* and *in vivo* (Harris et al., 1996; White and Khalili, 2006). The CR1, the Hsc70 binding motif, and the RB-binding motif seems to be conserved in the LT-ag of most nHPyVs. A possible interaction between RB and LT-ag was not examined for all nHPyVs. LT-ag of MWPyV was found to bind pRb, p107 and p130, but failed to increase expression of the E2F target genes *CCNA* (encoding cyclin A), *CCNE* (encoding cyclin E), and *MYBL2* (encoding B-MYB), and to decrease pRb levels contrary to SV40 LT-ag (Berrios et al., 2015). LT-ags of WUPyV, HPyV6, HPyV7, and TSPyV were also found to interact with the family member pRb by co-immunoprecipitation assays with lysates of cells overexpressing LT-ag (Rozenblatt-Rosen et al., 2012; Wu et al., 2016a; Nako et al., 2020). The biological relevance of HPyV7 LT-ag and pRb interaction remains unknown as HPyV-7 LT-ag expression in thymic epithelial tumors did not correlate with the phosphorylation of pRb (Keijzers et al., 2015). TSPyV LT-ag clusters with the cell proliferation marker Ki-67 and with phosphorylated pRb in hair follicles of TS-affected patients, thus suggesting a role for TSPyV LT-ag in cell proliferation and a potential driver of papule and spicule formation, typical for trichodysplasia spinulosa (Kazem et al., 2014).

**FIGURE 1:**
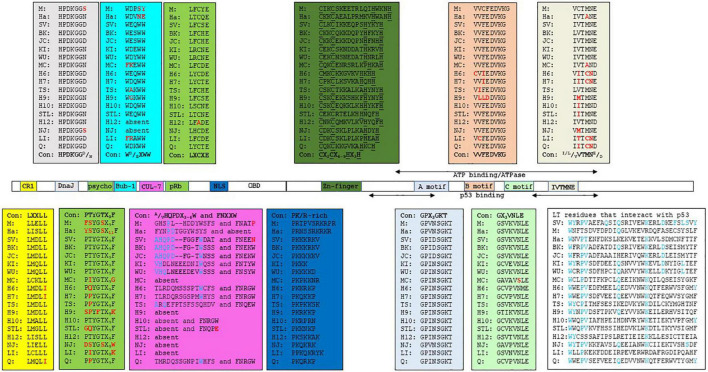
Functional domains in the LT-ags of HPyVs. The amino acid sequence of the functional domains is given in the corresponding colored box. The consensus sequence (Con) for each domain is shown. M = MPyV (J02288), Ha = HaPyV (NC_001663), SV = SV40 (NC_001669), BK = BKPyV (NC_001538), JC = JCPyV (NC_001699), KI = KIPyV (NC_009238), WU = WUPyV (NC_009539), MC = MCPyV (NC_010277), H6 = HPyV6 (NC_014406), H7 = HPyV7 (NC_014407), TS = TSPyV (NC_014361), H9 = HPyV9 (NC_015150), H10 = HPyV10 (JX262162), STL = STLPyV (NC_020890), H12 = HPyV12 (NC_024118), NJ = NJPyV-2013 (NC_024118), LI = LIPyV (NC_034253), and Q = QPyV (BK010702. The accession number is given in parenthesis. CR1 = conserved region 1.

Another essential LT-ag interaction in PyVs-mediated tumorigenesis is that with p53, a tumor-suppressing protein that regulates the gene expression in response to stressful events, such as DNA damage, leading to cell apoptosis, cell cycle arrest, or senescence. p53’s function is usually deregulated in many cancer types (Muller and Vousden, 2013). The interaction of PyV LT-ag with p53 requires the C-terminal part of the protein, which also contains the helicase/ATPase domain. The interaction of LT-ag with p53 prevents p53 from binding to DNA, and represses the transactivation domain of p53 (Sheppard et al., 1999). During SV40 carcinogenesis, LT-ag binds and blocks p53 activity, thereby preventing apoptosis, cell cycle arrest, DNA repair and angiogenesis (Vogelstein et al., 2000; Levine and Oren, 2009). Kellogg *and coworkers* determined the percentage identity across the p53 domains of HPyVs BK, JC, KI, WU, MC, 6, 7, TS, 10, STL, and NJ with the SV40 LT-ag p53-interaction domain (Kellogg et al., 2021). BKPyV and JCPyV LT-ag, which have been shown to interact with p53, had the highest identity (67 and 69%, respectively). They also predicted that the interaction of SV40 LT-ag with p53 requires 13 residues: D402, W581, Y582, P584, V585, Q590, Q593, K600, D604, F607, L609, S610, and Y612. Only W581 and Q583 are conserved in MPyV LT-ag. Accordingly, MPyV LT-ag does not bind p53 (Qian and Wiman, 2000). There is a high conservation among these residues, with many identical or similar in LT-ag of HPyVs and MPyV and HaPyV (Figure 1 and Supplementary Figure 1). Their computational docking studies of p53 with the LT-ags of BKPyV, JCPyV, KIPyV, WUPyV, MCPyV, HPyV6, HPyV7, TSPyV, MWPyV, STLPyV, and NJPyV supported the possibility of all LT-ags to bind p53. These findings predict the possibility of nHPyV LT-ags to interact with p53 in a manner similar to SV40 LT-ag. A direct interaction between the LT-ag of BKPyV and the LT-ag of JCPyV and p53 has been demonstrated (Shivakumar and Das, 1996; Staib et al., 1996). Less is known about the nHPyVs. Full-length MCPyV LT-ag fails to bind p53, whereas the truncated LT-ag form expressed in MCC cells lacks the C-terminal domain, and hence the p53-binding region (Cheng et al., 2013; Borchert et al., 2014). The TSPyV LT-ag expressed in HEK293 cells did not or only weakly bound to p53 (Wu et al., 2016a; Nako et al., 2020). The reciprocal co-immunoprecipitation with lysates of osteosarcoma U2OS cells ectopically expressing MWPyV LT-ag, an HPyV10 variant with > 95% nucleotide identity (Siebrasse et al., 2012), and p53 demonstrated an interaction between these two proteins (Berrios et al., 2015). However, compared to SV40 LT-ag, MWPyV LT-ag was less stable and could not stabilize p53, nor could MWPyV LT-ag promote the growth of human diploid fibroblast IMR-90 cells (Berrios et al., 2015). The rapid turn-over of MWPyV LT-ag compared to SV40 LT-ag may explain its inability to promote cell growth and its lack of oncogenic potential.

SV40 LT-ag can interact with additional cellular proteins, which may contribute to viral transformation (Cheng et al., 2009). One of these SV40 LT-ag-binding proteins is the mitotic checkpoint serine-threonine protein kinase Bub1 (Cotsiki et al., 2004; Hein et al., 2009). Impaired function of Bub1 leads to chromosomal instability, as observed in cells expressing SV40 LT-ag (Hu et al., 2013). Interaction with Bub1 requires SV40 LT-ag residues 88-98, which contain the WEQWW motif. The LT-ags of most nHPyVs contain the conserved motif W^D^/_E_XWW (Figure 1 and Supplementary Figure 1). The oncogenic MCPyV, MPyV, and HaPyV lack the Bub1-binding motif (Figure 1), and LT-ag of the HPyV6 isolate H6-cg-A2f.11 has the mutated WGQWW motif, suggesting that the Bub1:LT-ag interaction may not be absolutely required for HPyVs’ transformation *in vivo* (Torres et al., 2018).

Another mechanism by which SV40 LT-ag can induce transformation is through interaction with the cellular protein Cul7, an E3 ubiquitin ligase (Kohrman and Imperiale, 1992; Ali et al., 2004). Binding requires residues 69-81 (AHQPDFGGFWDAT) and 98-102 (FNEEN). Mutation of F98 diminishes Cul7 binding, while deletion of amino acids 68-83 abolishes it (Cavender and Tevethia, 2016). Cul7 binding to SV40 LT-ag was shown to play a role in transformation because mouse embryonal fibroblasts (MEFs) expressing non-Cul7 binding LT-ag mutants were unable to form colonies in soft agar, while wild-type expression cells were able to do so (Ali et al., 2004). The FNEEN motif is partially conserved in the nHPyV KI, WU, 6, 7, 10, TS, STL, and Q, whereas only the BKPyV, JCPyV, KIPyV, and WUPyV LT-ags show reminiscent identity with the SV40 LT-ag 69-81 amino acid sequence (Figure 1 and Supplementary Figure 1). The interaction between LT-ags from the nHPyV and Cul7 has not been studied, although the low sequence identity may indicate that no such binding occurs.

Insulin receptor substrate 1 (IRS1) is a component of the insulin-like growth factor (IGF-I) signaling pathway that transduces signals from the IGF-I receptor (IGF-IR). SV40 LT-ag was found to bind IRS1 (Fei et al., 1995). The biological importance of the IRS-LT-ag interaction in transformation is underscored by the observation that SV40 LT-ag is unable to transform IGF-IR^–/–^ MEFs, whereas LT-ag lacking the N-terminal 250 amino acids fails to bind IRS1 and to transform IGF-IR^–/–^ MEFs overexpressing IRS1 (Sell et al., 1993; Fei et al., 1995). The E107K mutation in the pRb motif LFCYE abrogated binding of SV40 LT-ag to IRS. Despite conservation of this residue, it is not known whether LT-ag of the other HPyVs can bind IRS1, with the exception of JCPyV LT-ag. JCPyV LT-ag was found to bind IRS1, resulting in nuclear translocation of IRS-1. IRS-1 could then bind Rad51 and inhibit homologous recombination DNA repair (Lassak et al., 2002; Reiss et al., 2006).

The interaction of SV40 LT-ag with the DNA repair enzyme Nbs1 disrupts DNA replication control and has been suggested to help immortalization of cells. This interaction is mediated by the DNA binding domain of SV40 LT-ag (Lanson et al., 2000; Wu et al., 2004). It is not known whether LT-ags of other HPyVs can associate with Nbs1.

### Functional Domains in the sT of Novel Human Polyomaviruses That May Be Involved in Oncogenic Processes

**FIGURE 2:**
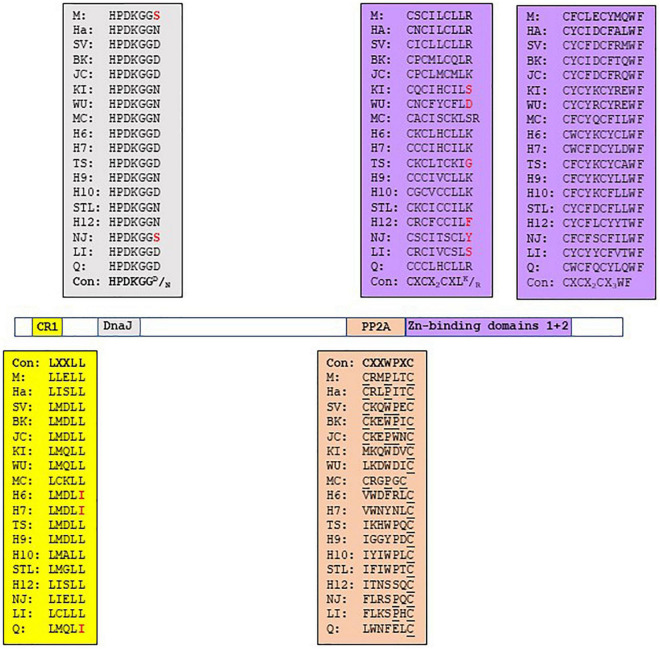
Functional domains in the sT-ags of HPyVs. The amino acid sequence of the functional domains is given in the corresponding colored box. See legend Figure 1 for details.

The HPyV early region encodes another regulatory protein, sT-ag, which is translated from an alternative spliced early transcript. Although sT-ag expression is not a constantly condition for viral replication, it is required for optimal PyV replication (Kwun et al., 2009; Tsang et al., 2016). PyV sT-ag has oncogenic properties in both cell culture and animal models. SV40 sT-ag can transform cells, including human cells, and can alone or in combination with LT-ag induce tumors in transgenic animals (Choi et al., 1988; Ratineau et al., 2000; Goetz et al., 2001; Yu et al., 2001; Ahuja et al., 2005). MCPyV sT transgenic mice will also develop tumors (Shuda et al., 2015; Verhaegen et al., 2015, 2017). Moreover, SV40 sT-ag can also influence the expression of cellular genes, including proto-oncogenes and tumor suppressor genes (Moens et al., 1997). The N-terminal regions of LT-ag and sT-ag share approximately 80 amino acids, which includes the CR1 with the motif LXLL and the Hsc70 binding HPDKNG^N^/_D_ sequence (Figure 2 and Supplementary Figure 2). SV40 sT-ag can interact with Hsc70, but studies with SV40 LT-ag mutants have shown that additional sequences C-terminal to the DnaJ and LXCXE motifs (the latter is not present in sT-ag) are required for stable interaction with Hsc70 (Sullivan et al., 2001; Genevaux et al., 2003). The importance of sT:Hsc70 interaction in possible nHPyV-induced cancers is unknown.

BKPyV and JCPyV sT-ags, but none of the other HPyVs, nor SV40, MPyV and HaPyV, contain a putative pRb motif LXCXE. JCPyV sT-ag contains the LYCKE and LHCWE motifs, whereas BKPyV sT-ag has only one LYCKE motif (Supplementary Figure 2). These LXCXE motifs are therefore potentially able to interact with pRb family proteins. Indeed, JCPyV sT-ag was found to interact with the pRb family members p107 and p130. The C157A mutation in the LHCWE motif did not abrogate the interaction with p107 and p130 (Bollag et al., 2010), which may indicate that the LYCKE motif rather than the LHCWE sequence is required for binding RB. The authors did not test whether BKPyV sT-ag, which contains the LYCKE motif, binds p107/p130, but showed that the C157A substitution reduced JCPyV replicated by 20-30% compared to wild-type virus. The importance of the two LXCXE motifs in JCPyV sT-ag is underscored by the finding that mutations in these sites are rare. In fact, only two isolates have been described in which the LHCWE motif was changed into LHRWE and FHCWE, respectively (accession numbers AAL37643 and AAK 70296). Whether mutations in the LXCXE motifs affect the oncogenic properties of JCPyV sT-ag remains to be examined.

The major oncogenic potential of sT-ag is attributed to its ability to bind the PP2A, since mutants that fail to bind PP2A cannot induce tumorigenic activity (Hahn et al., 2002). PP2A is a complex that consists of a structural A, a regulatory B, and a catalytic C subunit. Several isoforms of each subunit exist, with the composition of PP2A holoenzyme determining its substrate specificity and catalytic activity (Janssens et al., 2005; Wlodarchak and Xing, 2016). This enzyme plays a multi-faceted role in the regulation of the cell cycle and apoptosis by dephosphorylating protein targets such as AKT, p53, c-MYC, and β-catenin. PP2A is identified as a bona fide tumor suppressor (Janssens et al., 2005; Seshacharyulu et al., 2013; Wlodarchak and Xing, 2016). The sT-ags of SV40, BKPyV, JCPyV, and MCPyV, in addition to MT of murine PyV, bind and inactivate PP2A. This interaction promotes cell transformation, except for MCPyV sT-ag because mutations that abolished PP2A interaction had no effect on sT’s activity to transform rat fibroblasts or induce tumors in mice (Arroyo and Hahn, 2005; Sablina and Hahn, 2008; Cheng et al., 2009, 2017; Shuda et al., 2011; Griffiths et al., 2013; Kwun et al., 2015; Abdul-Sada et al., 2017; Verhaegen et al., 2017). However, the sT-ags of these PyVs seem to bind different subunits of PP2A and with different affinities. SV40 sT-ag interacts with B55α, B56α, and B56ϵ; BKPyV sT with Aα; JCPyV sT-ag with Aα, C subunit, and the AC core; and MCPyV sT-ag with Aα, Aβ, B5α, Cα, and Cα. The sT-ags of HPyV6 and TSPyV bind the A and C subunits of PP2A [reviewed in Moens and Macdonald (2019)]. The interaction of SV40 sT-ag with PP2A requires the DnaJ domain and the two Zn-binding domains (Figure 2 and Supplementary Figure 2; Mateer et al., 1998; Cho et al., 2007). Nonetheless, the DnaJ domain of SV40 sT-ag contributes to a high affinity binding to the A subunit of PP2A but is not absolutely essential for the sT:PP2A interaction (Mateer et al., 1998). The substitutions R7A and R21A in the DnaJ domain of SV40 sT-ag disrupted the interaction with the A subunit of PP2A. The second Zn-binding domain (see below) seems to be the primary docking site for PP2A, while the DnaJ domain may stabilize the binding (Cho et al., 2007). A CXXWPXC consensus sequence is also present in the sT-ags of BKPyV, JCPyV, whereas HaPyV and MPyV sT-ags have a CXXPXXC motif, and MCPyV sT-ag has the CXXPXC motif. The variation in the CXXWPXC consensus sequence may help explain the difference in the affinity and specificity of sT-ag for the PP2A subunits. The corresponding sequence of the other HPyVs shows little similarity with the consensus CXXWPXC motif, suggesting that they may not or poorly interact with PP2A. Despite a poor similarity, TSPyV sT-ag and MT-ag, and the sT-ags of HPyV6, HPyV7, and MWPyV were shown to bind PP2A (Nguyen et al., 2014; Berrios et al., 2015; Wu et al., 2017a,b, 2019b) and to stimulate signaling pathways in a PP2A-dependent manner (see section 3.2). It is not known whether the sT-ag of the other nHPyVs binds PP2A. Other amino acids outside the CXXWPXC motif in sT-ag have also been found to be crucial for PP2A binding. SV40 sT-ag also required the CXCX_2_CXLR motif, which is part of the Zn-biding domain [see next paragraph; (Mateer et al., 1998)]. This CXCX_2_CXL^R^/_K_ is conserved in the sT-ags of MPyV, HaPyV, and HPyVs except for KIPyV, WUPyV, HPyV12, NJPyV-2013, and LIPyV, which do not have the terminal basic residue, and MCPyV sT-ag, which has an additional residue before the basic residue (Figure 2 and Supplementary Figure 2). Single substitutions of residues R7A, K118A, I122A, L126A, H130A, K134A, L142A, E146A, and Y150A in MCPyV sT-ag all disrupted the interaction with PP2A (Kwun et al., 2015). Only residues I122 and L126 are part of the CXCX_2_CXL^R^/_K_ motif, indicating that additional residues are involved in the MCPyV sT:PP2A interaction. The first Zn-binding domain with the CX_5–7_CXCX_2–3_CX_4–5_H motif in the C-terminus of sT-ag is conserved in all HPyVs, except in HPyV10 sT-ag and NJPyV sT-ag, which have CX_8_CXCX_2_CX_5_H and CX_7_CXCX_3_CX_4_H, respectively (Figure 2 and Supplementary Figure 2). The second Zn-binding domain CXCX_2_C is also present in the sT-ags of MPyV, HaPyV, SV40 and HPyVs. Both Zn-binding domains contribute to the stability of sT-ag (Jog et al., 1990; Turk et al., 1993). For SV40 sT-ag, it was shown that the second Zn-binding motif participates in the interaction with the A subunit of murine PP2A, whereas the first Zn-binding motif may be involved in an interaction with the C subunit of PP2A (Cho et al., 2007). The second Zn-binding domain has a conserved WF at position + 3/ + 4 (Figure 2). Mutation of W into A abrogated the interaction of SV40 sT-ag with the PP2A Aa subunit (Cho et al., 2007). Although not demonstrated for all nHPyVs, conservation of the PP2A binding motifs in the sT-ags of the nHPyVs suggests that they can also interact with PP2A.

Expression of TSPyV sT-ag was shown to induce hyperphosphorylation of pRb, an event associated with S-phase induction and increased cell proliferation (Wu et al., 2016b). It remains unknown as to whether TSPyV sT-triggered phosphorylation of pRb depends on sT’s ability to inactivate PP2A, nor were the biological consequences and the possible binding of TSPyV sT-ag to pRb investigated. The direct interaction between TSPyV sT-ag and pRb seems unlikely because sT-ag does not contain the RB-binding motif.

### Functional Domains in Additional Early Proteins of Novel Human Polyomaviruses That May Be Involved in Oncogenic Processes

The murine polyomavirus (MPyV) and hamster polyomavirus (HaPyV) can transform a wide variety of cells and can induce tumors in their natural host (Benjamin, 2001; Jandrig et al., 2021). In addition to LT-ag and sT-ag, MT-ag also contributes to neoplastic transformation and can cause tumors in transgenic animals (Templeton and Eckhart, 1982; Bautch et al., 1987; Freund et al., 1992; Cheng et al., 2009; Fluck and Schaffhausen, 2009). These three viral oncoproteins are produced by differential splicing of the viral early region (Fluck and Schaffhausen, 2009). The pattern of splicing is such that all share a common N-terminal 79 amino-acid, whereas MT-ag and sT-ag share an additional 112 amino acids. LT-ag, MT-ag, and sT-ag each have a unique C-terminal sequence of 706, 230, and 4 amino acids, respectively. The common region with sT-ag encompasses the PP2A binding site, whereas the unique MT region can bind the c-SRC tyrosine kinase and has a hydrophobic transmembrane domain in its C-terminal end (Cheng et al., 2009; Fluck and Schaffhausen, 2009).

**FIGURE 3:**
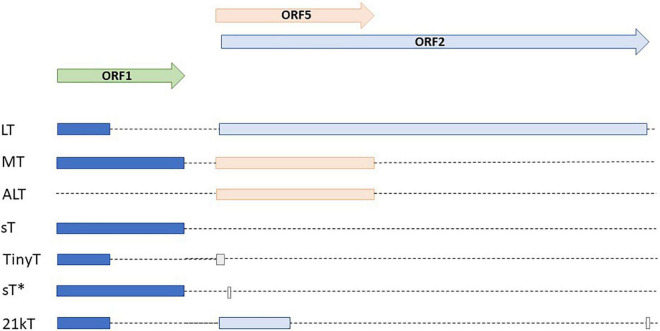
Alternative early transcripts of TSPyV. Modified after (van der Meijden et al., 2015; van der Meijden and Feltkamp, 2018).

**FIGURE 4:**
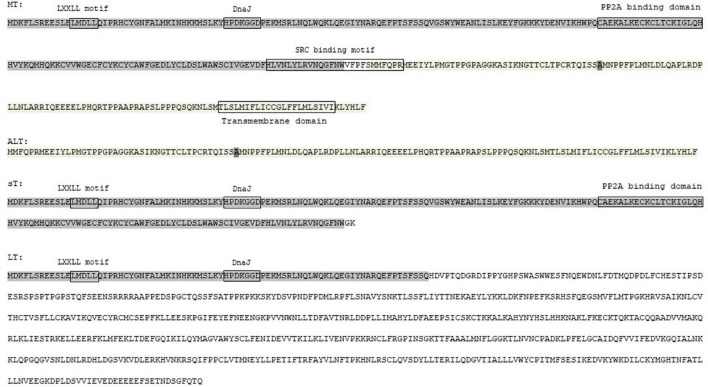
Amino acid sequence of MT-ag, ALT, sT-ag and LT-ag of TSPyV. The common sequences are enlightened in the same color. Putative functional motifs are shown.

Of the nHPyVs, expression of alternative proteins was only investigated for MCPyV and TSPyV. MCPyV encodes LT-ag, sT-ag, 57kT, and an alternative T-antigen (ALTO) (Shuda et al., 2008; Carter et al., 2013). The function of the latter two is incomplete understood. Immortalized human foreskin fibroblasts BJ-hTERT expressing 57kT grew more slowly than control cells, thereby suggesting that this protein has a growth inhibitory function (Cheng et al., 2013). ALTO does not seem to be required for viral replication (Shuda et al., 2008). Expression of the TSPyV early region in HeLa and HEK293T cells resulted in six differentially spliced transcripts with the potential to encode LT-ag, MT-ag, sT-ag, tiny T, 21kT, and alternative T (ALTO) (Figure 3; van der Meijden et al., 2015). For all of them, except tiny T, RNA was detected in the skin from TSPyV-infected patients. LT-ag, MT-ag, and ALTO protein expression was confirmed in HEK293T cells transfected with the TSPyV early region. The TSPyV MT-ag is 332 aa long and contains putative PP2A and SRC binding domains, and a hydrophobic transmembrane domain in the C-terminus (Figure 4 and Supplementary Figure 3). The ALTO protein of MCPyV also contains a hydrophobic rich C terminus and deletion of this domain changed the subcellular localization of ALTO from cytoplasmic foci to a diffuse distribution in the cytoplasm (Carter et al., 2013). The TSPyV ALTO is also detected in the cytoplasm, although the effect of deleting the hydrophobic C-terminal domain was not investigated (van der Meijden et al., 2015).

No alternative early proteins for the other nHPyVs have been predicted or detected, with the exception of STPyV and NJPyV-2013. STLPyV encodes a hypothetical MT, which contains the putative PP2A domain and a hydrophobic rich C-terminus. However, there is little similarity with the putative SRC domain of TSPyV MT (Supplementary Figure 3). For NJPyV-2013 a transcript encoding a putative 299 aa alternative T-antigen could be amplified from muscle tissue from a NJPyV-2013 positive patient (Mishra et al., 2014). The existence and possible function of these plausible STLPyV and NJPyV-2013 proteins remain to be investigated.

## Epidemiological Evidence for a Role of the Novel Human Polyomaviruses in Cancer

### Detection of Novel Human Polyomaviruses in Tumor Samples

Previous studies had shown a higher prevalence of BKPyV in urothelial and renal cancers and of JCPyV in brain tumors and colorectal cancers, compared to healthy tissue [(Delbue et al., 2014; Keller et al., 2015; Starrett and Buck, 2019; Shavaleh et al., 2020; Shoraka et al., 2020); see Supplementary Table 1]. MCPyV is associated with approximately 80% of all MCC cases (Arora et al., 2012; Becker et al., 2017; Pietropaolo et al., 2020). The presence of nHPyVs has been examined in several human tumor tissues (Supplementary Table 1). Yet, the lack or very infrequent detection of (RT-)PCR amplified nHPyV sequences, nHPyV proteins by immunohistochemistry, and nHPyV reads after deep sequencing does not support a role of nHPyVs in human cancers (Table 1 and Supplementary Table 1). Exceptions are HPyV 6 and HPyV7 for which an emerging role has been suggested in certain skin cancers and non-cutaneous malignancies, respectively [(Klufah et al., 2021; Supplementary Table 1].

**TABLE 1:** Prevalence of novel HPyV in human tumor samples.

	Oral Cavity	Respiratory system	Digestive system	Excretory system	Reproductive system	Integumentary System	Nervous system	Circulatory system	Endocrine system	skeleton
KI	0/475 (0)*	10/677 (1.5)	0/148 (0)	0/399 (0)	0/434 (0)	4/258 (1.6)	0/767 (0)	0/194 (0)	0/122 (0)	ND#
WU	1/447 (0.2)	0/725 (0)	0/270 (0)	0/399 (0)	0/434 (0)	0/258 (0)	0/767 (0)	0/72 (0)	0/122 (0)	ND
H6	15/542 (2.8)	0/370 (0)	24/380 (6.3)	0/211 (0)	1/334 (0.3)	164/2212 (7.4)	0/638 (0)	0/72 (0)	0/53 (0)	0/33 (0)
H7	4/559 (0.7)	0/441 (0)	26/300 (8.7)	0/211 (0)	1/334 (0.3)	41/1347 (3)	1/646 (0.2)	0/72 (0)	0/53 (0)	0/33 (0)
TS	0/447 (0)	1/457 (0.2)	0/148 (0)	0/211 (0)	1/426 (0.2)	5/890 (0.6)	0/559 (0)	0/73 (0)	0/47 (0)	ND
H9	0/475 (0)	0/413 (0)	0/260 (0)	0/399 (0)	0/280 (0)	2/618 (0.3)	0/629 (0)	1/100 (1)	ND	ND
H10	8/363 (2.2)	0/569 (0)	0/141 (0)	0/211 (0)	0/157 (0)	13/271 (4.8)	0/638 (0)	0/34 (0)	ND	ND
STL	0/269 (0)	0/473 (0)	0/130 (0)	0/202 (0)	0/143 (0)	1/246 (0.4)	0/638 (0)	0/34 (0)	ND	ND
H12	0/269 (0)	0/326 (0)	0/130 (0)	0/202 (0)	0/143 (0)	0/317 (0)	0/590 (0)	0/72 (0)	ND	ND
NJ	0/269 (0)	0/326 (0)	0/130 (0)	0/202 (0)	0/143 (0)	1/82 (1.2)	0/590 (0)	0/72 (0)	ND	ND
LI	ND	0/2 (0)	ND	ND	0/58 (0)	0/17 (0)	0/329 (0)	0/34 (0)	ND	ND
Q	ND	0/2 (0)	ND	ND	0/58 (0)	0/3 (0)	0/329 (0)	0/34 (0)	ND	ND

See text and Supplementary Table 1 for details and references. *Number of samples positive of total number of samples tested (%).

*#Not done.*

### Correlation Between Novel Human Polyomaviruses Antibodies and Cancer

Serological studies have proven that most healthy individuals are infected with one or several HPyVs, and possess antibodies against the major capsid protein VP1 (see section 1.2). Thus, comparing VP1 seroprevalence between healthy individuals and cancer patients may be inconclusive. The prevalence of antibodies against LT-ag or sT-ag may be a better marker as was shown for patients with MCPyV-positive MCC compared to non-MCC individuals. While only 2% (respectively, 1%) of 530 non-malignant subjects had antibodies against LT-ag (respectively, sT-ag), 26% (respectively, 41%) of 205 MCPyV-positive MCC patients were seropositive for LT-ag (respectively, sT-ag) (Paulson et al., 2010). This significant difference in anti-MCPyV oncoprotein antibodies was confirmed in another study, showing seropositivity in 1% of healthy persons (n = 100) against 52% in 219 MCC patients (Paulson et al., 2017). Only a few studies have examined the seroreactivity against nHPyVs in normal and cancer patients. Even so, most of these studies have monitored antibodies against VP1 and did not investigate the presence of the virus in the tumor. Comparing VP1 antibody prevalence in women with invasive cervical cancer or cervical intraepithelial neoplasia grade 3 with matched controls did not show significant differences (Castellsagué et al., 2014). Teras et al. (2015) determined the presence of antibodies against KIPyV, WUPyV, HPyV6, HPyV7, and TSPyV VP1 as a prognostic risk factor for non-Hodgkin lymphoma. They found that there was no association of these HPyVs with the risk of non-Hodgkin lymphoma risk, although a not statistically significant inverse trend for TSyV antibodies and non-Hodgkin lymphoma risk was observed. Similarly, no clear association between KIPyV, WUPyV, HPyV6, HPyV7, TSPyV, and HPyV10 VP1 seropositivity and squamous cell carcinoma (SCC) risk was found (Gossai et al., 2016). The authors also compared HPyV6, HPyV7, TSPyV, and HPyV10 LT-ag seropositivity in SCC patients and healthy controls. However, because of the cross-reactivity and the small number of samples that were LT-ag seropositive, the results were excluded (Gossai et al., 2016). The presence of viral nucleotide sequences or proteins in the SCC tumors was not investigated. Results from HPyV6, HPyV7, and TSPyV VP1 seroprevalence in immunocompetent patients with keratinocyte carcinomas (including cutaneous SCC and basal cell carcinoma) revealed that viral infection did not predict subsequent development of keratinocyte carcinoma, suggesting that these viruses do not play a major role in this cancer (Amorrortu et al., 2021). These finding were confirmed in a cohort of Australian cutaneous SCC patients. Comparing KIPyV, WUPyV, HPyV6, HPyV7, TSPyV, HPyV9, and HPyV10 VP1 seroprevalence in 226 individuals that developed cutaneous SCC with 462 controls showed no significant differences. The authors also tested for antibodies against HPyV7, TSPyV, and HPyV10 LT-ag and did not find statistically significant differences between the cancer patients and healthy controls. The presence of viral DNA in tumor tissue was not investigated (Antonsson et al., 2018). Likewise, no statistically significant difference in KIPyV, WUPyV, HPyV6, HPyV7, TSPyV, and HPyV10 VP1 and LT-ag seroprevalences were found between lung cancer patients and healthy controls (Malhotra et al., 2016). Another study failed to detect TSPyV LT-ag antibodies in sera from 370 healthy control and in sera from 357 chronic lymphocytic leukemia patients (Robles et al., 2015). Antibodies against KIPyV, WUPyV, HPyV6, HPyV7, and TSPyV VP1 were also examined in this study. High VP1 seroprevalence was found (77-99%) for all viruses in the healthy population, but a statistically significant decrease in seroprevalence for all viruses was detected in the patient group (Robles et al., 2015). The presence of viral DNA in tumor cells was not examined. Song et al. (2020) showed that KIPyV, WUPyV, HPyV6, and TSPyV, VP1 seropositivity was inversely associated with AIDS-related non-Hodgkin lymphoma, whereas seropositivity for HPyV7, HPyV9, and HPyV10 VP1 were non-significantly higher. Work by Bassig et al. (2018) with three different cohorts from China confirmed that TSPyV VP1 seropositivity was not associated with a risk of non-Hodgkin lymphoma, although an increased risk was apparent for higher antibody levels for TSPyV in the subjects of one of the cohorts. However, a study with 199 AIDS-NHL cases and matched HIV infected controls found seropositivity for the TSPyV to be associated with a 1.6-fold increased risk of AIDS- related non-Hodgkin lymphoma (Halec et al., 2019). VP1 serology indicated that glioma risk was unrelated to infection with HPyV6 (Egan et al., 2021). In conclusion, all these studies show that infection with nHPyVs as determined by VP1 serology has limited value as a marker for cancer.

However, an increasIn conclusion, all these studies show that infection with novel HPyVs as determined by VP1 serology has limited value as a marker for cancer.

## Experimental Evidence for Oncogenic Properties of the Novel Human Polyomaviruses

### Novel Human Polyomaviruses and Cell Proliferation

A group headed by Becker examined the presence of MCPyV, HPyV6, HPyV7, TSPyV, HPyV9 and HPyV10 in 16 BRAF inhibitor-associated epithelial proliferation samples from six different patients. No or low DNA levels were detected for these HPyVs, except HPyV6, for which a relatively high HPyV6 DNA load and VP1 expression was monitored. The majority (10/16; i.e., 63%) of the BRAF inhibitor-associated epithelial proliferation samples contained mutations in the *RAS* gene, whereas six samples did not contain such mutations. The authors suggested that the high viral load and viral expression may contribute to the epithelial proliferation in the samples with wild-type *RAS* gene (Schrama et al., 2014). The expression of LT-ag and sT-ag was not analyzed in the samples and the mechanism of HPyV6-induced epithelial proliferation in wild-type *RAS* cells is not known. MWPyV LT-ag was unable to stimulate the growth of human diploid fibroblast IMR90 cells. The authors speculated that this was due to the instability of LT-ag (Berrios et al., 2015).

### Novel Human Polyomaviruses and Transforming Activity

Expression of TSPyV LT-ag in mouse fibroblast NIH3T3 cells induced colony formation in soft agar, underscoring the transforming properties of this LT-ag (Nako et al., 2020). In fact, colonies of TSPyV LT-ag were detected more frequently than in SV40 LT-ag-transfected cells, suggesting a higher transforming activity of TSPyV LT-ag compared to SV40 LT-ag, despite neglectable binding to p53.

An emerging role of HPyV7 in thymic epithelial tumors has been reported (Rennspiess et al., 2015; Klufah et al., 2021), and inactivation of the cyclin dependent kinase inhibitor 2A (also referred to as p16^INK^ or p14^ARF^) plays a role in progression of thymoma (Hirabayashi et al., 1997). Still, no correlation was found between the presence of HPyV7 LT-ag and p16 levels in thymic tumors (Keijzers et al., 2015).

Expression of MCPyV sT-ag in rat fibroblasts (Rat2 cell line) and rodent cells transformed cells *in vitro* (Shuda et al., 2011; Zhao et al., 2020). However, HPyV6 and HPyV7 sT-ag were unable to generate colonies in soft agar when expressed in Rat2 cells. Rather, expression of sT-ag of these two HPyVs in primary foreskin fibroblasts, in keratinocytes, in the lung adenocarcinoma cell line A549 and in BJ fibroblasts induced senescence (Zhao et al., 2020). MCPyV sT-ag contains the unique LKDYM sequence (aa 91-95), which is absent or poorly conserved in sT-ag of HPyV6, HPyV7 and the other nHPyVs (Supplementary Figure 2). This domain is referred to as the LT-ag stabilizing domain (LSD) and has been shown to inhibit the FBXW7 E3 ligase, resulting in the stabilization of MCPyV LT-ag and other FBXW7 substrates such as c-MYC and cyclin E (Kwun et al., 2013). The lack of this LSD in sT-ag of HPyV6, HPyV7 may explain their inability to stabilize c-MYC and to transform cells *in vitro* (Zhao et al., 2020). Nevertheless, senescent fibroblast can promote proliferation and metastasis of tumor cells through secretion proinflammatory factors known as senescence-associated secretory phenotype (Wang et al., 2020). Indeed, medium from HPyV6/7 sT-ag expressing fibroblasts could rescue proliferation of non-growing MCPyV LSD mutant sT-ag expressing cells (Zhao et al., 2020). These results indicate that the sT-ags of HPyV6 and HPyV7 may indirectly contribute to cancer by inducing senescence, resulting in the production of senescence-associated secreted cytokines, which may promote proliferation of cancer cells in the tumor microenvironment.

### Animal Models

To the best of our knowledge, apart from MCPyV, transgenic animal models expressing LT-ag and/or sT-ag of the nHPyVs have not been generated. The absence of a convincing association between most nHPyVs and human cancer may explain why scientists have been reluctant to generate transgenic animals. Xenograft studies with nHPyV-positive tumor cells are also lacking.

### Novel Human Polyomaviruses and Epigenetic Changes

Epigenetic changes, including DNA methylation, histone modification, chromatin remodeling, and expression of non-coding RNAs such as microRNA, long non-coding RNA and circular RNA play a crucial role in the regulation of gene expression, with aberrant epigenetic changes being characteristic for cancer cells [for reviews see e.g. (Baylin and Jones, 2016; Zhang et al., 2020)]. All human tumor viruses can trigger epigenetic changes in the host cell (Pietropaolo et al., 2021). SV40, BKPyV, JCPyV and MCPyV were found to provoke epigenetic changes (Balakrishnan and Milavetz, 2017; Pietropaolo et al., 2021). Epigenetic dysregulation seems to be a driving mechanism in MCPyV-positive MCC, with altered DNA methylation, histone and chromatin modifications, and microRNA regulation (Pietropaolo et al., 2021; Rotondo et al., 2021). Little is known about the effect of the nHPyVs on epigenetic changes in infected cells. Enhanced transcript levels of histone methyltransferases were measured in MCPyV sT-ag expressing BJ cells compared to HPyV6 and HPyV7 sT-ag expression cells, this coincided with increased trimethylation of histone H3 at lysine residue 4, which is associated with transcriptional activity (Venkatesh and Workman, 2015; Zhang et al., 2020). Recently, the group of Lui showed that the DnaJ domain of MCPyV T-antigens recruited Hsc70, which then binds to DICER mRNA, thereby leading to stabilization and increased protein expression. Because DICER1 is a key factor in microRNA biogenesis, the early proteins of MCPyV affects the expression of mature microRNAs (Gao et al., 2021). The DnaJ domain is highly conserved in the LT-ags and sT-ags of the other HPyV, hence suggesting that other HPyVs can affect the microRNA maturation process.

### The Effect of Novel Human Polyomaviruses on Signal Transduction Pathways

#### The Mitogen-Activated Protein Kinase Pathways

The mitogen-activated protein kinase (MAPK) pathways consist of the classical MAPK pathways referred to as the ERK (extracellular-regulated kinase), JNK (c-Jun N-terminal kinase) and p38MAPK pathways, in addition to the atypical pathways represented by ERK3/4, ERK7/8, and Nemo-like kinases (Cargnello and Roux, 2011). Few studies have addressed the possible effect of nHPyVs on MAPK pathways. The group of Tyring showed that ectopically expressed HPyV6 sT-ag as well as HPyV7 sT-ag in HEK293 cells interacted with PP2A, thereby leading to activation (phosphorylation) of the ERK components mitogen/extracellular signal-regulated kinases MEK1/2 and ERK1/2 (Wu et al., 2017b,2019b). Moreover, HPyV6 and HPyV7 sT-ags increased protein levels and phosphorylation levels of the transcription factor c-JUN. Mutant sT-ags unable to bind PP2A did not activate the MAPK pathway and failed to induce c-JUN phosphorylation. The sT-ag of the oncogenic MCPyV was also reported to bind PP2A (Griffiths et al., 2013; Kwun et al., 2015; Abdul-Sada et al., 2017; Cheng et al., 2017), but no ERK phosphorylation was observed in 42/44 Merkel cell carcinoma tumors with an unknown status of MCPyV, nor in HEK293 cells expressing MCPyV sT-ag (Houben et al., 2006; Wu et al., 2016c). TSPyV sT-ag was found to phosphorylate ERK, MEK and c-JUN when overexpressed in HEK293 cells, but whether this occurred in a PP2A-dependent manner was not examined (Wu et al., 2016d). The same group described that TSPyV MT-ag could interact with PP2A and activate the MEK/ERK cascade and phosphorylate the substrate MNK1, but not c-JUN in a PP2A-dependent manner (Wu et al., 2017a). Although not investigated, HPyV6, HPyV7, and TSPyV sT-ag may also activate the JNK pathway because c-JUN is also a substrate of this pathway (Cargnello and Roux, 2011). The biological effect of HPyV6 and HPyV7 sT-ags and TSPyV sT/MT-triggered MAPK activation was not investigated, nor has activation of this pathway and phosphorylation of c-JUN in HPyV6, HPyV7 and TSPyV positive cancers been examined. Given the known role of the aberrant MAPK pathway and c-JUN activity in cancer and inflammation (Shaulian, 2010; Cargnello and Roux, 2011; Moens et al., 2013) and the emerging role of HPyV6 and HPyV7 in human cancers (Klufah et al., 2021), HPyV6 and HPyV7 sT-ag may participate in pathological processes. The biological implication of the MWPyV sT:PP2A interaction was not investigated (Berrios et al., 2015).

#### Novel Human Polyomaviruses and the NFκB Pathway

The NFκB pathway plays a pivotal role in inflammation and contributes to immunity. This pathway is also involved in cancer development and progression (DiDonato et al., 2012; Xia et al., 2018) responses and is often targeted by viruses to evade the immune system. The central components of the NFκB pathway are members of the Rel family, which contains the c-Rel, RelA (or p65), RelB, NFκB1 (or p105/p50), and NFκB2 (or p100/p52). These proteins can form homo- and heterodimers that act as transcription factors, and can regulate the expression of several NFκB-responsive genes whose gene products are involved in inflammatory and immunological processes (Vallabhapurapu and Karin, 2009; Fullard et al., 2012). RelA/p65 and NFκB1/p105/p50 are activated in the canonical NFκB pathway, which has been linked to senescence, whereas RelB and NFκB2/p100/p52 are implicated in the non-canonical pathway, which bypasses cellular senescence (Vaughan and Jat, 2011; Capece et al., 2018). Human tumor viruses have evolved strategies to evade and exploit the NFκB signaling cascades for their benefit and to provoke cancer (Sun and Karin, 2008; Sun and Cesarman, 2011; Zhao et al., 2015; da Costa et al., 2016; Harhaj and Giam, 2018; Charostad et al., 2020). MCPyV sT-ag was shown to inhibit the canonical NFκB pathway, but to activate non-canonical NFκB signaling (Griffiths et al., 2013; Berrios et al., 2016; Zhao et al., 2020). Expression of HPyV6 or HPyV7 sT-ags in BJ cells activated the canonical NFκB pathway, whereas MCPyV sT inhibited this pathway. Vice versa, MCPyV sT-ag stimulated the non-canonical NFκB signaling, whereas HPyV6/7 sT had no effect (Zhao et al., 2020). The effect of sT-ags of other nHPyVs on canonical and non-canonical NFkB signaling has not been investigated. Proteins of other human oncoviruses can activate the non-canonical NFκB pathway, suggesting that this may be a common feature for transforming viruses (Sun, 2017).

Binding of viral dsDNA to the specific pattern recognition receptor toll-like receptor 9 (TLR9) results in activation of NFκB signaling and subsequent production of inflammatory mediators (Mogensen, 2009). While the early regions of KIPyV and WUPyV could inhibit TLR9 promoter activity in a transient transfection assay in the B lymphocyte RPMI-8226 cell line with a luciferase reporter vector, no reduction in TLR9 mRNA levels was observed in stable KIPyV (respectively, WUPyV) LT-ag/sT-ag expressing naturally immortalized keratinocytes compared to control cells (Shahzad et al., 2013). The reason for this cell-specific effect on TLR9 expression is not known nor have the natural host cells for KIPyV and WUPyV been identified, although evading the innate immune system may help facilitate KIPyV and WUPyV to establish a long-lasting viral infection, a prerequisite for a virus to possibly induce cancer. Whether other nHPyV can modulate TLR9 expression remains to be investigated.

#### Novel Human Polyomaviruses and the Phosphatidyl-3-Kinase/AKT/Mammalian Target of Rapamycin Pathway

The phosphatidyl-3-kinase (PI3K)/AKT/Mammalian Target of Rapamycin (mTOR) pathway controls cell proliferation, apoptosis, protein translation, and metabolic processes and is often constitutively activated in cancers (Sato et al., 2010). mTOR forms two complexes: mTORC1 and mTORC2 (Loewith et al., 2002; Jhanwar-Uniyal et al., 2019). mTORC1 regulates mRNA translation through phosphorylation of the eukaryotic initiation factor 4E-binding protein 1 (4E-BP1). In its non- or hyperphosphorylated form, 4E-BP1 binds eukaryotic initiation factor 4E (eIF4E), hence resulting in the inhibition of cap-dependent translation. However, hyperphosphorylated 4E-BP1 does not bind eIF4E, and therefore does not prevent translation of 5’ capped mRNA (Musa et al., 2016). Inducible expression of HPyV7 sT-ag in HEK293 cells enhanced phosphorylation of 4E-BP1 at Ser65, whereas overexpression of the sT-ags of TSPyV and HPyV6 had no effect on 4E-BP1 phosphorylation (Wu et al., 2015). HPyV7 sT-ag induced phosphorylation of 4E-BP1 in a PP2A dependent manner (Wu et al., 2019b). Interestingly, inhibition of mTOR increased BKPyV, JCPyV, MCPyV, HPyV7, and TSPyV LT-ag levels in HEK293 cells transfected with expression plasmids for these LT-ags. Stimulation of LT-ag levels by mTOR inhibitors was the result of an enhanced stability of the protein and was mediated by the inhibition of the SKP2E3 ligase, which targets LT-ag. Inhibition of mTOR activated JCPyV, MCPyV, HPyV7 and TSPyV replication (Alvarez Orellana et al., 2021). Thus, while mTOR inhibitors have been used as anti-cancer agent (Ciuffreda et al., 2010), it may not be suitable for the treatment of HPyV positive tumors as it may stabilize the LT-ag oncoprotein.

#### Novel Human Polyomaviruses and the Wnt Pathway

Perturbed Wnt signaling occurs often in cancer cells and human tumor viruses can dysregulate the Wnt pathway (van Zuylen et al., 2016; Zhan et al., 2017; Zhong et al., 2020). One of the major components of this pathway is β-catenin, which is normally cytoplasmic. Activation of the Wnt pathway leads to nuclear translocation of β-catenin, where it will bind to transcriptional activators and stimulate transcription of b-catenin target genes (Zhan et al., 2017). The JCPyV LT-ag binds β-catenin through an LT-ag central domain spanning residues 82 to 629 and promotes stabilization. Besides this, the LT-ag triggered β-catenin nuclear translocation, with subsequent enhancement of c-*myc* expression (Enam et al., 2002; Gan and Khalili, 2004; Ripple et al., 2014). Although the JCPyV LT-ag:β-catenin interaction was described in mouse medulloblastoma and in glioblastoma cell lines, it has been recently detected in human colorectal carcinoma, in which β-catenin and Wnt pathway are frequently increased (Enam et al., 2002). This suggests a role of JCPyV in colorectal malignancy through activation of the Wnt pathway, although JCPyV detection is frequent in both normal colorectal and colorectal cancer tissues [(Shavaleh et al., 2020; Shoraka et al., 2020); Supplementary Table 1]. Infection of human bladder cancer cells with BKPyV activated the β-catenin signaling pathway, but an interaction between LT-ag and β-catenin was not investigated (Zeng et al., 2020). Ectopic expression of SV40 sT-ag in HEK293 upregulated several genes encoding proteins involved in the Wnt signaling (Ali-Seyed et al., 2006). Several proteins of the Wnt pathway were overexpressed in MCC compared to carcinoid tumors of the lung, but the presence of MCPyV in MCC was not determined (Shao et al., 2013). It is unknown whether the early proteins of the nHPyVs interact with β-catenin or stimulate the β-catenin/Wnt pathway.

### Novel Human Polyomaviruses and Immune Evasion and Inflammation

The apolipoprotein B messenger RNA-editing, enzyme-catalytic, polypeptide-like 3 (APOBEC3) protein is a ssDNA cytosine-to-uracil deaminase that restricts viral replication as part of the innate immune response (Bonvin and Greeve, 2008; Romani et al., 2009; Cheng et al., 2021). APOBEC3 can also deaminate genomic DNA and is responsible for mutations in many different cancers (Burns et al., 2015). The LT-ags of BKPyV, JCPyV, and MCPyV were shown to upregulate expression and activity of APOBEC3 (Verhalen et al., 2016). The mechanism by which these LT-ags upregulate APOBEC3 is not known, nor has it been determined whether the LT-ags of other HPyVs have the same property. For human papillomavirus (HPV) it was shown that E6 upregulates APOBEC3 by inactivating p53. This may not be the case for HPyVs because MCPyV LT-ag cannot bind p53 (Cheng et al., 2013). Because expression of the enzyme is upregulated in HPV-related cancers (Warren et al., 2017), LT-ag-induced APOBEC3 expression may also contribute to HPyV-mediated tumorigenesis.

BKPyV- and JCPyV-encoded microRNA downregulates expression of the UL16 binding protein 3 (ULBP3), a protein recognized by the killer receptor NKG2D, thereby reducing the destruction of virus-infected cells by natural killer cells (Bauman et al., 2011). SV40 infection of the breast cancer MCF2 resulted in a decrease of ULBP1 levels (Bauman et al., 2016). Different from BKPyV and JCPyV, SV40 microRNA was not involved in the reduction of ULBP1 expression. The mechanism by which SV40 inhibits ULBP1 expression remains elusive. It remains unverified as to whether the nHPyVs can evade the innate immune system by modulating the expression of ULBP1 and/or ULBP3, nor has the existence of microRNA encoded by the nHPyVs been demonstrated.

### Novel Human Polyomaviruses and DNA Damage Response

DNA damage and chromosome instability is a hallmark of cancer (Hanahan and Weinberg, 2011). Cells have evolved a complex system, the DNA damage response (DDR), to detect and repair changes in the genome. The major pathways in DDR are the ataxia telangiectasia mutated kinase (ATM), the ATM-related and Rad3-related kinase (ATR), and the DNA-dependent kinase (DNA-PK) (Awasthi et al., 2015; Blackford and Jackson, 2017). BKPyV, JCPyV, and MCPyV early proteins can interfere with the DDR pathway to support viral propagation at the cost of host genome stability (Justice et al., 2015; Moens and Macdonald, 2019; Tahseen et al., 2020). Less is known about the effects of the nHPyV on the DDR. The sT-ags of HPyV6, HPyV7, and TSPyV provoke phosphorylation of ATM and its downstream effector check point kinase 2 (CHK2), which in turn phosphorylates histone H2 variant H2AX and tumor promoter p53 binding protein 1 (p53BP1) (Wu et al., 2019a). A possible link between HPyV6, HPyV7, or TSPyV-induced activation of the DDR and oncogenesis is not known.

## Conclusion and Further Directions

All PyVs encode LT-ag and sT-ag, proteins that have been shown to be oncogenic in cell culture and animal models for some of the PyVs. Some PyVs, including the HPyV MCPyV, can cause cancer in their natural host. A causal role for HPyV6 and HPyV7 in human skin cancers is emerging, but the association of other nHPyVs with human tumors is less obvious (Klufah et al., 2021). Table 2 summarizes arguments pro and contra a role for the nHPyVs in cancer. Several explanations can be suggested as to why at present these viruses do not seem to cause cancer. Although these viruses may establish a persistent infection, the expression levels of LT-ag and sT-ag may be too low to be harmful for the cell. Although the LT-ags and sT-ags of the nHPyVs can potentially bind tumor suppressors like pRb, p53, and PP2A, their interaction may not occur *in vivo*. One of the characteristics of MCPyV-positive MCC tumors is integration of the viral genome (Feng et al., 2008). Integration of the BKPyV genome has also been reported in urinary tract cancers (Starrett and Buck, 2019). The lack of integration and/or the expression of a truncated LT-ag by nHPyVs may also explain their failure to transform infected cells. Finally, the tumors associated with nHPyVs has not been identified.

**TABLE 2:** Indication pro and contra for a role of the novel HPyVs in cancer.

Pro	Contra
Putative or proven conserved ● p53 binding motif ● pRb binding motif ● PP2A binding motif ● DnaJ motif ● Cul7 motif	Absent or rare detection of viral sequences and protein in tumors
Activation of oncogenic signaling pathways *in vitro*	Incidence in tumor tissue comparable with normal tissue
Infection early in life: long incubation time	Low viral genome copy numbers in tumors
Alternative early proteins with similarity to the MT oncoprotein of other PyVs	No correlation between LT-ag/sT-ag seropositivity and cancer
TSPyV LT-ag has transforming properties *in vitro*	Viral oncoproteins do not induce cell proliferation *in vitro*
	HPyV6 and HPyV7 sT-ag lacks transforming properties *in vitro*

Cell culture and transgenic animal studies investigating the transforming capacity of LT-ag and sT-ag are required to explore the possible tumorigenic potentials of nHPyVs. The interaction with p53, retinoblastoma proteins, PP2A and other tumor suppressor proteins should be tested. Epidemiological studies on more and different tumor samples (the genuine tumors associated with nHPyVs may yet not have been investigated) should be examined for the presence of viral sequences and proteins. The viral genome copy number and possible integration in tumors may also indicate whether the nHPyVs are involved in cancer or not. Normal adjacent tissue should be included as control. Immunodeficiency of the host may enhance the activity of the virus and hence contribute. Consequently, nHPyVs may play a contributing role in immunocompromised patients who develop cancer. Seropositivity against LT-ag and sT-ag in HPyV-positive and –negative cancer patients and healthy controls may also provide a clue on the plausible implication of these viruses in cancer.

In conclusion, convincing evidence is currently lacking for a causal role of nHPyVs in human cancers, although a hit-and-run mechanism, as suggested for MCPyV, cannot be excluded (Houben et al., 2012). Transient expression of their oncoproteins LT-ag and sT-ag may initiate neoplastic processes resulting in tumor formation without evidence for the presence of the virus at a later stage. Further research is required to unambiguously determine whether these nHPyVs can contribute to the development of cancer.

## Author Contributions

VP, CP, and UM: conceptualization, writing—original draft preparation, and writing—review and editing. All the authors have read and agreed to the published version of the manuscript.

## Conflict of Interest

The authors declare that the research was conducted in the absence of any commercial or financial relationships that could be construed as a potential conflict of interest.

## Publisher’s Note

All claims expressed in this article are solely those of the authors and do not necessarily represent those of their affiliated organizations, or those of the publisher, the editors and the reviewers. Any product that may be evaluated in this article, or claim that may be made by its manufacturer, is not guaranteed or endorsed by the publisher.
