# Supplementary material for: Functional Domains of the Early Proteins and Experimental and Epidemiological Studies Suggest a Role for the Novel Human Polyomaviruses in Cancer
Source: Front Microbiol. 2022 Feb 18;13:834368. doi: 10.3389/fmicb.2022.834368 (PMC8894888; doi:10.3389/fmicb.2022.834368)
Supplement: Supplementary file 1 [file Table_1.DOC]

Alignment LT

**>MPyV (J02288): 785 aa**

MDRVLSRADKERLLELLKLPRQLWGDFGRMQQAYKQQSLLLHPDKGGSHALMQELNSLWGTFKTEVYNLRMNLGGTGFQGSPPRTAERGTEESGHSPLHDDYWSFSYGSKYFTREWNDFFRKWDPSYQSPPKTAESSEQPDLFCYEEPLLSPNPSSPTDTPAHTAGRRRNPCVAEPDDSISPDPPRTPVSRKRPRPAGATGGGGGGVHANGGSVFGHPTGGTSTPAHPPPYHSQGGSESMGGSDSSGFAEGSFRSDPRCESENESYSQSCSQSSFNATPPKKAREDPAPSDFPSSLTGYLSHAIYSNKTFPAFLVYSTKEKCKQLYDTIGKFRPEFKCLVHYEEGGMLFFLTMTKHRVSAVKNYCSKLCRSFLMCKAVTKPMECYQVVTAAPFQLITENKPGLHQFEFTDEPEEQKAVDWIMVADFALENNLDDPLLIMGYYLDFAKEVPSCIKCSKEETRLQIHWKNHRKHAENADLFLNCKAQKTICQQAAASLASRRLKLVECTRSQLLKERLQQSLLRLKELGSSDALLYLAGVAWYQCLLEDFPQTLFKMLKLLTENVPKRRNILFRGPVNSGKTGLAAALISLLGGKSLNINCPADKLAFELGVAQDQFVVCFEDVKGQIALNKQLQPGMGVANLDNLRTTWNGSVKVNLEKKHSNKRSQLFPPCVCTMNEYLLPQTVWARFHMVLDFTCKPHLAQSLEKCEFLQRERIIQSGDTLALLLIWNFTSDVFDPDIQGLVKEVRDQFASECSYSLFCDILCNVQEGDDPLKDICDIAEYTVY

**>HaPyV (NC_001663): 751 aa**

MDRILTKEEKQALISLLDLEPQYWGDYGRMQKCYKKKCLQLHPDKGGNEELMQQLNTLWTKLKDGLYRVRLLLGPSQDPNASTSTSRPGEFYNPDTGGYWSYSYGSAGYSDQQKKYWEEFFSKWDVNEDLTCQEELSSSEDEFTPWHPNPPPSPVSISSDSSSSSCDEEYPRNSSRKRKRVHANGSPNTPIQPNKRAHTPGGGRTTIRGDTDIPRTPARESQSTFGSYFNSTEELEEEISQTQQSHHNTTPKKPPPTVSPDDFPTILRGFLSHAIFSNKTQNAFIIYSTKEKCEVLYEQIDKYNPDYKGIFIMKQTEAFVMFMTPGKHRVAAVKSYCCKFCTVSFLLCKAVTKPLELYNCVAKCDDFQILKENKPGLYHFEFCDEKKEVKQIDWNFLTSFAVENELDDPLVIMGHYLEFSQCESSCKKCAEALPRMKVHWANHSQHLENAELFLHCKQQKSICQQAADNVLARRRLKVLESTRQELLAERLNKLLDQLKDLSPVDKHLYLAGVAWYQCMFPDFEMMLLDILKLFTENVPKKRNVLFRGPVNSGKTSLAAAIMNLVGGVALNVNCPADKLNFELGVAIDKFAVVFEDVKGQTGDKRHLQSGLGINNLDNLRDYLDGSVKVNLEKKHVNKRSQIFPPCIVTANEYFFPQTLYARFHKVYNFEVKDFLAKSLEENSYMGRHRVCQSPLTMLIALLWNVPTENFDKSLKEKVETEKKVLSDMCNFTTFAEMCLNIQRGADPLEAL

**>SV40 (NC_001669): 708 aa**

MDKVLNREESLQLMDLLGLERSAWGNIPLMRKAYLKKCKEFHPDKGGDEEKMKKMNTLYKKMEDGVKYAHQPDFGGFWDATEIPTYGTDEWEQWWNAFNEENLFCSEEMPSSDDEATADSQHSTPPKKKRKVEDPKDFPSELLSFLSHAVFSNRTLACFAIYTTKEKAALLYKKIMEKYSVTFISRHNSYNHNILFFLTPHRHRVSAINNYAQKLCTFSFLICKGVNKEYLMYSALTRDPFSVIEESLPGGLKEHDFNPEEAEETKQVSWKLVTEYAMETKCDDVLLLLGMYLEFQYSFEMCLKCIKKEQPSHYKYHEKHYANAAIFADSKNQKTICQQAVDTVLAKKRVDSLQLTREQMLTNRFNDLLDRMDIMFGSTGSADIEEWMAGVAWLHCLLPKMDSVVYDFLKCMVYNIPKKRYWLFKGPIDSGKTTLAAALLELCGGKALNVNLPLDRLNFELGVAIDQFLVVFEDVKGTGGESRDLPSGQGINNLDNLRDYLDGSVKVNLEKKHLNKRTQIFPPGIVTMNEYSVPKTLQARFVKQIDFRPKDYLKHCLERSEFLLEKRIIQSGIALLLMLIWYRPVAEFAQSIQSRIVEWKERLDKEFSLSVYQKMKFNVAMGIGVLDWLRNSDDDDEDSQENADKNEDGGEKNMEDSGHETGIDSQSQGSFQAPQSSQSVHDHNQPYHICRGFTCFKKPPTPPPEPET

**>BKV (NC_001538): 695 aa**

MDKVLNREESMELMDLLGLERAAWGNLPLMRKAYLRKCKEFHPDKGGDEDKMKRMNTLYKKMEQDVKVAHQPDFGTWSSSEVPTYGTEEWESWWSSFNEKWDEDLFCHEDMFASDEEATADSQHSTPPKKKRKVEDPKDFPSDLHQFLSQAVFSNRTLACFAVYTTKEKAQILYKKLMEKYSVTFISRHMCAGHNIIFFLTPHRHRVSAINNFCQKLCTFSFLICKGVNKEYLLYSALTRDPYHTIEESIQGGLKEHDFSPEEPEETKQVSWKLITEYAVETKCEDVFLLLGMYLEFQYNVEECKKCQKKDQPYHFKYHEKHFANAIIFAESKNQKSICQQAVDTVLAKKRVDTLHMTREEMLTERFNHILDKMDLIFGAHGNAVLEQYMAGVAWLHCLLPKMDSVIFDFLHCIVFNVPKRRYWLFKGPIDSGKTTLAAGLLDLCGGKALNVNLPMERLTFELGVAIDQYMVVFEDVKGTGAESKDLPSGHGINNLDSLRDYLDGSVKVNLEKKHLNKRTQIFPPGLVTMNEYPVPKTLQARFVRQIDFRPKIYLRKSLQNSEFLLEKRILQSGMTLLLLLIWFRPVADFATDIQSRIVEWKERLDSEISMYTFSRMKYNICMGKCILDITREEDSETEDSGHGSSTESQSQCSSQVSDTSAPAEDSQRSDPHSQELHLCKGFQCFKRPKTPPPK

**>JCV (NC_001699): 688 aa**

MDKVLNREESMELMDLLGLDRSAWGNIPVMRKAYLKKCKELHPDKGGDEDKMKRMNFLYKKMEQGVKVAHQPDFGTWNSSEVPTYGTDEWESWWNTFNEKWDEDLFCHEEMFASDDENTGSQHSTPPKKKKKVEDPKDFPVDLHAFLSQAVFSNRTVASFAVYTTKEKAQILYKKLMEKYSVTFISRHGFGGHNILFFLTPHRHRVSAINNYCQKLCTFSFLICKGVNKEYLFYSALCRQPYAVVEESIQGGLKEHDFNPEEPEETKQVSWKLVTQYALETKCEDVFLLMGMYLDFQENPQQCKKCEKKDQPNHFNHHEKHYYNAQIFADSKNQKSICQQAVDTVAAKQRVDSIHMTREEMLVERFNFLLDKMDLIFGAHGNAVLEQYMAGVAWIHCLLPQMDTVIYDFLKCIVLNIPKKRYWLFKGPIDSGKTTLAAALLDLCGGKSLNVNMPLERLNFELGVGIDQFMVVFEDVKGTGAESRDLPSGHGISNLDCLRDYLDGSVKVNLERKHQNKRTQVFPPGIVTMNEYSVPRTLQARFVRQIDFRPKAYLRKSLSCSEYLLEKRILQSGMTLLLLLIWFRPVADFAAAIHERIVQWKERLDLEISMYTFSTMKANVGMGRPILDFPREEDSEAEDSGHGSSTESQSQCFSQVSEASGADTQENCTFHICKGFQCFKKPKTPPPK

**>KIPyV (NC_009238): 641 aa**

MDKTLSREEAKQLMQLLCLDMSCWGNLPLMRRQYLVKCKEYHPDKGGNEESMKLLNSLYLKLQDSVSSVHDLNEEEDNIWQSSQIPTYGTPDWDEWWSQFNTYWEEELRCNESMPSSPKRSAPEEEPSCSQATPPKKKHAFDASLEFPKELLEFVSHAVFSNKCITCFVVHTTREKGEVLYKKLLQKYQCSFISKHAFYNTVLIFFLTPHKHRVSAINNFCKGHCTVSFLFCKGVNNPYGLYSRMCRQPFNLCEENIPGGLKENEFNPEDLFGEPKEPSLSWNQIANFALEFDIDDVYYLLGSYIRFATKPEECEKCSKNDDATHKRVHVQNHENAVLLQESKSQKNACTQAIDRVIAERRYNCLTLTRKKLLTKRFKKLFNEMDKIVVGERKILLYMASIAWYTGLNKKIDELVVRFLKLIVDNKPKHRYWLFKGPINSGKTTLATALLNLCGGKALNINIPSEKLPFELGVALDQYMVVFEDVKGQIGIEKQLPSGNGVNNLDNLRDYLDGCVEVNLEKKHVNKRSQIFPPGIVTMNEYCIPETVAVRFEKTVMFTIKRNLRESLEKTPQLLSQRILHSGIAMLLLLIWYRPVSDFDEEIQSNVVYWKEVLDNYIGLTEFATMQMNVTNGKNILEKWFE

**>WUPyV (NC_009539): 648 aa**

MDKTLSRNEAKELMQLLGLDMTCWGNLPLMRTKYLSKCKEFHPDKGGNEEKMKKLNSLYLKLQECVSTVHQLNEEEDEVWSSSQIPTYGTPDWDYWWSQFNSYWEEELRCNEEMPKSPGETPTKRTREDDEEPQCSQATPPKKKKDNATDASLSFPKELEEFVSQAVFSNRTLTAFVIHTTKEKAETLYKKLLSKFKCNFASRHSYYNTALVFILTPFRHRVSAVNNFCKGYCTISFLFCKGVNNAYGLYSRMTRDPFTLCEENIPGGLKENDFKAEDLYGEFKDQLNWKALSEFALELGIDDVYLLLGLYLQLSIKVEECEKCNSNEDATHNRLHMEHQKNALLFSDSKSQKNVCQQAIDVVIAKRRVDSLNMSREDLLARRFEKILDKMDKTIKGEQDVLLYMAGVAWYLGLNGKIDELVYRYLKVIVENVPKKRYWVFKGPINSGKTTVAAALLDLCGGKALNINIPADRLNFELGVAIDQFTVVFEDVKGQVGDNKLLPSGNGMSNLDNLRDYLDGSVKVNLEKKHLNKRSQIFPPGIVTMNEYLVPATLAPRFHKTVLFTPKRHLKESLDKTPELMVKRVLQSGMCILIMLIWCRPVSDFHPCIQAKVVYWKELLDKYIGLTEFADMQMNVTNGCNILEKHNA

**>MCPyV (NC_010277): 817 aa**

MDLVLNRKEREALCKLLEIAPNCYGNIPLMKAAFKRSCLKHHPDKGGNPVIMMELNTLWSKFQQNIHKLRSDFSMFDEVDEAPIYGTTKFKEWWRSGGFSFGKAYEYGPNPHGTNSRSRKPSSNASRGAPSGSSPPHSQSSSSGYGSFSASQASDSQSRGPDIPPEHHEEPTSSSGSSSREETTNSGRESSTPNGTSVPRNSSRTDGTWEDLFCDESLSSPEPPSSSEEPEEPPSSRSSPRQPPSSSAEEASSSQFTDEEYRSSSFTTPKTPPPFSRKRKFGGSRSSASSASSASFTSTPPKPKKNRETPVPTDFPIDLSDYLSHAVYSNKTVSCFAIYTTSDKAIELYDKIEKFKVDFKSRHACELGCILLFITLSKHRVSAIKNFCSTFCTISFLICKGVNKMPEMYNNLCKPPYKLLQENKPLLNYEFQEKEKEASCNWNLVAEFACEYELDDHFIILAHYLDFAKPFPCQKCENRSRLKPHKAHEAHHSNAKLFYESKSQKTICQQAADTVLAKRRLEMLEMTRTEMLCKKFKKHLERLRDLDTIDLLYYMGGVAWYCCLFEEFEKKLQKIIQLLTENIPKYRNIWFKGPINSGKTSFAAALIDLLEGKALNINCPSDKLPFELGCALDKFMVVFEDVKGQNSLNKDLQPGQGINNLDNLRDHLDGAVAVSLEKKHVNKKHQIFPPCIVTANDYFIPKTLIARFSYTLHFSPKANLRDSLDQNMEIRKRRILQSGTTLLLCLIWCLPDTTFKPCLQEEIKNWKQILQSEISYGKFCQMIENVEAGQDPLLNILIEEEGPEETEETQDSGTFSQ

**>HPyV6 (NC_014406): 669 aa**

MDRLLAREEVRELMDLIGLSMACWGNLPLMQQKIRLACKKYHPDKGGDPEKMQRLNVLKEKLNATLRDQMSSSPTWCFSSEVRPPPQYGSPGWEQWWADFNRGWDDEDLYCDEHLSASEEEDNVDPGEGNSQDSKYSCTPPKKRKPNPAPNDFPSCLHDYLSHATLGNKCYTCFVSYTTLEKWETLYDKLQSAFNAVFTGAYKCNDNTGAILYCITPRRHRVSAMLNALSKCCTISFLLIKAVLKSAECYMALQGDEFTVIQESRAEGLHSYDFQEGSKKEECDWNQVASFASDTDLTDCLALLGYYIEFANDPASCMKCKKGVKVHKHHEVHFHNAQLFKAAKNQKSIAQQACDRVAAQRRVLMLESTRQDLVLQAFKKQFTILAEQYAGGVEITQLLGAVAWLDCLQPSFTTKLKEILSILTENIPKKRNVLFKGPINSGKTTLAAAILDLVGGVSLNVNCTPDKINFELGCAIDKFMCVIEDVKGTPMANTNLTQGCGMTNLDNLRDYLDGCVPVNMERKHLNKVSQLFPPSVITCNDYIIPCTVKARIARGYYFLHKPCLQKCLKDCVLMSKRLLQKGTTLLAALIWWEPVEDFMEELQEDVVNWKQTFERWVSFGMYQTMKENILAGIDPFTNVLVDESFVQPQENDETNDSTQESGIGSMHSM

**>HPyV7 (NC_014407): 671 aa**

MDKLLGRDEVKELMELIGLNMACWGNLPLIQHKVRLASKKYHPDKGGDPQKMQRLNVLKDKLQATLRDQRSGSPMWHYSSDEVRPPPPYGSPAWDQWWQDFNKGWDEDLYCTEELSSSDEEEPAASASVNPEEGCSQDSKYSATPPKQKKPNPAPQDFPECLSEFLSHATLGNKCYTCFLCYTTYEKSMLLYEKLGVEFNALFIGAYNCVDGSGALVFFISGSRHRVSAILNACKKHCTVSFIMVKAVLKNAECYKALQDSKFAVLRESKEGGLHSYDFQEASKKDDCDWNFVADFAADMELTDVLLIMGYYMEFATEPSLCPKCLKSVKAHQHHEKHWANAKLFKTAKNQKGIAQQAADRVLAARRVLMMESTRKDLMVMSFKKQFKVLAEQFGGGVEITQLIGAVAWLDCLLPQFTIKIKEMLSYLVENTPKRRNLLFKGPINSGKTTLAAAILDLLGGVALNVNCSSDKINFELGCAIDKYMVVIEDVKGTPLPNTDLPSGVGMANLDNMRDYLDGCVPVNLERKHINKTSQLFPPCIITCNEYAIPTTVKARVAKGYYFLHKPGLKKSLDANPILMKKRLLQKGCTLLAALIWWEPVSDFVEEIQEEVVNWKQTFEQWVSYGMFQTMKENILSGKDPFEGVLINDPTEENTRETQESTESGIGSMNN

**>TSPyV (NC_014361): 697 aa**

MDKFLSREESLELMDLLQIPRHCYGNFALMKINHKKMSLKYHPDKGGDPEKMSRLNQLWQKLQEGIYNARQEFPTSFSSQHDVPTQDGRDIPPYGHPSWASWWESFNQEWDNLFDTMQDPDLFCHESTIPSDESRSPSPTPGPSTQFSEENSRRRRAAPPEDSPGCTQSSFSATPPKPKKSKYDSVPNDFPDMLRPFLSNAVYSNKTLSSFLIYTTNEKAEYLYKKLDKFNPEFKSRHSFQEGSMVFLMTPGKHRVSAIKNLCVTHCTVSFLLCKAVIKQVECYRCMCSEPFKLLEESKPGIFEYEFNEENGKPVVNWNLLTDFAVTNRLDDPLLIMAHYLDFAEEPSICSKCTKKALKAHYNYHSLHHKNAKLFKECKTQKTACQQAADVVMAKQRLKLIESTRKELLEERFKLMFEKLTDEFGQIKILQYMAGVAWYSCLFENIDEVVTKILKLIVENVPKKRNCLFRGPINSGKTTFAAALMNFLGGKTLNVNCPADKLPFELGCAIDQFVVIFEDVKGQIALNKKLQPGQGVSNLDNLRDHLDGSVKVNLERKHVNKRSQIFPPCLVTMNEYLLPETIFTRFAYVLNFTPKHNLRSCLQVSDYLLTERILQDGVTIALLLVWYCPITMFSESIKEDVKYWKDILCKYMGHTNFATLLLNVEEGKDPLDSVVIEVEDEEEEEFSETNDSGFQTQ

**>HPyV9 (NC_015150): 680 aa**

MDQTLSLEERNELMDLLQLTRAAWGNLSLMKKAYKTVSKIYHPDKGGNPEKMQRLNELFQKLQVTLLEIRSNCGSSSSQGYYSDSPYFTETPFSYCERKNEDPEGGSWGKWWREFVNKEYDDLFCSETISSSDDENNPGPSAPPPSSASASEDPDPEEEAGSSQSSFTCTPPKRKKPEPNTPEDFPMCLYSFLSHAIYSNKTMNCFLIYTTVEKSKQLYRTVEKSKIKVDFKAIFLYKDDGIEGGLLYFITLGKHRVSAVKHFCVAQCTFSFIHCKAVIKPLELYRALGKPPFKLLEENKPGVSMFDFQEEKEQAVNWQEICNYAVEAKITDVLLLLGIYLDFAVEPGTCSKCEKKSHKFHYNYHSKHHANACLFLESKSQKNICQQAVDQVLAAKRLKLVECTRMELLEDRFIQLFDEMEDFLHGEIEILRWMSGVAWYTILLDNSWDVFQKILQLVTTSQPKKRNILFKGPINSGKTTLASAFMHFFDGKALNINCPAEKLSFELGCAIDQFCVLLDDVKGQITLNKHLQPGQGVNNLDNLRDHLDGTIKVNLEKKHVNKRSQIFPPVIMTMNEYLLPPTVGVRFALHIHFHCKTYLKQSLEKSDLIEKRILNSGYTILLLLLWYNPVDSFTPKVQEYVVKWKEILERHVSITQFGNIQQNILDGKDPLHGIVIEEQA

**>HPyV10(JX262162)**

MDRVLSRDEVKELMALLSLNTAAWGNIPLMQYKYRQTCLKLHPDKGGDGEKMKRLNELFSKMYTTIEKLRREGEVYFPAKGNPTYGTPEWDQWWEEFNRGWDEDLSCNESFAPSDEEEPGPSQSASQTANDTNTPKKRPRESSSNSTCTPPKRPRNFNPVDFPEVLLEFLSNAIFSNKTLNSFVLYTTREKGQFLYEKVPLKFKAMFYSLHEFDGDSLLFLLLSGKHRVSAIKNYCSNLCTVSFLLVKGCLKAYECYYALCKTPFKLIKQSQEHGLSKTDFCEEEKDKVVNWQQICEFAVEVQCEDPLLLMGMLLDFAKDVEGCSKCEQKKLKHHYKFHEAQNINSKLFKDCKNQKTICQQATDWVTAQRRLLILESTREHLLVLRFKHMFEKMEDICGEVEICQYMAGVAWLSLLMPHFDEIILFIIKAMTENVPKRRYVLFKGPINSGKTTVAAAILDLLGGKTLNVNCPPDKLAFEIGCAIDEYMVVFEDVKGQNEGSNSSLTPGMGMSNLDNLRDHLDGCVKVNLEKKHVNKKSQIFPPGIITMNDYFIPPTLQARMIKTINFRPKLFLRNSLEKNSELLRKRIVQSGVTLLLLLCWWQPVIAFHPEIHDNVRYWKETIEKYVPFGMYHDIRRNIESGEDPLKDILICVDADEDTQQDSGINSQ

**>STLPyV (KF525270)**

MDQALSREEAKELMGLLGLPEDSWGNVPLITYRFRQKSKIYHPDKGGNEETMKRMTELYSRMQNTLQNLRSSNENEHMYPPGGQYGTPAWEQWWEEFNQPFEDDLTCNESFNCSDDEGTSASQKRKFPDYSTQNSTPPKKNKPADPTDFPAELETFLSHAVFSNKTSNCFCIYTTMEKGNELYTVIGPKFKSMFISCHSYNTCCLLFMILAGKHRVSALKNFCSALCSISFVLVKSCLKPYECYYRMCSSPFSVIKQSRPEGLSQAEFMEQENSKPTVNWQQICEFAVQFNCEDPLLLMGIYLDFSESPDNCEKCRTELKHHNQFHEKEHNNAKLFRDSKTQKTLCQQACDWVCAKRRVLILESTREDLLVIRFKQVLKEMQDIAGEVEILRYMAGVAWLSLLFNHFDDIVLEIIRTMVVNTPKRRYFLFKGPINSGKTTVAAAILDLLGGRTLNINCPPEKVNFELGCAIDEFMVVFEDVKGQTEGKTNLTSGMGMNNLDSLRDHLDGCVKVNLEKKHLNKRSQIFPPGIITMNEYNVPLTILARMVKVINFRPKHYLKKSLEVNNELLHRRIVQSGKTLLMLLMWWQPVKVFHSSIHEDVKLWKDTLTKYVSIGMFHDIQKNIQNGEDPLKNILICEDTENNETQDSAFCTQDSDNE

**>HPyV12 (NC_020890)**

MDSILTFAERQLLISLLKISGDTFGNVPAMARAYKLAAKRLHPDKGGNEAEMKKLNELWNKFKDGIYNLREYNPHRNPSGNPCGSFFWFRRFQSDLFADETLSSTSSDEEPEPAQRKRGTGANIHESASRTSFSTGSPGKGTRGGGGIPRDAPPPDSGYGSFPFDSTPPKRGRNGGGSAPSTSGGVPDDFEGDSADQNCSQATPPKSKKAKMDNGPSDFPCDINIFLSSAVYSNKTVNAFLIFTTVEKCQLLYQKIDVKFKIDFKSRHEGENKAHGYLYILTVAKHRVSAVKNYCAKQCTISFLHCKAINKPFDCYKALCCDPYKRIESNKDLFQTDFENENSQQVDWTLISTFAECNMIDDPYLIMGHYLDFASPLPCNKCQMKVLKVHYQFHEAHHNNAILFKNSKAQKTICQQAADVVIAKRRLHLIESTREELLAERFKLFLNKYKELDKMRVLEHMAGVMWYSVMFENIDRIVIQILKLMTENIPKKRNVLFKGPINSGKTSFAAAMLDLISGKTLNINCPADKLPFELGCALDQFAVVFEDVKGQVGNDKTLQCGQGVNNLDNIRDHLDGSVTVNLEKKHVNKKTQIFPPCIVTMNDYKLPPTVKARFAYMVIFTHMKCLQTSLEKNDDIVKHRITHSGLTMFMILMWYCSSSAFIPSLRETIEIEKKLLESICTTEIACLMKDNIKAGRDPLHDIVTEADE

**>NJPyV-2013 (NC_024118)**

MEKVLEKSDKEMLIELLGIPRYAYGNFPIMKTAYKRASKIYHPDKGGSSEKMMLLNSLWQKFQEGLIEVRDSEVFSDSYGSANFRKRYASWCSSVFTNEKSDSRADLHCDESPISSSSDEEDETQSSGYNSFPFTSTPTPSTSTASQEVPPPFSEPQFPESSSASGSSSAGRNTETERESPPKRRRGTEDLDGSYTDSQTSFASTPPKQKRKSPDSPSDLPSCLFDFVSHAIFSNKTVNAFILYSTLEKASLLYEKIDKFKIEFKSLHKLTEGANVGGGLVLVMTIAKHRVSAMKNFCQQFCTVSFLICKVVLKPLECYQCLCKPPFSQVKANKDGLFSYDFEDKKEENCNWNKVAEFAVLADIDDPLLILAHYLDFAQPFPCLKCEHQKTKAHDYHKAHHENAVLFEACKSQRSICNQASDIVLAKRRLLLTESTREELLAMCFQKQLKALQALDTLEIYDHMAGVAWYANLFENFDDILFQILKLLTQNIPKQRNILFRGPVNSGKTTFAAALVDLLGGRSLNVNCPADKLNFELGCAIDRFFVVFEDVKGQNMLNKKLQPGQGISNLDNMRDYLDGAVPVNLEKKHMNKRSQVFPPCVMTMNEYFMPQTLFVRFSLKLDFVSRPNLQSAVDKTPGLVANRILQKGLTLFLLLIWYTPVKKFAVSLQEEIANWKCIIEKTVSHSDFCKMLENIEVGESPLTDLIDEGDN

**>LiPyV (NC_034253)**

MDAVLTTPERRQLCLLLDISPQEYGNIPLMKNAFKKACLKHHPDKGGDPVLMMQLNSLWGKFTTSLTEARASTYQDDPIYGTPQFRAWWYRKHYGFFPDGFDPRRSSSTRNRRPGGTEEPEYEQPSTSGPNLSTPRPKKSRSNLFGSSGCRSRSTAQNPLFCDESLSSSEEEAENASAKSQSDHFSFTSQEESSQASAPSFTSNESTPASTPKRNRKNQSFGGIPSPGSRRSFSSTPPKQKRYKEGDDPIDFPNCLSEFLSHATLSNKTYSCFLIFTTAEKGELLYNKVSEKYKVEFKSLHNYRGGTALLFLVLLTRHRVTAIKNFACTFCSVSFLLCKAVIKSPELYSCLIKEPFCLLKENKPGLWDHEFAENKEPSCNWNLVADFACNYNLTDWVIILAHYLDFANDPALCDKCSKLPLKPHEAHRKNYENAKFFLKCKSQKTICQQAADVVIAKNRLKMLEQTREEMLREKILCKLKELQEMKLETLYIFLAGVAWYKVMFGNFEWKVFKVLNLLTDNIPKKRNVLFKGPINSGKTSLAAAFLDLLEGKALNINCPQDRLNFELGCAQDLFMVCFEDVKGSRGQNKDLPSGQGMHNLDNLRDHMDGSVNVNLEKKHQNKRSQIFPPSITTCNEYIIPDTVMCRFAITITFAHKENLRTSLRKNIDMQKLRVLQRGCTLLLGLMWLLPKEKFDDEIRPEVERWRDAFRGDIPQAHFEKMIQNVECGLDPLEDLFVEAPADAPPAAAPEDSEAEPPVASKVPHQHENTMEEPERQQKARPEKDLESQDSGLFTQDSGQT

**>QPyV (BK010702)**

MDRLLSRDEVNELMQLIGLSMSNWGNLPLIQHKVREACKKHHPDKGGDPEKMQRLNVLKDKFAATMRDQSSGNPIWHFSSEEVRPPPPYGTPEWDKWWHDFNRGWDEDLYCTEELSASDEEQTAEDPEEGCSQNSKYSATPPKQRKPNPAPQDFPECINEYLSHATLGNKCYNCFVCYTTMEKSLMLYDKLNNEFNALFIGNYKCNDGSGSIVYMITGSRHRPSAILNASKKYCTVSFSLVKAVLKNAECYKALQGPNFTVIRESREGGLHSYDFQEASKKDDCDWNAVAEFALANDLTDPLLIMGYYLEFAAEPSLCQKCKKGVKAHKCHELQWSNAKLFKSAKNQRGIATQAADRVLSARRVMMIESTRVDLMVMAFKKQFQVLNDQFAGGVEITQLLGAVAWLDCLMPSFTTKLKEMLTLLVQNYAKKRNLLFKGPINSGKTTVAAGIMDLLGGVALNVNCSSDKINFELGCAIDKMLVVFEDVKGQPLPNTDLPAGVGMANLDNLRDHLDGCVPVNLERKHTNKVSQLFPPCIITCNDYAIPRTVKARVAKGYYFIHKPNLKKCLDVNPILMQKRLLQKGVTLLAALIWWEPVSEFVEEIQEDVVNWKQTFERWVTYGMYQDMKQNILAGKDPFYGVIMSDINEIVEETQESTESGVGSMET

CLUSTAL O(1.2.4) multiple sequence alignment

HPyV6 MDRLLAREEVRELMDLIGLSMACWGNLPLMQQKIRLACKKYHPDKGGDPEKMQRLNVLKE 60

HPyV7 MDKLLGRDEVKELMELIGLNMACWGNLPLIQHKVRLASKKYHPDKGGDPQKMQRLNVLKD 60

QPyV MDRLLSRDEVNELMQLIGLSMSNWGNLPLIQHKVREACKKHHPDKGGDPEKMQRLNVLKD 60

SV40 MDKVLNREESLQLMDLLGLERSAWGNIPLMRKAYLKKCKEFHPDKGGDEEKMKKMNTLYK 60

BKV MDKVLNREESMELMDLLGLERAAWGNLPLMRKAYLRKCKEFHPDKGGDEDKMKRMNTLYK 60

JCV MDKVLNREESMELMDLLGLDRSAWGNIPVMRKAYLKKCKELHPDKGGDEDKMKRMNFLYK 60

KIPyV MDKTLSREEAKQLMQLLCLDMSCWGNLPLMRRQYLVKCKEYHPDKGGNEESMKLLNSLYL 60

WUPyV MDKTLSRNEAKELMQLLGLDMTCWGNLPLMRTKYLSKCKEFHPDKGGNEEKMKKLNSLYL 60

HPyV10 MDRVLSRDEVKELMALLSLNTAAWGNIPLMQYKYRQTCLKLHPDKGGDGEKMKRLNELFS 60

STLPyV MDQALSREEAKELMGLLGLPEDSWGNVPLITYRFRQKSKIYHPDKGGNEETMKRMTELYS 60

TSPyV MDKFLSREESLELMDLLQIPRHCYGNFALMKINHKKMSLKYHPDKGGDPEKMSRLNQLWQ 60

HPyV9 MDQTLSLEERNELMDLLQLTRAAWGNLSLMKKAYKTVSKIYHPDKGGNPEKMQRLNELFQ 60

HPyV12 MDSILTFAERQLLISLLKISGDTFGNVPAMARAYKLAAKRLHPDKGGNEAEMKKLNELWN 60

LiPyV MDAVLTTPERRQLCLLLDISPQEYGNIPLMKNAFKKACLKHHPDKGGDPVLMMQLNSLWG 60

MCPyV MDLVLNRKEREALCKLLEIAPNCYGNIPLMKAAFKRSCLKHHPDKGGNPVIMMELNTLWS 60

NJPyV MEKVLEKSDKEMLIELLGIPRYAYGNFPIMKTAYKRASKIYHPDKGGSSEKMMLLNSLWQ 60

MPyV MDRVLSRADKERLLELLKLPRQLWGDFGRMQQAYKQQSLLLHPDKGGSHALMQELNSLWG 60

HaPyV MDRILTKEEKQALISLLDLEPQYWGDYGRMQKCYKKKCLQLHPDKGGNEELMQQLNTLWT 60

*: * : * *: : :*: : . ******. * :. *

HPyV6 KLNATLRDQMSSSPTWCFS--------------S-EVRPPPQYGSPGWEQWWAD------ 99

HPyV7 KLQATLRDQRSGSPMWHYS--------------SDEVRPPPPYGSPAWDQWWQD------ 100

QPyV KFAATMRDQSSGNPIWHFS--------------SEEVRPPPPYGTPEWDKWWHD------ 100

SV40 KMEDGVKYAHQPD--FGG---------------FWDATEIPTYGTDEWEQWWNA------ 97

BKV KMEQDVKVAHQPD--FG----------------TWSSSEVPTYGTEEWESWWSS------ 96

JCV KMEQGVKVAHQPD--FG----------------TWNSSEVPTYGTDEWESWWNT------ 96

KIPyV KLQDSVSSVHDLNEEEDN---------------IWQSSQIPTYGTPDWDEWWSQ------ 99

WUPyV KLQECVSTVHQLNEEEDE---------------VWSSSQIPTYGTPDWDYWWSQ------ 99

HPyV10 KMYTTIEKLRREGEVY------------------FPAKGNPTYGTPEWDQWWEE------ 96

STLPyV RMQNTLQNLRSSNENE------------------HMYPPGGQYGTPAWEQWWEE------ 96

TSPyV KLQEGIYNARQEFPTSFSSQH--DVPT-------QDGRDIPPYGHPSWASWWES------ 105

HPyV9 KLQVTLLEIRSNCGSSSSQGYYSDSPYFTETPFSYCERKNEDPEGGSWGKWWRE------ 114

HPyV12 KFKDGIYNLREYNPHR-----------------------NPSGNPCGSFFWFRRFQSDLF 97

LiPyV KFTTSLTEARAST-----YQ------------------DDPIYGTPQFRAWWYRKHYGFF 97

MCPyV KFQQNIHKLRSDFSMFDEVD------------------EAPIYGTTKFKEW---WRSGGF 99

NJPyV KFQEGLIEVRDSEVFSD------------------------SYGSANFRKRYASWCSSVF 96

MPyV TFKTEVYNLRMNLGGTGFQGS----PP----RTAERGTEESGHSPLHDDYWSFSYGSKYF 112

HaPyV KLKDGLYRVRLLLGPSQDPNA----ST----STSR---PGEFYNPDTGGYWSYSYGSAGY 109

: :

HPyV6 ----------FNRGWD-------------------------------------------- 105

HPyV7 ----------FNKGWD-------------------------------------------- 106

QPyV ----------FNRGWD-------------------------------------------- 106

SV40 ----------FNE----------------------------------------------- 100

BKV ----------FNEKWD-------------------------------------------- 102

JCV ----------FNEKWD-------------------------------------------- 102

KIPyV ----------FNTYWE-------------------------------------------- 105

WUPyV ----------FNSYWE-------------------------------------------- 105

HPyV10 ----------FNRGWD-------------------------------------------- 102

STLPyV ----------FNQPFE-------------------------------------------- 102

TSPyV ----------FNQEWDNLFDTM-------------------------------------- 117

HPyV9 ----------FVNK---------------------------------------------- 118

HPyV12 ADETLSSTSSD-------EEPEPAQRKRGTGANIHESASRTSFSTGSPGKGTRGGGGIPR 150

LiPyV PD---------------------------------GFDPRRS--SSTRNR-RPGGTEEPE 121

MCPyV SFGKAYEYG----------------------PNPHGTNSRSRKPSSNASRGAPSGSSPP- 136

NJPyV TNEKSD--------------------------------SRA---DLHCDESPISSSSDE- 120

MPyV T----REWNDFFRKWDPSYQSPPKTAESSEQPD--LFCYEE------PLL-SPNPSS-PT 158

HaPyV SDQQKKYWEEFFSKWDVNEDLTCQEELSSSEDE---FTPWH------PNP-PPSPVS-IS 158

HPyV6 ------------------------------------------------------------ 105

HPyV7 ------------------------------------------------------------ 106

QPyV ------------------------------------------------------------ 106

SV40 ------------------------------------------------------------ 100

BKV ------------------------------------------------------------ 102

JCV ------------------------------------------------------------ 102

KIPyV ------------------------------------------------------------ 105

WUPyV ------------------------------------------------------------ 105

HPyV10 ------------------------------------------------------------ 102

STLPyV ------------------------------------------------------------ 102

TSPyV ------------------------------------------------------------ 117

HPyV9 ------------------------------------------------------------ 118

HPyV12 DAPPPDSGYGSFPFDSTPPKRG-------R------------------------------ 173

LiPyV YEQPSTSGPNLS----TPR---------------P--KKSR--SNLFGSSGC-------- 150

MCPyV HSQSSSSGYGSFSASQASDSQS-------RGPDIP--PEHH--EEPTSSSGSSSREETTN 185

NJPyV EDETQSSGYNSFPFTSTP------------------------------------------ 138

MPyV DTPAHTAGRRRNPCVAEPDDS--------ISPDPPRTPVSRKRPRPAGATGG-------- 202

HaPyV ------SDSSSSSCDEEYPRNSSRKRKRVHANGSPNTPIQ---PNKRAHTPG-------- 201

HPyV6 -----------------------DEDLYCDEHLSASEEEDN------------------- 123

HPyV7 ------------------------EDLYCTEELSSSDEEEPAAS---------------- 126

QPyV ------------------------EDLYCTEELSASDEEQTA------------------ 124

SV40 ------------------------ENLFCSEEMPSSDDEAT------------------- 117

BKV ------------------------EDLFCHEDMFASDEEAT------------------- 119

JCV ------------------------EDLFCHEEMFASDDENT------------------- 119

KIPyV ------------------------EELRCNESMPSSPKRS-------------------- 121

WUPyV ------------------------EELRCNEEMPKSPGETPTKR---------------- 125

HPyV10 ------------------------EDLSCNESFAPSDEEE-PGPS-----------QSAS 126

STLPyV ------------------------DDLTCNESFNCSDDEG-TSAS-----------QKR- 125

TSPyV ----------------------QDPDLFCHESTIPSDESRSPSPT-----------PGPS 144

HPyV9 ----------------------EYDDLFCSETISSSDDENNPGPS-----------APPP 145

HPyV12 ------------------------------------------------------------ 173

LiPyV -----------------RSRSTAQNPLFCDESLSSSEEEAENASAKSQSDHFSFTSQEES 193

MCPyV SGRESSTPNGTSVPRNSSRTDGTWEDLFCDESLSSPEPPSSSEEPEEPPSSRSSPRQPPS 245

NJPyV ------------------------------------TP-STSTASQEVPPPFSEPQFPES 161

MPyV -GGGGVHANGGSV--------------FG-------HPTGGTSTPAHPP-PYH--SQGGS 237

HaPyV -G-GRTTIRG------------------------------DTDIPRTPA--RE--SQS-- 223

HPyV6 --------------------------VDPG-------------EGNSQDSKYSCTPPKKR 144

HPyV7 ------------------------ASVNPE-------------EGCSQDSKYSATPPKQK 149

QPyV --------------------------EDPE-------------EGCSQNSKYSATPPKQR 145

SV40 --------------------------------------------A----DSQHSTPPKKK 129

BKV --------------------------------------------A----DSQHSTPPKKK 131

JCV -------------------------------------------------GSQHSTPPKKK 130

KIPyV --------------------------APEE-------------EP----SCSQATPPKKK 138

WUPyV ------------------------TREDDE-------------EP----QCSQATPPKKK 144

HPyV10 QT-------A--------N----DTNTPKK-------------RPRESSSNSTCTPPKRP 154

STLPyV --------------------------------------------KFPDYSTQNSTPPKKN 141

TSPyV TQFSEENSRR--------R----RAAPPED-------------SPGCTQSSFSATPPKPK 179

HPyV9 SSAS-----A--------S----EDPDPEE-------------EAGSSQSSFTCTPPKRK 175

HPyV12 ---NGGGSAPSTS-----------G------GVPDDFEG-----DSADQNCSQATPPKSK 208

LiPyV SQAS---APSFTS-----NE-STPASTPKRNRKNQSFGGI---PSPGSRRSFSSTPPKQK 241

MCPyV SSAEEASSSQFTDEEYRSSSFTTPKTPPPF-SRKRKFGGSRSSASSASSASFTSTPPKPK 304

NJPyV SSASGSSSAGRNT--------ETERESPPK-RRRG--TEDLDGSYTDSQTSFASTPPKQK 210

MPyV ESMGGSDSSGFAEGSFRSD--------PRCE------SENESYSQSCSQSSFNATPPK-- 281

HaPyV -TF---------GSYFNST--------EELE------E-----EISQTQQSHHNTTPK-- 252

* **

HPyV6 KPN--PAPNDFPSCLHDYLSHATLGNKCYTCFVSYTTLEKWETLYDKL-----QSAFNAV 197

HPyV7 KPN--PAPQDFPECLSEFLSHATLGNKCYTCFLCYTTYEKSMLLYEKL-----GVEFNAL 202

QPyV KPN--PAPQDFPECINEYLSHATLGNKCYNCFVCYTTMEKSLMLYDKL-----NNEFNAL 198

SV40 R--KVEDPKDFPSELLSFLSHAVFSNRTLACFAIYTTKEKAALLYKKI-----MEKYSVT 182

BKV R--KVEDPKDFPSDLHQFLSQAVFSNRTLACFAVYTTKEKAQILYKKL-----MEKYSVT 184

JCV K--KVEDPKDFPVDLHAFLSQAVFSNRTVASFAVYTTKEKAQILYKKL-----MEKYSVT 183

KIPyV --HAFDASLEFPKELLEFVSHAVFSNKCITCFVVHTTREKGEVLYKKL-----LQKYQCS 191

WUPyV KDNATDASLSFPKELEEFVSQAVFSNRTLTAFVIHTTKEKAETLYKKL-----LSKFKCN 199

HPyV10 RN---FNPVDFPEVLLEFLSNAIFSNKTLNSFVLYTTREKGQFLYEKV-----PLKFKAM 206

STLPyV KP---ADPTDFPAELETFLSHAVFSNKTSNCFCIYTTMEKGNELYTVI-----GPKFKSM 193

TSPyV KSKYDSVPNDFPDMLRPFLSNAVYSNKTLSSFLIYTTNEKAEYLYKKLDKFN------PE 233

HPyV9 KPE-PNTPEDFPMCLYSFLSHAIYSNKTMNCFLIYTTVEKSKQLYRTVEKSKIKVDFKAI 234

HPyV12 KAKMDNGPSDFPCDINIFLSSAVYSNKTVNAFLIFTTVEKCQLLYQKI-----DVKFKID 263

LiPyV RYKEGDDPIDFPNCLSEFLSHATLSNKTYSCFLIFTTAEKGELLYNKV-----SEKYKVE 296

MCPyV KNRETPVPTDFPIDLSDYLSHAVYSNKTVSCFAIYTTSDKAIELYDKI------EKFKVD 358

NJPyV R-KSPDSPSDLPSCLFDFVSHAIFSNKTVNAFILYSTLEKASLLYEKI------DKFKIE 263

MPyV KAREDPAPSDFPSSLTGYLSHAIYSNKTFPAFLVYSTKEKCKQLYDTI------GKFRPE 335

HaPyV KPPPTVSPDDFPTILRGFLSHAIFSNKTQNAFIIYSTKEKCEVLYEQI------DKYNPD 306

.:* : ::* * .*: .* .:* :* ** :

HPyV6 FTGAYKCND---NTGAILYCITPRRHRVSAMLNALSKCCTISFLLIKAVLKSAECYMALQ 254

HPyV7 FIGAYNCVD---GSGALVFFISGSRHRVSAILNACKKHCTVSFIMVKAVLKNAECYKALQ 259

QPyV FIGNYKCND---GSGSIVYMITGSRHRPSAILNASKKYCTVSFSLVKAVLKNAECYKALQ 255

SV40 FISRHNS-----YNHNILFFLTPHRHRVSAINNYAQKLCTFSFLICKGVNKEYLMYSALT 237

BKV FISRHMC-----AGHNIIFFLTPHRHRVSAINNFCQKLCTFSFLICKGVNKEYLLYSALT 239

JCV FISRHGF-----GGHNILFFLTPHRHRVSAINNYCQKLCTFSFLICKGVNKEYLFYSALC 238

KIPyV FISKHAF-----YNTVLIFFLTPHKHRVSAINNFCKGHCTVSFLFCKGVNNPYGLYSRMC 246

WUPyV FASRHSY-----YNTALVFILTPFRHRVSAVNNFCKGYCTISFLFCKGVNNAYGLYSRMT 254

HPyV10 FYSLHEF-----DGDSLLFLLLSGKHRVSAIKNYCSNLCTVSFLLVKGCLKAYECYYALC 261

STLPyV FISCHSY-----NTCCLLFMILAGKHRVSALKNFCSALCSISFVLVKSCLKPYECYYRMC 248

TSPyV FKSRHSF-----QEGSMVFLMTPGKHRVSAIKNLCVTHCTVSFLLCKAVIKQVECYRCMC 288

HPyV9 FLYKDDG-----IEGGLLYFITLGKHRVSAVKHFCVAQCTFSFIHCKAVIKPLELYRALG 289

HPyV12 FKSRHEG---ENKAHGYLYILTVAKHRVSAVKNYCAKQCTISFLHCKAINKPFDCYKALC 320

LiPyV FKSLHNYRGG----TALLFLVLLTRHRVTAIKNFACTFCSVSFLLCKAVIKSPELYSCLI 352

MCPyV FKSRHACEL-----GCILLFITLSKHRVSAIKNFCSTFCTISFLICKGVNKMPEMYNNLC 413

NJPyV FKSLHKLTEGANVGGGLVLVMTIAKHRVSAMKNFCQQFCTVSFLICKVVLKPLECYQCLC 323

MPyV FKCLVHYE-E----GGMLFFLTMTKHRVSAVKNYCSKLCR-SFLMCKAVTKPMECYQVVT 389

HaPyV YKGIFIMKQT----EAFVMFMTPGKHRVAAVKSYCCKFCTVSFLLCKAVTKPLELYNCVA 362

: : : :** :*: * ** * : * :

HPyV6 -GDEFTVIQESRAEGLHSYDFQEGSK-----KEECDWNQVASFASDTDLTDCLALLGYYI 308

HPyV7 -DSKFAVLRESKEGGLHSYDFQEASK-----KDDCDWNFVADFAADMELTDVLLIMGYYM 313

QPyV -GPNFTVIRESREGGLHSYDFQEASK-----KDDCDWNAVAEFALANDLTDPLLIMGYYL 309

SV40 -RDPFSVIEESLPGGLKEHDFNPEEAE---ETKQVSWKLVTEYAMETKCDDVLLLLGMYL 293

BKV -RDPYHTIEESIQGGLKEHDFSPEEPE---ETKQVSWKLITEYAVETKCEDVFLLLGMYL 295

JCV -RQPYAVVEESIQGGLKEHDFNPEEPE---ETKQVSWKLVTQYALETKCEDVFLLMGMYL 294

KIPyV -RQPFNLCEENIPGGLKENEFNPEDLFGEPKEPSLSWNQIANFALEFDIDDVYYLLGSYI 305

WUPyV -RDPFTLCEENIPGGLKENDFKAEDLYGEFKD-QLNWKALSEFALELGIDDVYLLLGLYL 312

HPyV10 -KTPFKLIKQSQEHGLSKTDFCEEEK-----DKVVNWQQICEFAVEVQCEDPLLLMGMLL 315

STLPyV -SSPFSVIKQSRPEGLSQAEFMEQEN----SKPTVNWQQICEFAVQFNCEDPLLLMGIYL 303

TSPyV -SEPFKLLEESKP-GIFEYEFNE-EN----GKPVVNWNLLTDFAVTNRLDDPLLIMAHYL 341

HPyV9 -KPPFKLLEENKP-GVSMFDFQE-EK----E-QAVNWQEICNYAVEAKITDVLLLLGIYL 341

HPyV12 -CDPYKRIESNKD--LFQTDFEN-EN----S-QQVDWTLISTFAECNMIDDPYLIMGHYL 371

LiPyV -KEPFCLLKENKP-GLWDHEFAE-NK----E-PSCNWNLVADFACNYNLTDWVIILAHYL 404

MCPyV -KPPYKLLQENKP--LLNYEFQEKEK----E-ASCNWNLVAEFACEYELDDHFIILAHYL 465

NJPyV -KPPFSQVKANKD-GLFSYDFED-KK----E-ENCNWNKVAEFAVLADIDDPLLILAHYL 375

MPyV -AAPFQLITENKP-GLHQFEFTDEPE----EQKAVDWIMVADFALENNLDDPLLIMGYYL 443

HaPyV KCDDFQILKENKP-GLYHFEFCDEKK----EVKQIDWNFLTSFAVENELDDPLVIMGHYL 417

: . : :* .* : :* * ::. :

HPyV6 EFANDPASCMKCKKGV--KV--HKHHEVHFHNAQLFKAAKNQKSIAQQACDRVAAQRRVL 364

HPyV7 EFATEPSLCPKCLKSV--KA--HQHHEKHWANAKLFKTAKNQKGIAQQAADRVLAARRVL 369

QPyV EFAAEPSLCQKCKKGV--KA--HKCHELQWSNAKLFKSAKNQRGIATQAADRVLSARRVM 365

SV40 EFQYSFEMCLKCIKKE--QPSHYKYHEKHYANAAIFADSKNQKTICQQAVDTVLAKKRVD 351

BKV EFQYNVEECKKCQKKD--QPYHFKYHEKHFANAIIFAESKNQKSICQQAVDTVLAKKRVD 353

JCV DFQENPQQCKKCEKKD--QPNHFNHHEKHYYNAQIFADSKNQKSICQQAVDTVAAKQRVD 352

KIPyV RFATKPEECEKCSKND--DATHKRVHVQNHENAVLLQESKSQKNACTQAIDRVIAERRYN 363

WUPyV QLSIKVEECEKCNSNE--DATHNRLHMEHQKNALLFSDSKSQKNVCQQAIDVVIAKRRVD 370

HPyV10 DFAKDVEGCSKCEQKK--LKHHYKFHEAQNINSKLFKDCKNQKTICQQATDWVTAQRRLL 373

STLPyV DFSESPDNCEKCRT-E--LKHHNQFHEKEHNNAKLFRDSKTQKTLCQQACDWVCAKRRVL 360

TSPyV DFAEEPSICSKCTKKA--LKAHYNYHSLHHKNAKLFKECKTQKTACQQAADVVMAKQRLK 399

HPyV9 DFAVEPGTCSKCEKKS--HKFHYNYHSKHHANACLFLESKSQKNICQQAVDQVLAAKRLK 399

HPyV12 DFAS-PLPCNKCQMKV--LKVHYQFHEAHHNNAILFKNSKAQKTICQQAADVVIAKRRLH 428

LiPyV DFANDPALCDKCSKLP---LKPHEAHRKNYENAKFFLKCKSQKTICQQAADVVIAKNRLK 461

MCPyV DFAK-PFPCQKCENRS--RLKPHKAHEAHHSNAKLFYESKSQKTICQQAADTVLAKRRLE 522

NJPyV DFAQ-PFPCLKCEHQK---TKAHDYHKAHHENAVLFEACKSQRSICNQASDIVLAKRRLL 431

MPyV DFAKEVPSCIKCSKEETRLQIHWKNHRKHAENADLFLNCKAQKTICQQAAAS-LASRRLK 502

HaPyV EFSQCESSCKKCAEALPRMKVHWANHSQHLENAELFLHCKQQKSICQQAADNVLARRRLK 477

: * ** * . *: :: .* *: . ** : .*

HPyV6 MLESTRQDLVLQAFKKQFTILAEQYA--GGVEITQLLGAVAWLDCLQPSFTTKLKEILSI 422

HPyV7 MMESTRKDLMVMSFKKQFKVLAEQFG--GGVEITQLIGAVAWLDCLLPQFTIKIKEMLSY 427

QPyV MIESTRVDLMVMAFKKQFQVLNDQFA--GGVEITQLLGAVAWLDCLMPSFTTKLKEMLTL 423

SV40 SLQLTREQMLTNRFNDLLDRMDIMFGSTGSADIEEWMAGVAWLHCLLPKMDSVVYDFLKC 411

BKV TLHMTREEMLTERFNHILDKMDLIFGAHGNAVLEQYMAGVAWLHCLLPKMDSVIFDFLHC 413

JCV SIHMTREEMLVERFNFLLDKMDLIFGAHGNAVLEQYMAGVAWIHCLLPQMDTVIYDFLKC 412

KIPyV CLTLTRKKLLTKRFKKLFNEMDKIVV--GERKILLYMASIAWYTGLNKKIDELVVRFLKL 421

WUPyV SLNMSREDLLARRFEKILDKMDKTIK--GEQDVLLYMAGVAWYLGLNGKIDELVYRYLKV 428

HPyV10 ILESTREHLLVLRFKHMFEKMEDI-C--GEVEICQYMAGVAWLSLLMPHFDEIILFIIKA 430

STLPyV ILESTREDLLVIRFKQVLKEMQDI-A--GEVEILRYMAGVAWLSLLFNHFDDIVLEIIRT 417

TSPyV LIESTRKELLEERFKLMFEKLTDE-F--GQIKILQYMAGVAWYSCLFENIDEVVTKILKL 456

HPyV9 LVECTRMELLEDRFIQLFDEMEDFLH--GEIEILRWMSGVAWYTILLDNSWDVFQKILQL 457

HPyV12 LIESTREELLAERFKLFLNKYKEL----DKMRVLEHMAGVMWYSVMFENIDRIVIQILKL 484

LiPyV MLEQTREEMLREKILCKLKELQEM----KLETLYIFLAGVAWYKVMFGNFEWKVFKVLNL 517

MCPyV MLEMTRTEMLCKKFKKHLERLRDL----DTIDLLYYMGGVAWYCCLFEEFEKKLQKIIQL 578

NJPyV LTESTREELLAMCFQKQLKALQAL----DTLEIYDHMAGVAWYANLFENFDDILFQILKL 487

MPyV LVECTRSQLLKERLQQSLLRLKEL----GSSDALLYLAGVAWYQCLLEDFPQTLFKMLKL 558

HaPyV VLESTRQELLAERLNKLLDQLKDL----SPVDKHLYLAGVAWYQCMFPDFEMMLLDILKL 533

:* .:: : : :..: * : . :

HPyV6 LTENIPKKRNVLFKGPINSGKTTLAAAILDLVGGVSLNVNCTPDKINFELGCAIDKFMCV 482

HPyV7 LVENTPKRRNLLFKGPINSGKTTLAAAILDLLGGVALNVNCSSDKINFELGCAIDKYMVV 487

QPyV LVQNYAKKRNLLFKGPINSGKTTVAAGIMDLLGGVALNVNCSSDKINFELGCAIDKMLVV 483

SV40 MVYNIPKKRYWLFKGPIDSGKTTLAAALLELCGGKALNVNLPLDRLNFELGVAIDQFLVV 471

BKV IVFNVPKRRYWLFKGPIDSGKTTLAAGLLDLCGGKALNVNLPMERLTFELGVAIDQYMVV 473

JCV IVLNIPKKRYWLFKGPIDSGKTTLAAALLDLCGGKSLNVNMPLERLNFELGVGIDQFMVV 472

KIPyV IVDNKPKHRYWLFKGPINSGKTTLATALLNLCGGKALNINIPSEKLPFELGVALDQYMVV 481

WUPyV IVENVPKKRYWVFKGPINSGKTTVAAALLDLCGGKALNINIPADRLNFELGVAIDQFTVV 488

HPyV10 MTENVPKRRYVLFKGPINSGKTTVAAAILDLLGGKTLNVNCPPDKLAFEIGCAIDEYMVV 490

STLPyV MVVNTPKRRYFLFKGPINSGKTTVAAAILDLLGGRTLNINCPPEKVNFELGCAIDEFMVV 477

TSPyV IVENVPKKRNCLFRGPINSGKTTFAAALMNFLGGKTLNVNCPADKLPFELGCAIDQFVVI 516

HPyV9 VTTSQPKKRNILFKGPINSGKTTLASAFMHFFDGKALNINCPAEKLSFELGCAIDQFCVL 517

HPyV12 MTENIPKKRNVLFKGPINSGKTSFAAAMLDLISGKTLNINCPADKLPFELGCALDQFAVV 544

LiPyV LTDNIPKKRNVLFKGPINSGKTSLAAAFLDLLEGKALNINCPQDRLNFELGCAQDLFMVC 577

MCPyV LTENIPKYRNIWFKGPINSGKTSFAAALIDLLEGKALNINCPSDKLPFELGCALDKFMVV 638

NJPyV LTQNIPKQRNILFRGPVNSGKTTFAAALVDLLGGRSLNVNCPADKLNFELGCAIDRFFVV 547

MPyV LTENVPKRRNILFRGPVNSGKTGLAAALISLLGGKSLNINCPADKLAFELGVAQDQFVVC 618

HaPyV FTENVPKKRNVLFRGPVNSGKTSLAAAIMNLVGGVALNVNCPADKLNFELGVAIDKFAVV 593

.. . * * *:**::**** .*:.:: : * :**:* ::: **:* . *

HPyV6 IEDVKGTPMA-NTNLTQGCGMTNLDNLRDYLDGCVPVNMERKHLNKVSQLFPPSVITCND 541

HPyV7 IEDVKGTPLP-NTDLPSGVGMANLDNMRDYLDGCVPVNLERKHINKTSQLFPPCIITCNE 546

QPyV FEDVKGQPLP-NTDLPAGVGMANLDNLRDHLDGCVPVNLERKHTNKVSQLFPPCIITCND 542

SV40 FEDVKGTGGE-SRDLPSGQGINNLDNLRDYLDGSVKVNLEKKHLNKRTQIFPPGIVTMNE 530

BKV FEDVKGTGAE-SKDLPSGHGINNLDSLRDYLDGSVKVNLEKKHLNKRTQIFPPGLVTMNE 532

JCV FEDVKGTGAE-SRDLPSGHGISNLDCLRDYLDGSVKVNLERKHQNKRTQVFPPGIVTMNE 531

KIPyV FEDVKGQIGI-EKQLPSGNGVNNLDNLRDYLDGCVEVNLEKKHVNKRSQIFPPGIVTMNE 540

WUPyV FEDVKGQVGD-NKLLPSGNGMSNLDNLRDYLDGSVKVNLEKKHLNKRSQIFPPGIVTMNE 547

HPyV10 FEDVKGQNEGSNSSLTPGMGMSNLDNLRDHLDGCVKVNLEKKHVNKKSQIFPPGIITMND 550

STLPyV FEDVKGQTEGK-TNLTSGMGMNNLDSLRDHLDGCVKVNLEKKHLNKRSQIFPPGIITMNE 536

TSPyV FEDVKGQIAL-NKKLQPGQGVSNLDNLRDHLDGSVKVNLERKHVNKRSQIFPPCLVTMNE 575

HPyV9 LDDVKGQITL-NKHLQPGQGVNNLDNLRDHLDGTIKVNLEKKHVNKRSQIFPPVIMTMNE 576

HPyV12 FEDVKGQVGN-DKTLQCGQGVNNLDNIRDHLDGSVTVNLEKKHVNKKTQIFPPCIVTMND 603

LiPyV FEDVKGSRGQ-NKDLPSGQGMHNLDNLRDHMDGSVNVNLEKKHQNKRSQIFPPSITTCNE 636

MCPyV FEDVKGQNSL-NKDLQPGQGINNLDNLRDHLDGAVAVSLEKKHVNKKHQIFPPCIVTAND 697

NJPyV FEDVKGQNML-NKKLQPGQGISNLDNMRDYLDGAVPVNLEKKHMNKRSQVFPPCVMTMNE 606

MPyV FEDVKGQIAL-NKQLQPGMGVANLDNLRTTWNGSVKVNLEKKHSNKRSQLFPPCVCTMNE 677

HaPyV FEDVKGQTGD-KRHLQSGLGINNLDNLRDYLDGSVKVNLEKKHVNKRSQIFPPCIVTANE 652

::**** * * *: *** :* :* : *.:*:** ** *:*** : * *:

HPyV6 YIIPCTVKARIARGYYFLHKPCLQKCLKD-CVLMSKRLLQKGTTLLAALIWWEPVEDFME 600

HPyV7 YAIPTTVKARVAKGYYFLHKPGLKKSLDANPILMKKRLLQKGCTLLAALIWWEPVSDFVE 606

QPyV YAIPRTVKARVAKGYYFIHKPNLKKCLDVNPILMQKRLLQKGVTLLAALIWWEPVSEFVE 602

SV40 YSVPKTLQARFVKQIDFRPKDYLKHCLERSEFLLEKRIIQSGIALLLMLIWYRPVAEFAQ 590

BKV YPVPKTLQARFVRQIDFRPKIYLRKSLQNSEFLLEKRILQSGMTLLLLLIWFRPVADFAT 592

JCV YSVPRTLQARFVRQIDFRPKAYLRKSLSCSEYLLEKRILQSGMTLLLLLIWFRPVADFAA 591

KIPyV YCIPETVAVRFEKTVMFTIKRNLRESLEKTPQLLSQRILHSGIAMLLLLIWYRPVSDFDE 600

WUPyV YLVPATLAPRFHKTVLFTPKRHLKESLDKTPELMVKRVLQSGMCILIMLIWCRPVSDFHP 607

HPyV10 YFIPPTLQARMIKTINFRPKLFLRNSLEKNSELLRKRIVQSGVTLLLLLCWWQPVIAFHP 610

STLPyV YNVPLTILARMVKVINFRPKHYLKKSLEVNNELLHRRIVQSGKTLLMLLMWWQPVKVFHS 596

TSPyV YLLPETIFTRFAYVLNFTPKHNLRSCLQVSDYLLTERILQDGVTIALLLVWYCPITMFSE 635

HPyV9 YLLPPTVGVRFALHIHFHCKTYLKQSLEKSD-LIEKRILNSGYTILLLLLWYNPVDSFTP 635

HPyV12 YKLPPTVKARFAYMVIFTHMKCLQTSLEKNDDIVKHRITHSGLTMFMILMWYCSSSAFIP 663

LiPyV YIIPDTVMCRFAITITFAHKENLRTSLRKNIDMQKLRVLQRGCTLLLGLMWLLPKEKFDD 696

MCPyV YFIPKTLIARFSYTLHFSPKANLRDSLDQNMEIRKRRILQSGTTLLLCLIWCLPDTTFKP 757

NJPyV YFMPQTLFVRFSLKLDFVSRPNLQSAVDKTPGLVANRILQKGLTLFLLLIWYTPVKKFAV 666

MPyV YLLPQTVWARFHMVLDFTCKPHLAQSLEKCEFLQRERIIQSGDTLALLLIWNFTSDVFDP 737

HaPyV YFFPQTLYARFHKVYNFEVKDFLAKSLEENSYMGRHRVCQSPLTMLIALLWNVPTENFDK 712

* .* *: *. * * .: : *: : : * * *

HPyV6 ELQEDVVNWKQTFERWVSFGMYQTMKENILAGIDPFTNVLVDESFVQPQENDET-ND--- 656

HPyV7 EIQEEVVNWKQTFEQWVSYGMFQTMKENILSGKDPFEGVLINDPTE---ENTRE-TQ--- 659

QPyV EIQEDVVNWKQTFERWVTYGMYQDMKQNILAGKDPFYGVIMSDIN----EIVEE-TQ--- 654

SV40 SIQSRIVEWKERLDKEFSLSVYQKMKFNVAMGIGVLDWLRNSDDDDEDSQENADKNED-- 648

BKV DIQSRIVEWKERLDSEISMYTFSRMKYNICMGKCILDITREEDSETEDSG---------- 642

JCV AIHERIVQWKERLDLEISMYTFSTMKANVGMGRPILDFPREEDSEAEDSG---------- 641

KIPyV EIQSNVVYWKEVLDNYIGLTEFATMQMNVTNGKNILEKWFE------------------- 641

WUPyV CIQAKVVYWKELLDKYIGLTEFADMQMNVTNGCNILEKHNA------------------- 648

HPyV10 EIHDNVRYWKETIEKYVPFGMYHDIRRNIESGEDPLKDILICVDADEDT------QQ--- 661

STLPyV SIHEDVKLWKDTLTKYVSIGMFHDIQKNIQNGEDPLKNILICEDTENNE------TQ--- 647

TSPyV SIKEDVKYWKDILCKYMGHTNFATLLLNVEEGKDPLDSVVIEVEDEEEEEFSE--TN--- 690

HPyV9 KVQEYVVKWKEILERHVSITQFGNIQQNILDGKDPLHGIVIEEQA--------------- 680

HPyV12 SLRETIEIEKKLLESICTTEIACLMKDNIKAGRDPLHDIVTEADE--------------- 708

LiPyV EIRPEVERWRDAFRGDIPQAHFEKMIQNVECGLDPLEDLFVEAPADAPPAAAPEDSEAEP 756

MCPyV CLQEEIKNWKQILQSEISYGKFCQMIENVEAGQDPLLNILIEEEGPEETE---------- 807

NJPyV SLQEEIANWKCIIEKTVSHSDFCKMLENIEVGESPLTDLIDEGDN--------------- 711

MPyV DIQGLVKEVRDQFASECSYSLFCDILCNVQEGDDPLKDICDIAEYTVY------------ 785

HaPyV SLKEKVETEKKVLSDMCNFTTFAEMCLNIQRGADPLEAL--------------------- 751

:: : : : : *: * :

HPyV6 -----------------------------STQESGIGSMHSM------------------ 669

HPyV7 -----------------------------ESTESGIGSMNN------------------- 671

QPyV -----------------------------ESTESGVGSMET------------------- 666

SV40 -------------------------GGEKNMEDSGHETGIDSQSQGSF------QAPQSS 677

BKV -----------------------------------HGSSTESQSQCSSQVSDTSAPAEDS 667

JCV -----------------------------------HGSSTESQSQCFSQVSEASGAD--- 663

KIPyV ------------------------------------------------------------ 641

WUPyV ------------------------------------------------------------ 648

HPyV10 --------------------------------DSG----INSQ----------------- 668

STLPyV --------------------------------DSA----FCTQDSDNE------------ 659

TSPyV --------------------------------DSG----FQTQ----------------- 697

HPyV9 ------------------------------------------------------------ 680

HPyV12 ------------------------------------------------------------ 708

LiPyV PVASKVPHQHENTMEEPERQQKARPEKDLESQDSG----LFTQDSGQT------------ 800

MCPyV -----------------------------ETQDSG----TFSQ----------------- 817

NJPyV ------------------------------------------------------------ 711

MPyV ------------------------------------------------------------ 785

HaPyV ------------------------------------------------------------ 751

HPyV6 ------------------------------- 669

HPyV7 ------------------------------- 671

QPyV ------------------------------- 666

SV40 QSVHDHNQPYHICRGFTCFKKPPTPPPEPET 708

BKV QRSDPHSQELHLCKGFQCFKRPKTPPPK--- 695

JCV ---TQENCTFHICKGFQCFKKPKTPPPK--- 688

KIPyV ------------------------------- 641

WUPyV ------------------------------- 648

HPyV10 ------------------------------- 668

STLPyV ------------------------------- 659

TSPyV ------------------------------- 697

HPyV9 ------------------------------- 680

HPyV12 ------------------------------- 708

LiPyV ------------------------------- 800

MCPyV ------------------------------- 817

NJPyV ------------------------------- 711

MPyV ------------------------------- 785

HaPyV ------------------------------- 751
