# Supplementary material for: Functional Domains of the Early Proteins and Experimental and Epidemiological Studies Suggest a Role for the Novel Human Polyomaviruses in Cancer
Source: Front Microbiol. 2022 Feb 18;13:834368. doi: 10.3389/fmicb.2022.834368 (PMC8894888; doi:10.3389/fmicb.2022.834368)
Supplement: Supplementary file 2 [file Table_2.DOCX]

Alignment sT

**>MPyV sT (J02288): 196 aa**

MDRVLSRADKERLLELLKLPRQLWGDFGRMQQAYKQQSLLLHPDKGGSHALMQELNSLWGTFKTEVYNLRMNLGGTGFQVRRLHADGWNLSTKDTFGDRYYQRFCRMPLTCLVNVKYSSCSCILCLLRKQHRELKDKCDARCLVLGECFCLECYMQWFGTPTRDVLNLYADFIASMPIDWLDLDVHSVYNPTGLSP

**>sT HaPyV (NC_001663): 194 aa**

MDRILTKEEKQALISLLDLEPQYWGDYGRMQKCYKKKCLQLHPDKGGNEELMQQLNTLWTKLKDGLYRVRLLLGPSQVRRLGKDQWNLSLQQTFSGTYFRRLCRLPITCLRNKGISTCNCILCLLRKQHFLLKKSWRVPCLVLGECYCIDCFALWFGLPVTNMLVPLYAQFLAPIPVDWLDLNVHEVYNPASGP

**>SV40 st-ag (NC_001669): 174 aa**

MDKVLNREESLQLMDLLGLERSAWGNIPLMRKAYLKKCKEFHPDKGGDEEKMKKMNTLYKKMEDGVKYAHQPDFGGFWDATEVFASSLNPGVDAMYCKQWPECAKKMSANCICLLCLLRMKHENRKLYRKDPLVWVDCYCFDCFRMWFGLDLCEGTLLLWCDIIGQTTYRDLKL

**>BKPyV st-ag (NC_001538): 172 aa**

MDKVLNREESMELMDLLGLERAAWGNLPLMRKAYLRKCKEFHPDKGGDEDKMKRMNTLYKKMEQDVKVAHQPDFGTWSSSEVCADFPLCPDTLYCKEWPICSKKPSVHCPCMLCQLRLRHLNRKFLRKEPLVWIDCYCIDCFTQWFGLDLTEETLQWWVQIIGETPFRDLKL

**>JCPyV st-ag (NC_001699): 172 aa**

MDKVLNREESMELMDLLGLDRSAWGNIPVMRKAYLKKCKELHPDKGGDEDKMKRMNFLYKKMEQGVKVAHQPDFGTWNSSEVGCDFPPNSDTLYCKEWPNCATNPSVHCPCLMCMLKLRHRNRKFLRSSPLVWIDCYCFDCFRQWFGCDLTQEALHCWEKVLGDTPYRDLKL

**>KIPyV (NC_009238): 191 aa**

MDKTLSREEAKQLMQLLCLDMSCWGNLPLMRRQYLVKCKEYHPDKGGNEESMKLLNSLYLKLQDSVSSVHDLNEEEDNIWQSSQVYCKDLCCNKFRLVGAIYGDYYEAYIMKQWDVCIHGYNHECQCIHCILSKYHKEKYKIYRKPPVWIECYCYKCYREWFFFPISMQTFFFWKVIIFNTEIRAVQPLLR

**>WUPyV (NC_009539): 194 aa**

MDKTLSRNEAKELMQLLGLDMTCWGNLPLMRTKYLSKCKEFHPDKGGNEEKMKKLNSLYLKLQECVSTVHQLNEEEDEVWSSSQVECTELCCNFPPRKYRLVGEVYGDVFEEYILKDWDICLKGFYYLCNCFYCFLDKRHKQKYKIFRKPPMWIECYCYRCYREWFGFEISAETFFYWKKIIFLTTMQGVGLTR

**>MCPyV (NC_010277): 196 aa**

MDLVLNRKEREALCKLLEIAPNCYGNIPLMKAAFKRSCLKHHPDKGGNPVIMMELNTLWSKFQQNIHKLRSDFSMFDEVSTKFPWEEYGTLKDYMQSGYNARFCRGPGCMLKQLRDSKCACISCKLSRQHCSLKTLKQKNCLTWGECFCYQCFILWFGFPPTWESFDWWQKTLEETDYCLLHLHLF

**>HPyV6 st-ag (NC_014406): 190 aa**

MDRLLAREEVRELMDLIGLSMACWGNLPLMQQKIRLACKKYHPDKGGDPEKMQRLNVLKEKLNATLRDQMSSSPTWCFSSEVSDDWGIPLTVGEFLGPEFHKKKVWDFRLCVQQGISSCKCLHCLLKKEHKKQVEINLGKPTIWGKCWCYKCYCLWFGLPVEADSFMWWTHIIYQSPLDWLGITEKLIWW

**>HPyV7 st-ag (NC_014407): 193 aa**

MDKLLGRDEVKELMELIGLNMACWGNLPLIQHKVRLASKKYHPDKGGDPQKMQRLNVLKDKLQATLRDQRSGSPMWHYSSDEVSFWDIELTVGEFLGPEFNRKKVWNYNLCVVQGLRACCCIHCILKRKHKKKAKEYAKDHRGPLLWGKCWCFDCYLDWFGVERSEESFMWWSHIIFQTPMDVLNLWGQLNLL

**>TSPyV st-ag (NC_014361): 198 aa**

MDKFLSREESLELMDLLQIPRHCYGNFALMKINHKKMSLKYHPDKGGDPEKMSRLNQLWQKLQEGIYNARQEFPTSFSSQVGSWYWEANLISLKEYFGKKKYDENVIKHWPQCAEKALKECKCLTCKIGLQHHVYKQMHQKKCVVWGECFCYKCYCAWFGEDLYCLDSLWAWSCIVGEVDFHLVNLYLRVNQGFNWGK

**>HPyV9 (NC_015150): 189 aa**

MDQTLSLEERNELMDLLQLTRAAWGNLSLMKKAYKTVSKIYHPDKGGNPEKMQRLNELFQKLQVTLLEIRSNCGSSSSQVAWYFWDENFRTLGAFLGEKFNQRIIGGYPDCITYNKPSCCCIVCLLKQQHKSTKINKKKPCLVWGECFCYKCYLLWFGFPEDFTSFNYWTLLMRNMDLSLLRLWTELGF

**>HPyV10 (JX262162): 206 aa**

MDRVLSRDEVKELMALLSLNTAAWGNIPLMQYKYRQTCLKLHPDKGGDGEKMKRLNELFSKMYTTIEKLRREGEVYFPAKVGYFIDDVVTLGDVLGPSFEEKIIYIWPLCASDLLRHKCGCVCCLLKKQHRNDKLAKQKQCLVWGECFCYKCFLLWFGQEFGYTSFFWWKHIMHNTEFDLLCLLGELILWVSYFSFILGKSHLWDS

**>STLPyV (NC_020106): 195 aa**

MDQALSRQEAKELMGLLGLPEDSWGNVPLITYRFRQKSKIYHPDKGGNEETMKRMTELYSRMQNTLQNLRSSNENENMYPPVRMLLLTDTFTLGELLGPQFESKVIFIWPTCAKCRYRTFCQCVCCILKRQHDEIKKVRNKPCVTWGECYCFDCFLLWFGCDLTKASLHAWKHVMYNLDLDLLMFKQLNLVSFSF

**>HPyV12 (NC_020890): 182 aa**

MDSILTFAERQLLISLLKISGDTFGNVPAMARAYKLAAKRLHPDKGGNEAEMKKLNELWNKFKDGIYNLREVKPSLHPVVTCTVLGARNIFNLITNSSQCMRNLLRYCRCFCCILFQQHRQLKITYRRRCNVWGQCYCFLCYYTWFGVNCSIGAFTEWLILLKHLDWRLLKISSAELDVLGK

**>NJPyV (NC_024118): 183 aa**

MEKVLEKSDKEMLIELLGIPRYAYGNFPIMKTAYKRASKIYHPDKGGSSEKMMLLNSLWQKFQEGLIEVRDSEVCQVSFSDCYDSSLLKCCSPKVFHELFLRSPQCLLKGPTSCSCITSCLYNQHRQIKLCGKKRCLTWGNCFCFSCFILWFGLRETWKTFEIWKHVIAQMPAALLQLSPSLF

**>LiPyV (NC_034253: 179 aa**

MDAVLTTPERRQLCLLLDISPQEYGNIPLMKNAFKKACLKHHPDKGGDPVLMMQLNSLWGKFTTSLTEARASTYQASTLFWEIDNPLKNLLGPVIKRPFLKSPHCINSKFYNCRCIVCSLSDQHSSLKILQKKKCLIWGECYCYYCFVTWFGLPGNSATFEDYKNLILEMDVDLLNLHC

**>QPyV (BK010702): 193 aa**

MDRLLSRDEVNELMQLIGLSMSNWGNLPLIQHKVREACKKHHPDKGGDPEKMQRLNVLKDKFAATMRDQSSGNPIWHFSSEEVSFWDLQLTVGEFLGVEFNRKKLWNFELCVLQGLRACCCLHCLLRRKHKKLAKQMAKDQKGPLVWGHCWCFQCYLQWFGEDKNKESFEWWTQIIYGTQMDVINIWGQINLL

CLUSTAL O(1.2.4) multiple sequence alignment

SV40 MDKVLNREESLQLMDLLGLERSAWGNIPLMRKAYLKKCKEFHPDKGGDEEKMKKMNTLYK 60

BKPYV MDKVLNREESMELMDLLGLERAAWGNLPLMRKAYLRKCKEFHPDKGGDEDKMKRMNTLYK 60

JCPYV MDKVLNREESMELMDLLGLDRSAWGNIPVMRKAYLKKCKELHPDKGGDEDKMKRMNFLYK 60

KIPyV MDKTLSREEAKQLMQLLCLDMSCWGNLPLMRRQYLVKCKEYHPDKGGNEESMKLLNSLYL 60

WUPyV MDKTLSRNEAKELMQLLGLDMTCWGNLPLMRTKYLSKCKEFHPDKGGNEEKMKKLNSLYL 60

HPyV12 MDSILTFAERQLLISLLKISGDTFGNVPAMARAYKLAAKRLHPDKGGNEAEMKKLNELWN 60

MPyV MDRVLSRADKERLLELLKLPRQLWGDFGRMQQAYKQQSLLLHPDKGGSHALMQELNSLWG 60

sT MDRILTKEEKQALISLLDLEPQYWGDYGRMQKCYKKKCLQLHPDKGGNEELMQQLNTLWT 60

NJPyV MEKVLEKSDKEMLIELLGIPRYAYGNFPIMKTAYKRASKIYHPDKGGSSEKMMLLNSLWQ 60

MCPyV MDLVLNRKEREALCKLLEIAPNCYGNIPLMKAAFKRSCLKHHPDKGGNPVIMMELNTLWS 60

LiPyV MDAVLTTPERRQLCLLLDISPQEYGNIPLMKNAFKKACLKHHPDKGGDPVLMMQLNSLWG 60

TSPyV MDKFLSREESLELMDLLQIPRHCYGNFALMKINHKKMSLKYHPDKGGDPEKMSRLNQLWQ 60

HPyV6 MDRLLAREEVRELMDLIGLSMACWGNLPLMQQKIRLACKKYHPDKGGDPEKMQRLNVLKE 60

HPyV7 MDKLLGRDEVKELMELIGLNMACWGNLPLIQHKVRLASKKYHPDKGGDPQKMQRLNVLKD 60

QPyV MDRLLSRDEVNELMQLIGLSMSNWGNLPLIQHKVREACKKHHPDKGGDPEKMQRLNVLKD 60

HPyV9 MDQTLSLEERNELMDLLQLTRAAWGNLSLMKKAYKTVSKIYHPDKGGNPEKMQRLNELFQ 60

HPyV10 MDRVLSRDEVKELMALLSLNTAAWGNIPLMQYKYRQTCLKLHPDKGGDGEKMKRLNELFS 60

STLPyV MDQALSRQEAKELMGLLGLPEDSWGNVPLITYRFRQKSKIYHPDKGGNEETMKRMTELYS 60

*: * : * *: : :*: : . ******. * :. *

SV40 KMEDGVKYAHQPD--FGGFWDATEVFASS----LNPGV-------------DAMYCKQWP 101

BKPYV KMEQDVKVAHQPD--FGT-WSSSEV-CAD----FPLCP-------------DTLYCKEWP 99

JCPYV KMEQGVKVAHQPD--FGT-WNSSEV-GCD----FPPNS-------------DTLYCKEWP 99

KIPyV KLQDSVSSVHDLNEEEDNIWQSSQVYCKDLCCN----KFRLVGAIYGD-YYEAYIMKQWD 115

WUPyV KLQECVSTVHQLNEEEDEVWSSSQVECTELCCNFPPRKYRLVGEVYGD-VFEEYILKDWD 119

HPyV12 KFKDGIYNLREVKP----SLHP-----------------VVTCTVLGA-RNIFNLITNSS 98

MPyV TFKTEVYNLRMNLG----GTGF-QVRRL-----HADGWNLSTKDTFGD-RYYQRFCRMPL 109

sT KLKDGLYRVRLLLG----PS---QVRRL-----GKDQWNLSLQQTFSG-TYFRRLCRLPI 107

NJPyV KFQEGLIEVRDSEV----CQV-SFSDC--------Y-DSSLLKCCSPK-VFHELFLR-SP 104

MCPyV KFQQNIHKLRSDFS----MFD-EVSTKF-----PWE-EYGTLKDYMQS-GYNARFCR-GP 107

LiPyV KFTTSLTEARASTY---------QASTL-----FWE-IDNPLKNLLGP-VIKRPFLK-SP 103

TSPyV KLQEGIYNARQEFP----TSFSSQVGSW-----YWEANLISLKEYFGKKKYDENVIKHWP 111

HPyV6 KLNATLRDQMSSSP----TWCFSSEVSD-----DWG-IPLTVGEFLGP-EFHKKKVWDFR 109

HPyV7 KLQATLRDQRSGSP----MWHYSSDEVS-----FWD-IELTVGEFLGP-EFNRKKVWNYN 109

QPyV KFAATMRDQSSGNP----IWHFSSEEVS-----FWD-LQLTVGEFLGV-EFNRKKLWNFE 109

HPyV9 KLQVTLLEIRSNCG----SSS-SQVAWY-----FWDENFRTLGAFLGE-KFNQRIIGGYP 109

HPyV10 KMYTTIEKLRRE------GEVYFPAKVG-----YFIDDVVTLGDVLGP-SFEEKIIYIWP 108

STLPyV RMQNTLQNLRSSNE----NENMYPPVRM-----LLLTDTFTLGELLGP-QFESKVIFIWP 110

: :

SV40 ECAKKMS--ANCICLLCLLRMKHENRKL----YRKDPLVWVDCYCFDCFRMWFGLDLC-E 154

BKPYV ICSKKPS--VHCPCMLCQLRLRHLNRKF----LRKEPLVWIDCYCIDCFTQWFGLDLT-E 152

JCPYV NCATNPS--VHCPCLMCMLKLRHRNRKF----LRSSPLVWIDCYCFDCFRQWFGCDLT-Q 152

KIPyV VCIHGYN--HECQCIHCILSKYHKEKYK----IYRKPPVWIECYCYKCYREWFFFPIS-M 168

WUPyV ICLKGFY--YLCNCFYCFLDKRHKQKYK----IFRKPPMWIECYCYRCYREWFGFEIS-A 172

HPyV12 QCMRNLL--RYCRCFCCILFQQHRQLKI---TYRRRCNVWGQCYCFLCYYTWFGVNCS-I 152

MPyV TCLVNV-KYSSCSCILCLLRKQHRELKD---KCDARCLVLGECFCLECYMQWFGTPTR-D 164

sT TCLRNK-GISTCNCILCLLRKQHFLLKK---SWRVPCLVLGECYCIDCFALWFGLPVT-N 162

NJPyV QCLLKGP--TSCSCITSCLYNQHRQIKL---CGKKRCLTWGNCFCFSCFILWFGLRET-W 158

MCPyV GCMLKQLRDSKCACISCKLSRQHCSLKT---LKQKNCLTWGECFCYQCFILWFGFPPT-W 163

LiPyV HCINSKF--YNCRCIVCSLSDQHSSLKI---LQKKKCLIWGECYCYYCFVTWFGLPGN-S 157

TSPyV QCAEKAL--KECKCLTCKIGLQHHVYKQ---MHQKKCVVWGECFCYKCYCAWFGEDLYCL 166

HPyV6 LCVQQGI--SSCKCLHCLLKKEHKKQV---EINLGKPTIWGKCWCYKCYCLWFGLPVE-A 163

HPyV7 LCVVQGL--RACCCIHCILKRKHKKKAKEYAKDHRGPLLWGKCWCFDCYLDWFGVERS-E 166

QPyV LCVLQGL--RACCCLHCLLRRKHKKLAKQMAKDQKGPLVWGHCWCFQCYLQWFGEDKN-K 166

HPyV9 DCITYN-K-PSCCCIVCLLKQQHKSTKI---NKKKPCLVWGECFCYKCYLLWFGFPED-F 163

HPyV10 LCASDLLR-HKCGCVCCLLKKQHRNDKL---AKQKQCLVWGECFCYKCFLLWFGQEFG-Y 163

STLPyV TCAKCRYR-TFCQCVCCILKRQHDEIKK---VRNKPCVTWGECYCFDCFLLWFGCDLT-K 165

* * *. . : * .*:* *: **

SV40 GTLLLWCDIIGQTTYRDLKL----------------------- 174

BKPYV ETLQWWVQIIGETPFRDLKL----------------------- 172

JCPYV EALHCWEKVLGDTPYRDLKL----------------------- 172

KIPyV QTFFFWKVIIFNTEIRAVQPLLR-------------------- 191

WUPyV ETFFYWKKIIFLTTMQGVGLTR--------------------- 194

HPyV12 GAFTEWLILLKHLDWRLLKISSAELDV-------LGK------ 182

MPyV V-LNLYADFIASMPIDWLDLDVHSVYNPTGLSP---------- 196

sT MLVPLYAQFLAPIPVDWLDLNVHEVYNPASGP----------- 194

NJPyV KTFEIWKHVIAQMPAALLQLSPSLF------------------ 183

MCPyV ESFDWWQKTLEETDYCLLHLHLF-------------------- 186

LiPyV ATFEDYKNLILEMDVDLLNLHC--------------------- 179

TSPyV DSLWAWSCIVGEVDFHLVNLYLRVNQGFNWGK----------- 198

HPyV6 DSFMWWTHIIYQSPLDWLGITEKLIWW---------------- 190

HPyV7 ESFMWWSHIIFQTPMDVLNLWGQLNLL---------------- 193

QPyV ESFEWWTQIIYGTQMDVINIWGQINLL---------------- 193

HPyV9 TSFNYWTLLMRNMDLSLLRLWTELGF----------------- 189

HPyV10 TSFFWWKHIMHNTEFDLLCLLGELILWVSYFSFILGKSHLWDS 206

STLPyV ASLHAWKHVMYNLDLDLLMFKQLNLVSFSF------------- 195

. : : :
