# Supplementary material for: Functional Domains of the Early Proteins and Experimental and Epidemiological Studies Suggest a Role for the Novel Human Polyomaviruses in Cancer
Source: Front Microbiol. 2022 Feb 18;13:834368. doi: 10.3389/fmicb.2022.834368 (PMC8894888; doi:10.3389/fmicb.2022.834368)
Supplement: Supplementary file 3 [file Table_3.DOCX]

**Alignment MT**

>MPyV: 431 aa

MDRVLSRADKERLLELLKLPRQLWGDFGRMQQAYKQQSLLLHPDKGGSHALMQELNSLWGTFKTEVYNLRMNLGGTGFQVRRLHADGWNLSTKDTFGDRYYQRFCRMPLTCLVNVKYSSCSCILCLLRKQHRELKDKCDARCLVLGECFCLECYMQWFGTPTRDVLNLYADFIASMPIDWLDLDVHSVYNPKRRSEELRRAATVHYTMTTGHSAMEASTSQGNGMISSESGTPATSRRLRLPSLLSNPTYSVMRSHSYPPTRVLQQIHPHILLEEDEILVLLSPMTAYPRTPPELLYPESDQDQLEPLEEEEEEYMPMEDLYLDILPGEQVPQLIPPPIIPRAGLSPWEGLILRDLQRAHFDPILDASQRMRATHRAALRAHSMQRHLRRLGRTLLLVTFLAALLGICLMLFILIKRSRHF

>STLPyV: 229 aa

MDQALSRQEAKELMGLLGLPEDSWGNVPLITYRFRQKSKIYHPDKGGNEETMKRMTELYSRMQNTLQNLRSSNENENMYPPVRMLLLTDTFTLGELLGPQFESKVIFIWPTCAKCRYRTFCQCVCCILKRQHDEIKKVRNKPCVTWGECYCFDCFLLWFGCDLTKASLHAWKHVMYNLDLDLLMFKQLNLVMMKGPLHPKKENFLILLLRIQHHLRKISQQIQQIFLLN

>TSPyV: 332 aa

MDKFLSREESLELMDLLQIPRHCYGNFALMKINHKKMSLKYHPDKGGDPEKMSRLNQLWQKLQEGIYNARQEFPTSFSSQVGSWYWEANLISLKEYFGKKKYDENVIKHWPQCAEKALKECKCLTCKIGLQHYVYKQMHQKKCVVWGECFCYKCYCAWFGEDLYCLDSLWAWSCIVGEVDFHLVNLYLRVNQGFNWVFPFSMMFQPRMEEIYLPMGTPPGPAGGKASIKNGTTCLTPCRTQTSSAMNPPFPLMNLDLQAPLRDPLLNLARRIQEEEELPRQRTPPAAPRAPSLPPPQSQKNLSMTLSLMIFLICCGLFFLMLSIVIKLYHLF

CLUSTAL O(1.2.4) multiple sequence alignment

MPyV MDRVLSRADKERLLELLKLPRQLWGDFGRMQQAYKQQSLLLHPDKGGSHALMQELNSLWG 60

STLPyV MDQALSRQEAKELMGLLGLPEDSWGNVPLITYRFRQKSKIYHPDKGGNEETMKRMTELYS 60

TSPyV MDKFLSREESLELMDLLQIPRHCYGNFALMKINHKKMSLKYHPDKGGDPEKMSRLNQLWQ 60

**: *** : .*: ** :*.. :*:. : .:: * ******. *..:..*:

MPyV TFKTEVYNLRMNLGGTG-FQVRRLHADGWNLSTKDTFGDRYYQR-FCRMPLTCLVNVKYS 118

STLPyV RMQNTLQNLRSSNENENMYPPVRMLLLTDTFTLGELLG-PQFESKVIFIWPTCAKCRYRT 119

TSPyV KLQEGIYNARQEFPTSFSSQVGSWYWEANLISLKEYFGKKKYDENVIKHWPQCAEKAL-K 119

:: : * * . :: : :* :: . * .

MPyV SCSCILCLLRKQHRELKDKCDARCLVLGECFCLECYMQWFGTPTRDVLNLYAD--FIASM 176

STLPyV FCQCVCCILKRQHDEIKKVRNKPCVTWGECYCFDCFLLWFGCDLTK-ASLHAWKHVMYNL 178

TSPyV ECKCLTCKIGLQHYVYKQMHQKKCVVWGECFCYKCYCAWFGEDLYCLDSLWAWSCIVGEV 179

*.*: * : ** *. : *:. ***:* .*: *** .* * .: .:

MPyV PIDWLDLDVHSVYNPKRRSEELR--RAATVHYTMTTGHSAMEASTSQGNGMISSESGTPA 234

STLPyV D-----LDLLMFK-----QLNLVMMK-GPLHPKKENFL---------------------- 205

TSPyV D-----FHLVNLYLRVNQGFNWVFPFSMMFQPRMEEIYLPMGTPPGPAGGKASIKNGTTC 234

:.: . : .:

MPyV TSRRLRLPSLLSNPTYSVMRSHSYPPTRVLQQIHPHILLEEDEILVLLSPMTAYPRTPPE 294

STLPyV -----------------ILLLRIQHHLRK------------------------------- 217

TSPyV LTPCRTQTSSAMNPPFPLMNLDLQAPLRD--------------------PL--------- 265

:: *

MPyV LLYPESDQDQLEPLEEEEEEYMPMEDLYLDILPGEQVPQLIPPPIIPRAGLSPWEGLILR 354

STLPyV --------------------------ISQQIQQIFLLN---------------------- 229

TSPyV ------------------------LNLARRIQEEEELPRQRTPPAAPRAPSLPPP----- 296

: * :

MPyV DLQRAHFDPILDASQRMRATHRAALRAHSMQRHLRRLGRTLLLVTFLAALLGICLMLFIL 414

STLPyV ------------------------------------------------------------ 229

TSPyV -------------------------------QSQKNLSMTLSLMIFLICCGLFFLMLSIV 325

MPyV IKRSRHF 421

STLPyV ------- 229

TSPyV IKLYHLF 332
