# Supplementary material for: Functional Domains of the Early Proteins and Experimental and Epidemiological Studies Suggest a Role for the Novel Human Polyomaviruses in Cancer
Source: Front Microbiol. 2022 Feb 18;13:834368. doi: 10.3389/fmicb.2022.834368 (PMC8894888; doi:10.3389/fmicb.2022.834368)
Supplement: Supplementary file 5 [file Data_Sheet_1.pdf]

Supplementary Figure S1. Alignment LTag

>MPyV (J02288): 785 aa
MDRVLSRADKERLLELLKLPRQLWGDFGRMQQAYKQQSLLLHPDKGGSHALMQELNSLWGTFKTEVYNLRMNLGGTGF
QGSPPRTAERGTEESGHSPLHDDYWSFSYGSKYFTREWNDFFRKWDPSYQSPPKTAESSEQPDLFCYEEPLLSPNPSS
PTDTPAHTAGRRRNPCVAEPDDSISPDPPRTPVSRKRPRPAGATGGGGGGVHANGGSVFGHPTGGTSTPAHPPPYHSQ
GGSESMGGSDSSGFAEGSFRSDPRCESENESYSQSCSQSSFNATPPKKAREDPAPSDFPSSLTGYLSHAIYSNKTFPA
FLVYSTKEKCKQLYDTIGKFRPEFKCLVHYEEGGMLFFLTMTKHRVSAVKNYCSKLCRSFLMCKAVTKPMECYQVVTA
APFQLITENKPGLHQFEFTDEPEEQKAVDWIMVADFALENNLDDPLLIMGYYLDFAKEVPSCIKCSKEETRLQIHWKN
HRKHAENADLFLNCKAQKTICQQAAASLASRRLKLVECTRSQLLKERLQQSLLRLKELGSSDALLYLAGVAWYQCLLE
DFPQTLFKMLKLLTENVPKRRNILFRGPVNSGKTGLAAALISLLGGKSLNINCPADKLAFELGVAQDQFVVCFEDVKG
QIALNKQLQPGMGVANLDNLRTTWNGSVKVNLEKKHSNKRSQLFPPCVCTMNEYLLPQTVWARFHMVLDFTCKPHLAQ
SLEKCEFLQRERIIQSGDTLALLLIWNFTSDVFDPDIQGLVKEVRDQFASECSYSLFCDILCNVQEGDDPLKDICDIA
EYTVY

>HaPyV (NC_001663): 751 aa
MDRILTKEEKQALISLLDLEPQYWGDYGRMQKCYKKKCLQLHPDKGGNEELMQQLNTLWTKLKDGLYRVRLLLGPSQD
PNASTSTSRPGEFYNPDTGGYWSYSYGSAGYSDQQKKYWEEFFSKWDVNEDLTCQEELSSSEDEFTPWHPNPPPSPVS
ISSDSSSSSCDEEYPRNSSRKRKRVHANGSPNTPIQPNKRAHTPGGGRTTIRGDTDIPRTPARESQSTFGSYFNSTEE
LEEEISQTQQSHHNTTPKKPPPTVSPDDFPTILRGFLSHAIFSNKTQNAFIIYSTKEKCEVLYEQIDKYNPDYKGIFI
MKQTEAFVMFMTPGKHRVAAVKSYCCKFCTVSFLLCKAVTKPLELYNCVAKCDDFQILKENKPGLYHFEFCDEKKEVK
QIDWNFLTSFAVENELDDPLVIMGHYLEFSQCESSCKKCAEALPRMKVHWANHSQHLENAELFLHCKQQKSICQQAAD
NVLARRRLKVLESTRQELLAERLNKLLDQLKDLSPVDKHLYLAGVAWYQCMFPDFEMMLLDILKLFTENVPKKRNVLF
RGPVNSGKTSLAAAIMNLVGGVALNVNCPADKLNFELGVAIDKFAVVFEDVKGQTGDKRHLQSGLGINNLDNLRDYLD
GSVKVNLEKKHVNKRSQIFPPCIVTANEYFFPQTLYARFHKVYNFEVKDFLAKSLEENSYMGRHRVCQSPLTMLIALL
WNVPTENFDKSLKEKVETEKKVLSDMCNFTTFAEMCLNIQRGADPLEAL

>SV40 (NC_001669): 708 aa
MDKVLNREESLQLMDLLGLERSAWGNIPLMRKAYLKKCKEFHPDKGGDEEKMKKMNTLYKKMEDGVKYAHQPDFGGFW
DATEIPTYGTDEWEQWWNAFNEENLFCSEEMPSSDDEATADSQHSTPPKKKRKVEDPKDFPSELLSFLSHAVFSNRTL
ACFAIYTTKEKAALLYKKIMEKYSVTFISRHNSYNHNILFFLTPHRHRVSAINNYAQKLCTFSFLICKGVNKEYLMYS
ALTRDPFSVIEESLPGGLKEHDFNPEEAEETKQVSWKLVTEYAMETKCDDVLLLLGMYLEFQYSFEMCLKCIKKEQPS
HYKYHEKHYANAAIFADSKNQKTICQQAVDTVLAKKRVDSLQLTREQMLTNRFNDLLDRMDIMFGSTGSADIEEWMAG
VAWLHCLLPKMDSVVYDFLKCMVYNIPKKRYWLFKGPIDSGKTTLAAALLELCGGKALNVNLPLDRLNFELGVAIDQF
LVVFEDVKGTGGESRDLPSGQGINNLDNLRDYLDGSVKVNLEKKHLNKRTQIFPPGIVTMNEYSVPKTLQARFVKQID
FRPKDYLKHCLERSEFLLEKRIIQSGIALLLMLIWYRPVAEFAQSIQSRIVEWKERLDKEFSLSVYQKMKFNVAMGIG
VLDWLRNSDDDDEDSQENADKNEDGGEKNMEDSGHETGIDSQSQGSFQAPQSSQSVHDHNQPYHICRGFTCFKKPPTP
PPEPET

>BKV (NC_001538): 695 aa
MDKVLNREESMELMDLLGLERAAWGNLPLMRKAYLRKCKEFHPDKGGDEDKMKRMNTLYKKMEQDVKVAHQPDFGTWS
SSEVPTYGTEEWESWWSSFNEKWDEDLFCHEDMFASDEEATADSQHSTPPKKKRKVEDPKDFPSDLHQFLSQAVFSNR
TLACFAVYTTKEKAQILYKKLMEKYSVTFISRHMCAGHNIIFFLTPHRHRVSAINNFCQKLCTFSFLICKGVNKEYLL
YSALTRDPYHTIEESIQGGLKEHDFSPEEPEETKQVSWKLITEYAVETKCEDVFLLLGMYLEFQYNVEECKKCQKKDQ
PYHFKYHEKHFANAIIFAESKNQKSICQQAVDTVLAKKRVDTLHMTREEMLTERFNHILDKMDLIFGAHGNAVLEQYM
AGVAWLHCLLPKMDSVIFDFLHCIVFNVPKRRYWLFKGPIDSGKTTLAAGLLDLCGGKALNVNLPMERLTFELGVAID
QYMVVFEDVKGTGAESKDLPSGHGINNLDSLRDYLDGSVKVNLEKKHLNKRTQIFPPGLVTMNEYPVPKTLQARFVRQ
IDFRPKIYLRKSLQNSEFLLEKRILQSGMTLLLLLIWFRPVADFATDIQSRIVEWKERLDSEISMYTFSRMKYNICMG
KCILDITREEDSETEDSGHGSSTESQSQCSSQVSDTSAPAEDSQRSDPHSQELHLCKGFQCFKRPKTPPPK

>JCV (NC_001699): 688 aa
MDKVLNREESMELMDLLGLDRSAWGNIPVMRKAYLKKCKELHPDKGGDEDKMKRMNFLYKKMEQGVKVAHQPDFGTWN
SSEVPTYGTDEWESWWNTFNEKWDEDLFCHEEMFASDDENTGSQHSTPPKKKKKVEDPKDFPVDLHAFLSQAVFSNRT
VASFAVYTTKEKAQILYKKLMEKYSVTFISRHGFGGHNILFFLTPHRHRVSAINNYCQKLCTFSFLICKGVNKEYLFY
SALCRQPYAVVEESIQGGLKEHDFNPEEPEETKQVSWKLVTQYALETKCEDVFLLMGMYLDFQENPQQCKKCEKKDQP
NHFNHHEKHYYNAQIFADSKNQKSICQQAVDTVAAKQRVDSIHMTREEMLVERFNFLLDKMDLIFGAHGNAVLEQYMA

GVAWIHCLLPQMDTVIYDFLKCIVLNIPKKRYWLFKGPIDSGKTTLAAALLDLCGGKSLNVNMPLERLNFELGVGIDQ
FMVVFEDVKGTGAESRDLPSGHGISNLDCLRDYLDGSVKVNLERKHQNKRTQVFPPGIVTMNEYSVPRTLQARFVRQI
DFRPKAYLRKSLSCSEYLLEKRILQSGMTLLLLLIWFRPVADFAAAIHERIVQWKERLDLEISMYTFSTMKANVGMGR
PILDFPREEDSEAEDSGHGSSTESQSQCFSQVSEASGADTQENCTFHICKGFQCFKKPKTPPPK

>KIPyV (NC_009238): 641 aa
MDKTLSREEAKQLMQLLCLDMSCWGNLPLMRRQYLVKCKEYHPDKGGNEESMKLLNSLYLKLQDSVSSVHDLNEEEDN
IWQSSQIPTYGTPDWDEWWSQFNTYWEEELRCNESMPSSPKRSAPEEEPSCSQATPPKKKHAFDASLEFPKELLEFVS
HAVFSNKCITCFVVHTTREKGEVLYKKLLQKYQCSFISKHAFYNTVLIFFLTPHKHRVSAINNFCKGHCTVSFLFCKG
VNNPYGLYSRMCRQPFNLCEENIPGGLKENEFNPEDLFGEPKEPSLSWNQIANFALEFDIDDVYYLLGSYIRFATKPE
ECEKCSKNDDATHKRVHVQNHENAVLLQESKSQKNACTQAIDRVIAERRYNCLTLTRKKLLTKRFKKLFNEMDKIVVG
ERKILLYMASIAWYTGLNKKIDELVVRFLKLIVDNKPKHRYWLFKGPINSGKTTLATALLNLCGGKALNINIPSEKLP
FELGVALDQYMVVFEDVKGQIGIEKQLPSGNGVNNLDNLRDYLDGCVEVNLEKKHVNKRSQIFPPGIVTMNEYCIPET
VAVRFEKTVMFTIKRNLRESLEKTPQLLSQRILHSGIAMLLLLIWYRPVSDFDEEIQSNVVYWKEVLDNYIGLTEFAT
MQMNVTNGKNILEKWFE

>WUPyV (NC_009539): 648 aa
MDKTLSRNEAKELMQLLGLDMTCWGNLPLMRTKYLSKCKEFHPDKGGNEEKMKKLNSLYLKLQECVSTVHQLNEEEDE
VWSSSQIPTYGTPDWDYWWSQFNSYWEEELRCNEEMPKSPGETPTKRTREDDEEPQCSQATPPKKKKDNATDASLSFP
KELEEFVSQAVFSNRTLTAFVIHTTKEKAETLYKKLLSKFKCNFASRHSYYNTALVFILTPFRHRVSAVNNFCKGYCT
ISFLFCKGVNNAYGLYSRMTRDPFTLCEENIPGGLKENDFKAEDLYGEFKDQLNWKALSEFALELGIDDVYLLLGLYL
QLSIKVEECEKCNSNEDATHNRLHMEHQKNALLFSDSKSQKNVCQQAIDVVIAKRRVDSLNMSREDLLARRFEKILDK
MDKTIKGEQDVLLYMAGVAWYLGLNGKIDELVYRYLKVIVENVPKKRYWVFKGPINSGKTTVAAALLDLCGGKALNIN
IPADRLNFELGVAIDQFTVVFEDVKGQVGDNKLLPSGNGMSNLDNLRDYLDGSVKVNLEKKHLNKRSQIFPPGIVTMN
EYLVPATLAPRFHKTVLFTPKRHLKESLDKTPELMVKRVLQSGMCILIMLIWCRPVSDFHPCIQAKVVYWKELLDKYI
GLTEFADMQMNVTNGCNILEKHNA

>MCPyV (NC_010277): 817 aa
MDLVLNRKEREALCKLLEIAPNCYGNIPLMKAAFKRSCLKHHPDKGGNPVIMMELNTLWSKFQQNIHKLRSDFSMFDE
VDEAPIYGTTKFKEWWRSGGFSFGKAYEYGPNPHGTNSRSRKPSSNASRGAPSGSSPPHSQSSSSGYGSFSASQASDS
QSRGPDIPPEHHEEPTSSSGSSSREETTNSGRESSTPNGTSVPRNSSRTDGTWEDLFCDESLSSPEPPSSSEEPEEPP
SSRSSPRQPPSSSAEEASSSQFTDEEYRSSSFTTPKTPPPFSRKRKFGGSRSSASSASSASFTSTPPKPKKNRETPVP
TDFPIDLSDYLSHAVYSNKTVSCFAIYTTSDKAIELYDKIEKFKVDFKSRHACELGCILLFITLSKHRVSAIKNFCST
FCTISFLICKGVNKMPEMYNNLCKPPYKLLQENKPLLNYEFQEKEKEASCNWNLVAEFACEYELDDHFIILAHYLDFA
KPFPCQKCENRSRLKPHKAHEAHHSNAKLFYESKSQKTICQQAADTVLAKRRLEMLEMTRTEMLCKKFKKHLERLRDL
DTIDLLYYMGGVAWYCCLFEEFEKKLQKIIQLLTENIPKYRNIWFKGPINSGKTSFAAALIDLLEGKALNINCPSDKL
PFELGCALDKFMVVFEDVKGQNSLNKDLQPGQGINNLDNLRDHLDGAVAVSLEKKHVNKKHQIFPPCIVTANDYFIPK
TLIARFSYTLHFSPKANLRDSLDQNMEIRKRRILQSGTTLLLCLIWCLPDTTFKPCLQEEIKNWKQILQSEISYGKFC
QMIENVEAGQDPLLNILIEEEGPEETEETQDSGTFSQ

>HPyV6 (NC_014406): 669 aa
MDRLLAREEVRELMDLIGLSMACWGNLPLMQQKIRLACKKYHPDKGGDPEKMQRLNVLKEKLNATLRDQMSSSPTWCF
SSEVRPPPQYGSPGWEQWWADFNRGWDDEDLYCDEHLSASEEEDNVDPGEGNSQDSKYSCTPPKKRKPNPAPNDFPSC
LHDYLSHATLGNKCYTCFVSYTTLEKWETLYDKLQSAFNAVFTGAYKCNDNTGAILYCITPRRHRVSAMLNALSKCCT
ISFLLIKAVLKSAECYMALQGDEFTVIQESRAEGLHSYDFQEGSKKEECDWNQVASFASDTDLTDCLALLGYYIEFAN
DPASCMKCKKGVKVHKHHEVHFHNAQLFKAAKNQKSIAQQACDRVAAQRRVLMLESTRQDLVLQAFKKQFTILAEQYA
GGVEITQLLGAVAWLDCLQPSFTTKLKEILSILTENIPKKRNVLFKGPINSGKTTLAAAILDLVGGVSLNVNCTPDKI
NFELGCAIDKFMCVIEDVKGTPMANTNLTQGCGMTNLDNLRDYLDGCVPVNMERKHLNKVSQLFPPSVITCNDYIIPC
TVKARIARGYYFLHKPCLQKCLKDCVLMSKRLLQKGTTLLAALIWWEPVEDFMEELQEDVVNWKQTFERWVSFGMYQT
MKENILAGIDPFTNVLVDESFVQPQENDETNDSTQESGIGSMHSM

>HPyV7 (NC_014407): 671 aa
MDKLLGRDEVKELMELIGLNMACWGNLPLIQHKVRLASKKYHPDKGGDPQKMQRLNVLKDKLQATLRDQRSGSPMWHY
SSDEVRPPPPYGSPAWDQWWQDFNKGWDEDLYCTEELSSSDEEEPAASASVNPEEGCSQDSKYSATPPKQKKPNPAPQ
DFPECLSEFLSHATLGNKCYTCFLCYTTYEKSMLLYEKLGVEFNALFIGAYNCVDGSGALVFFISGSRHRVSAILNAC
KKHCTVSFIMVKAVLKNAECYKALQDSKFAVLRESKEGGLHSYDFQEASKKDDCDWNFVADFAADMELTDVLLIMGYY

MEFATEPSLCPKCLKSVKAHQHHEKHWANAKLFKTAKNQKGIAQQAADRVLAARRVLMMESTRKDLMVMSFKKQFKVL
AEQFGGGVEITQLIGAVAWLDCLLPQFTIKIKEMLSYLVENTPKRRNLLFKGPINSGKTTLAAAILDLLGGVALNVNC
SSDKINFELGCAIDKYMVVIEDVKGTPLPNTDLPSGVGMANLDNMRDYLDGCVPVNLERKHINKTSQLFPPCIITCNE
YAIPTTVKARVAKGYYFLHKPGLKKSLDANPILMKKRLLQKGCTLLAALIWWEPVSDFVEEIQEEVVNWKQTFEQWVS
YGMFQTMKENILSGKDPFEGVLINDPTEENTRETQESTESGIGSMNN

>TSPyV (NC_014361): 697 aa
MDKFLSREESLELMDLLQIPRHCYGNFALMKINHKKMSLKYHPDKGGDPEKMSRLNQLWQKLQEGIYNARQEFPTSFS
SQHDVPTQDGRDIPPYGHPSWASWWESFNQEWDNLFDTMQDPDLFCHESTIPSDESRSPSPTPGPSTQFSEENSRRRR
AAPPEDSPGCTQSSFSATPPKPKKSKYDSVPNDFPDMLRPFLSNAVYSNKTLSSFLIYTTNEKAEYLYKKLDKFNPEF
KSRHSFQEGSMVFLMTPGKHRVSAIKNLCVTHCTVSFLLCKAVIKQVECYRCMCSEPFKLLEESKPGIFEYEFNEENG
KPVVNWNLLTDFAVTNRLDDPLLIMAHYLDFAEEPSICSKCTKKALKAHYNYHSLHHKNAKLFKECKTQKTACQQAAD
VVMAKQRLKLIESTRKELLEERFKLMFEKLTDEFGQIKILQYMAGVAWYSCLFENIDEVVTKILKLIVENVPKKRNCL
FRGPINSGKTTFAAALMNFLGGKTLNVNCPADKLPFELGCAIDQFVVIFEDVKGQIALNKKLQPGQGVSNLDNLRDHL
DGSVKVNLERKHVNKRSQIFPPCLVTMNEYLLPETIFTRFAYVLNFTPKHNLRSCLQVSDYLLTERILQDGVTIALLL
VWYCPITMFSESIKEDVKYWKDILCKYMGHTNFATLLLNVEEGKDPLDSVVIEVEDEEEEEFSETNDSGFQTQ

>HPyV9 (NC_015150): 680 aa
MDQTLSLEERNELMDLLQLTRAAWGNLSLMKKAYKTVSKIYHPDKGGNPEKMQRLNELFQKLQVTLLEIRSNCGSSSS
QGYYSDSPYFTETPFSYCERKNEDPEGGSWGKWWREFVNKEYDDLFCSETISSSDDENNPGPSAPPPSSASASEDPDP
EEEAGSSQSSFTCTPPKRKKPEPNTPEDFPMCLYSFLSHAIYSNKTMNCFLIYTTVEKSKQLYRTVEKSKIKVDFKAI
FLYKDDGIEGGLLYFITLGKHRVSAVKHFCVAQCTFSFIHCKAVIKPLELYRALGKPPFKLLEENKPGVSMFDFQEEK
EQAVNWQEICNYAVEAKITDVLLLLGIYLDFAVEPGTCSKCEKKSHKFHYNYHSKHHANACLFLESKSQKNICQQAVD
QVLAAKRLKLVECTRMELLEDRFIQLFDEMEDFLHGEIEILRWMSGVAWYTILLDNSWDVFQKILQLVTTSQPKKRNI
LFKGPINSGKTTLASAFMHFFDGKALNINCPAEKLSFELGCAIDQFCVLLDDVKGQITLNKHLQPGQGVNNLDNLRDH
LDGTIKVNLEKKHVNKRSQIFPPVIMTMNEYLLPPTVGVRFALHIHFHCKTYLKQSLEKSDLIEKRILNSGYTILLLL
LWYNPVDSFTPKVQEYVVKWKEILERHVSITQFGNIQQNILDGKDPLHGIVIEEQA

>HPyV10(JX262162)
MDRVLSRDEVKELMALLSLNTAAWGNIPLMQYKYRQTCLKLHPDKGGDGEKMKRLNELFSKMYTTIEKLRREGEVYFP
AKGNPTYGTPEWDQWWEEFNRGWDEDLSCNESFAPSDEEEPGPSQSASQTANDTNTPKKRPRESSSNSTCTPPKRPRN
FNPVDFPEVLLEFLSNAIFSNKTLNSFVLYTTREKGQFLYEKVPLKFKAMFYSLHEFDGDSLLFLLLSGKHRVSAIKN
YCSNLCTVSFLLVKGCLKAYECYYALCKTPFKLIKQSQEHGLSKTDFCEEEKDKVVNWQQICEFAVEVQCEDPLLLMG
MLLDFAKDVEGCSKCEQKKLKHHYKFHEAQNINSKLFKDCKNQKTICQQATDWVTAQRRLLILESTREHLLVLRFKHM
FEKMEDICGEVEICQYMAGVAWLSLLMPHFDEIILFIIKAMTENVPKRRYVLFKGPINSGKTTVAAAILDLLGGKTLN
VNCPPDKLAFEIGCAIDEYMVVFEDVKGQNEGSNSSLTPGMGMSNLDNLRDHLDGCVKVNLEKKHVNKKSQIFPPGII
TMNDYFIPPTLQARMIKTINFRPKLFLRNSLEKNSELLRKRIVQSGVTLLLLLCWWQPVIAFHPEIHDNVRYWKETIE
KYVPFGMYHDIRRNIESGEDPLKDILICVDADEDTQQDSGINSQ

>STLPyV (KF525270)
MDQALSREEAKELMGLLGLPEDSWGNVPLITYRFRQKSKIYHPDKGGNEETMKRMTELYSRMQNTLQNLRSSNENEHM
YPPGGQYGTPAWEQWWEEFNQPFEDDLTCNESFNCSDDEGTSASQKRKFPDYSTQNSTPPKKNKPADPTDFPAELETF
LSHAVFSNKTSNCFCIYTTMEKGNELYTVIGPKFKSMFISCHSYNTCCLLFMILAGKHRVSALKNFCSALCSISFVLV
KSCLKPYECYYRMCSSPFSVIKQSRPEGLSQAEFMEQENSKPTVNWQQICEFAVQFNCEDPLLLMGIYLDFSESPDNC
EKCRTELKHHNQFHEKEHNNAKLFRDSKTQKTLCQQACDWVCAKRRVLILESTREDLLVIRFKQVLKEMQDIAGEVEI
LRYMAGVAWLSLLFNHFDDIVLEIIRTMVVNTPKRRYFLFKGPINSGKTTVAAAILDLLGGRTLNINCPPEKVNFELG
CAIDEFMVVFEDVKGQTEGKTNLTSGMGMNNLDSLRDHLDGCVKVNLEKKHLNKRSQIFPPGIITMNEYNVPLTILAR
MVKVINFRPKHYLKKSLEVNNELLHRRIVQSGKTLLMLLMWWQPVKVFHSSIHEDVKLWKDTLTKYVSIGMFHDIQKN
IQNGEDPLKNILICEDTENNETQDSAFCTQDSDNE

>HPyV12 (NC_020890)
MDSILTFAERQLLISLLKISGDTFGNVPAMARAYKLAAKRLHPDKGGNEAEMKKLNELWNKFKDGIYNLREYNPHRNP
SGNPCGSFFWFRRFQSDLFADETLSSTSSDEEPEPAQRKRGTGANIHESASRTSFSTGSPGKGTRGGGGIPRDAPPPD
SGYGSFPFDSTPPKRGRNGGGSAPSTSGGVPDDFEGDSADQNCSQATPPKSKKAKMDNGPSDFPCDINIFLSSAVYSN
KTVNAFLIFTTVEKCQLLYQKIDVKFKIDFKSRHEGENKAHGYLYILTVAKHRVSAVKNYCAKQCTISFLHCKAINKP
FDCYKALCCDPYKRIESNKDLFQTDFENENSQQVDWTLISTFAECNMIDDPYLIMGHYLDFASPLPCNKCQMKVLKVH
YQFHEAHHNNAILFKNSKAQKTICQQAADVVIAKRRLHLIESTREELLAERFKLFLNKYKELDKMRVLEHMAGVMWYS

VMFENIDRIVIQILKLMTENIPKKRNVLFKGPINSGKTSFAAAMLDLISGKTLNINCPADKLPFELGCALDQFAVVFE
DVKGQVGNDKTLQCGQGVNNLDNIRDHLDGSVTVNLEKKHVNKKTQIFPPCIVTMNDYKLPPTVKARFAYMVIFTHMK
CLQTSLEKNDDIVKHRITHSGLTMFMILMWYCSSSAFIPSLRETIEIEKKLLESICTTEIACLMKDNIKAGRDPLHDI
VTEADE

>NJPyV-2013 (NC_024118)
MEKVLEKSDKEMLIELLGIPRYAYGNFPIMKTAYKRASKIYHPDKGGSSEKMMLLNSLWQKFQEGLIEVRDSEVFSDS
YGSANFRKRYASWCSSVFTNEKSDSRADLHCDESPISSSSDEEDETQSSGYNSFPFTSTPTPSTSTASQEVPPPFSEP
QFPESSSASGSSSAGRNTETERESPPKRRRGTEDLDGSYTDSQTSFASTPPKQKRKSPDSPSDLPSCLFDFVSHAIFS
NKTVNAFILYSTLEKASLLYEKIDKFKIEFKSLHKLTEGANVGGGLVLVMTIAKHRVSAMKNFCQQFCTVSFLICKVV
LKPLECYQCLCKPPFSQVKANKDGLFSYDFEDKKEENCNWNKVAEFAVLADIDDPLLILAHYLDFAQPFPCLKCEHQK
TKAHDYHKAHHENAVLFEACKSQRSICNQASDIVLAKRRLLLTESTREELLAMCFQKQLKALQALDTLEIYDHMAGVA
WYANLFENFDDILFQILKLLTQNIPKQRNILFRGPVNSGKTTFAAALVDLLGGRSLNVNCPADKLNFELGCAIDRFFV
VFEDVKGQNMLNKKLQPGQGISNLDNMRDYLDGAVPVNLEKKHMNKRSQVFPPCVMTMNEYFMPQTLFVRFSLKLDFV
SRPNLQSAVDKTPGLVANRILQKGLTLFLLLIWYTPVKKFAVSLQEEIANWKCIIEKTVSHSDFCKMLENIEVGESPL
TDLIDEGDN

>LiPyV (NC_034253)
MDAVLTTPERRQLCLLLDISPQEYGNIPLMKNAFKKACLKHHPDKGGDPVLMMQLNSLWGKFTTSLTEARASTYQDDP
IYGTPQFRAWWYRKHYGFFPDGFDPRRSSSTRNRRPGGTEEPEYEQPSTSGPNLSTPRPKKSRSNLFGSSGCRSRSTA
QNPLFCDESLSSSEEEAENASAKSQSDHFSFTSQEESSQASAPSFTSNESTPASTPKRNRKNQSFGGIPSPGSRRSFS
STPPKQKRYKEGDDPIDFPNCLSEFLSHATLSNKTYSCFLIFTTAEKGELLYNKVSEKYKVEFKSLHNYRGGTALLFL
VLLTRHRVTAIKNFACTFCSVSFLLCKAVIKSPELYSCLIKEPFCLLKENKPGLWDHEFAENKEPSCNWNLVADFACN
YNLTDWVIILAHYLDFANDPALCDKCSKLPLKPHEAHRKNYENAKFFLKCKSQKTICQQAADVVIAKNRLKMLEQTRE
EMLREKILCKLKELQEMKLETLYIFLAGVAWYKVMFGNFEWKVFKVLNLLTDNIPKKRNVLFKGPINSGKTSLAAAFL
DLLEGKALNINCPQDRLNFELGCAQDLFMVCFEDVKGSRGQNKDLPSGQGMHNLDNLRDHMDGSVNVNLEKKHQNKRS
QIFPPSITTCNEYIIPDTVMCRFAITITFAHKENLRTSLRKNIDMQKLRVLQRGCTLLLGLMWLLPKEKFDDEIRPEV
ERWRDAFRGDIPQAHFEKMIQNVECGLDPLEDLFVEAPADAPPAAAPEDSEAEPPVASKVPHQHENTMEEPERQQKAR
PEKDLESQDSGLFTQDSGQT

>QPyV (BK010702)
MDRLLSRDEVNELMQLIGLSMSNWGNLPLIQHKVREACKKHHPDKGGDPEKMQRLNVLKDKFAATMRDQSSGNPIWHF
SSEEVRPPPPYGTPEWDKWWHDFNRGWDEDLYCTEELSASDEEQTAEDPEEGCSQNSKYSATPPKQRKPNPAPQDFPE
CINEYLSHATLGNKCYNCFVCYTTMEKSLMLYDKLNNEFNALFIGNYKCNDGSGSIVYMITGSRHRPSAILNASKKYC
TVSFSLVKAVLKNAECYKALQGPNFTVIRESREGGLHSYDFQEASKKDDCDWNAVAEFALANDLTDPLLIMGYYLEFA
AEPSLCQKCKKGVKAHKCHELQWSNAKLFKSAKNQRGIATQAADRVLSARRVMMIESTRVDLMVMAFKKQFQVLNDQF
AGGVEITQLLGAVAWLDCLMPSFTTKLKEMLTLLVQNYAKKRNLLFKGPINSGKTTVAAGIMDLLGGVALNVNCSSDK
INFELGCAIDKMLVVFEDVKGQPLPNTDLPAGVGMANLDNLRDHLDGCVPVNLERKHTNKVSQLFPPCIITCNDYAIP
RTVKARVAKGYYFIHKPNLKKCLDVNPILMQKRLLQKGVTLLAALIWWEPVSEFVEEIQEDVVNWKQTFERWVTYGMY
QDMKQNILAGKDPFYGVIMSDINEIVEETQESTESGVGSMET

```
CLUSTAL O(1.2.4) multiple sequence alignment


HPyV6       MDRLLAREEVRELMDLIGLSMACWGNLPLMQQKIRLACKKYHPDKGGDPEKMQRLNVLKE    60
HPyV7       MDKLLGRDEVKELMELIGLNMACWGNLPLIQHKVRLASKKYHPDKGGDPQKMQRLNVLKD    60
QPyV        MDRLLSRDEVNELMQLIGLSMSNWGNLPLIQHKVREACKKHHPDKGGDPEKMQRLNVLKD    60
SV40        MDKVLNREESLQLMDLLGLERSAWGNIPLMRKAYLKKCKEFHPDKGGDEEKMKKMNTLYK    60
BKV         MDKVLNREESMELMDLLGLERAAWGNLPLMRKAYLRKCKEFHPDKGGDEDKMKRMNTLYK    60
JCV         MDKVLNREESMELMDLLGLDRSAWGNIPVMRKAYLKKCKELHPDKGGDEDKMKRMNFLYK    60
KIPyV       MDKTLSREEAKQLMQLLCLDMSCWGNLPLMRRQYLVKCKEYHPDKGGNEESMKLLNSLYL    60
WUPyV       MDKTLSRNEAKELMQLLGLDMTCWGNLPLMRTKYLSKCKEFHPDKGGNEEKMKKLNSLYL    60
HPyV10      MDRVLSRDEVKELMALLSLNTAAWGNIPLMQYKYRQTCLKLHPDKGGDGEKMKRLNELFS    60
STLPyV      MDQALSREEAKELMGLLGLPEDSWGNVPLITYRFRQKSKIYHPDKGGNEETMKRMTELYS    60
TSPyV       MDKFLSREESLELMDLLQIPRHCYGNFALMKINHKKMSLKYHPDKGGDPEKMSRLNQLWQ    60
HPyV9       MDQTLSLEERNELMDLLQLTRAAWGNLSLMKKAYKTVSKIYHPDKGGNPEKMQRLNELFQ    60
HPyV12      MDSILTFAERQLLISLLKISGDTFGNVPAMARAYKLAAKRLHPDKGGNEAEMKKLNELWN    60
LiPyV       MDAVLTTPERRQLCLLLDISPQEYGNIPLMKNAFKKACLKHHPDKGGDPVLMMQLNSLWG    60
MCPyV       MDLVLNRKEREALCKLLEIAPNCYGNIPLMKAAFKRSCLKHHPDKGGNPVIMMELNTLWS    60
NJPyV       MEKVLEKSDKEMLIELLGIPRYAYGNFPIMKTAYKRASKIYHPDKGGSSEKMMLLNSLWQ    60
MPyV        MDRVLSRADKERLLELLKLPRQLWGDFGRMQQAYKQQSLLLHPDKGGSHALMQELNSLWG    60
HaPyV       MDRILTKEEKQALISLLDLEPQYWGDYGRMQKCYKKKCLQLHPDKGGNEELMQQLNTLWT    60
            *:  *   :   *  *: :    :*:   :       .   ******.   *  :. *

HPyV6       KLNATLRDQMSSSPTWCFS--------------S-EVRPPPQYGSPGWEQWWAD------    99
HPyV7       KLQATLRDQRSGSPMWHYS--------------SDEVRPPPPYGSPAWDQWWQD------    100
QPyV        KFAATMRDQSSGNPIWHFS--------------SEEVRPPPPYGTPEWDKWWHD------    100
SV40        KMEDGVKYAHQPD--FGG---------------FWDATEIPTYGTDEWEQWWNA------    97
BKV         KMEQDVKVAHQPD--FG----------------TWSSSEVPTYGTEEWESWWSS------    96
JCV         KMEQGVKVAHQPD--FG----------------TWNSSEVPTYGTDEWESWWNT------    96
KIPyV       KLQDSVSSVHDLNEEEDN---------------IWQSSQIPTYGTPDWDEWWSQ------    99
WUPyV       KLQECVSTVHQLNEEEDE---------------VWSSSQIPTYGTPDWDYWWSQ------    99
HPyV10      KMYTTIEKLRREGEVY------------------FPAKGNPTYGTPEWDQWWEE------    96
STLPyV      RMQNTLQNLRSSNENE------------------HMYPPGGQYGTPAWEQWWEE------    96
TSPyV       KLQEGIYNARQEFPTSFSSQH--DVPT-------QDGRDIPPYGHPSWASWWES------    105
HPyV9       KLQVTLLEIRSNCGSSSSQGYYSDSPYFTETPFSYCERKNEDPEGGSWGKWWRE------    114
HPyV12      KFKDGIYNLREYNPHR-----------------------NPSGNPCGSFFWFRRFQSDLF    97
LiPyV       KFTTSLTEARAST-----YQ------------------DDPIYGTPQFRAWWYRKHYGFF    97
MCPyV       KFQQNIHKLRSDFSMFDEVD------------------EAPIYGTTKFKEW---WRSGGF    99
NJPyV       KFQEGLIEVRDSEVFSD------------------------SYGSANFRKRYASWCSSVF    96
MPyV        TFKTEVYNLRMNLGGTGFQGS----PP----RTAERGTEESGHSPLHDDYWSFSYGSKYF    112
HaPyV       KLKDGLYRVRLLLGPSQDPNA----ST----STSR---PGEFYNPDTGGYWSYSYGSAGY    109
             :   :

HPyV6       ----------FNRGWD--------------------------------------------    105
HPyV7       ----------FNKGWD--------------------------------------------    106
QPyV        ----------FNRGWD--------------------------------------------    106
SV40        ----------FNE-----------------------------------------------    100
BKV         ----------FNEKWD--------------------------------------------    102
JCV         ----------FNEKWD--------------------------------------------    102
KIPyV       ----------FNTYWE--------------------------------------------    105
WUPyV       ----------FNSYWE--------------------------------------------    105
HPyV10      ----------FNRGWD--------------------------------------------    102
STLPyV      ----------FNQPFE--------------------------------------------    102
TSPyV       ----------FNQEWDNLFDTM--------------------------------------    117
HPyV9       ----------FVNK----------------------------------------------    118
HPyV12      ADETLSSTSSD-------EEPEPAQRKRGTGANIHESASRTSFSTGSPGKGTRGGGGIPR    150
LiPyV       PD---------------------------------GFDPRRS--SSTRNR-RPGGTEEPE    121
MCPyV       SFGKAYEYG----------------------PNPHGTNSRSRKPSSNASRGAPSGSSPP-    136
NJPyV       TNEKSD--------------------------------SRA---DLHCDESPISSSSDE-    120
MPyV        T----REWNDFFRKWDPSYQSPPKTAESSEQPD--LFCYEE------PLL-SPNPSS-PT    158
HaPyV       SDQQKKYWEEFFSKWDVNEDLTCQEELSSSEDE---FTPWH------PNP-PPSPVS-IS    158
```

```
HPyV6       ------------------------------------------------------------   105
HPyV7       ------------------------------------------------------------   106
QPyV        ------------------------------------------------------------   106
SV40        ------------------------------------------------------------   100
BKV         ------------------------------------------------------------   102
JCV         ------------------------------------------------------------   102
KIPyV       ------------------------------------------------------------   105
WUPyV       ------------------------------------------------------------   105
HPyV10      ------------------------------------------------------------   102
STLPyV      ------------------------------------------------------------   102
TSPyV       ------------------------------------------------------------   117
HPyV9       ------------------------------------------------------------   118
HPyV12      DAPPPDSGYGSFPFDSTPPKRG-------R------------------------------   173
LiPyV       YEQPSTSGPNLS----TPR---------------P--KKSR--SNLFGSSGC--------   150
MCPyV       HSQSSSSGYGSFSASQASDSQS-------RGPDIP--PEHH--EEPTSSSGSSSREETTN   185
NJPyV       EDETQSSGYNSFPFTSTP------------------------------------------   138
MPyV        DTPAHTAGRRRNPCVAEPDDS--------ISPDPPRTPVSRKRPRPAGATGG--------   202
HaPyV       ------SDSSSSSCDEEYPRNSSRKRKRVHANGSPNTPIQ---PNKRAHTPG--------   201

HPyV6       -----------------------DEDLYCDEHLSASEEEDN-------------------   123
HPyV7       ------------------------EDLYCTEELSSSDEEEPAAS----------------   126
QPyV        ------------------------EDLYCTEELSASDEEQTA------------------   124
SV40        ------------------------ENLFCSEEMPSSDDEAT-------------------   117
BKV         ------------------------EDLFCHEDMFASDEEAT-------------------   119
JCV         ------------------------EDLFCHEEMFASDDENT-------------------   119
KIPyV       ------------------------EELRCNESMPSSPKRS--------------------   121
WUPyV       ------------------------EELRCNEEMPKSPGETPTKR----------------   125
HPyV10      ------------------------EDLSCNESFAPSDEEE-PGPS-----------QSAS   126
STLPyV      ------------------------DDLTCNESFNCSDDEG-TSAS-----------QKR-   125
TSPyV       ----------------------QDPDLFCHESTIPSDESRSPSPT-----------PGPS   144
HPyV9       ----------------------EYDDLFCSETISSSDDENNPGPS-----------APPP   145
HPyV12      ------------------------------------------------------------   173
LiPyV       -----------------RSRSTAQNPLFCDESLSSSEEEAENASAKSQSDHFSFTSQEES   193
MCPyV       SGRESSTPNGTSVPRNSSRTDGTWEDLFCDESLSSPEPPSSSEEPEEPPSSRSSPRQPPS   245
NJPyV       ------------------------------------TP-STSTASQEVPPPFSEPQFPES   161
MPyV        -GGGGVHANGGSV--------------FG-------HPTGGTSTPAHPP-PYH--SQGGS   237
HaPyV       -G-GRTTIRG------------------------------DTDIPRTPA--RE--SQS--   223

HPyV6       --------------------------VDPG-------------EGNSQDSKYSCTPPKKR   144
HPyV7       ------------------------ASVNPE-------------EGCSQDSKYSATPPKQK   149
QPyV        --------------------------EDPE-------------EGCSQNSKYSATPPKQR   145
SV40        --------------------------------------------A----DSQHSTPPKKK   129
BKV         --------------------------------------------A----DSQHSTPPKKK   131
JCV         -------------------------------------------------GSQHSTPPKKK   130
KIPyV       --------------------------APEE-------------EP----SCSQATPPKKK   138
WUPyV       ------------------------TREDDE-------------EP----QCSQATPPKKK   144
HPyV10      QT-------A--------N----DTNTPKK-------------RPRESSSNSTCTPPKRP   154
STLPyV      --------------------------------------------KFPDYSTQNSTPPKKN   141
TSPyV       TQFSEENSRR--------R----RAAPPED-------------SPGCTQSSFSATPPKPK   179
HPyV9       SSAS-----A--------S----EDPDPEE-------------EAGSSQSSFTCTPPKRK   175
HPyV12      ---NGGGSAPSTS-----------G------GVPDDFEG-----DSADQNCSQATPPKSK   208
LiPyV       SQAS---APSFTS-----NE-STPASTPKRNRKNQSFGGI---PSPGSRRSFSSTPPKQK   241
MCPyV       SSAEEASSSQFTDEEYRSSSFTTPKTPPPF-SRKRKFGGSRSSASSASSASFTSTPPKPK   304
NJPyV       SSASGSSSAGRNT--------ETERESPPK-RRRG--TEDLDGSYTDSQTSFASTPPKQK   210
MPyV        ESMGGSDSSGFAEGSFRSD--------PRCE------SENESYSQSCSQSSFNATPPK--   281
HaPyV       -TF---------GSYFNST--------EELE------E-----EISQTQQSHHNTTPK--   252
                                                                  * **

HPyV6       KPN--PAPNDFPSCLHDYLSHATLGNKCYTCFVSYTTLEKWETLYDKL-----QSAFNAV   197
HPyV7       KPN--PAPQDFPECLSEFLSHATLGNKCYTCFLCYTTYEKSMLLYEKL-----GVEFNAL   202
QPyV        KPN--PAPQDFPECINEYLSHATLGNKCYNCFVCYTTMEKSLMLYDKL-----NNEFNAL   198
```

```
SV40        R--KVEDPKDFPSELLSFLSHAVFSNRTLACFAIYTTKEKAALLYKKI-----MEKYSVT    182
BKV         R--KVEDPKDFPSDLHQFLSQAVFSNRTLACFAVYTTKEKAQILYKKL-----MEKYSVT    184
JCV         K--KVEDPKDFPVDLHAFLSQAVFSNRTVASFAVYTTKEKAQILYKKL-----MEKYSVT    183
KIPyV       --HAFDASLEFPKELLEFVSHAVFSNKCITCFVVHTTREKGEVLYKKL-----LQKYQCS    191
WUPyV       KDNATDASLSFPKELEEFVSQAVFSNRTLTAFVIHTTKEKAETLYKKL-----LSKFKCN    199
HPyV10      RN---FNPVDFPEVLLEFLSNAIFSNKTLNSFVLYTTREKGQFLYEKV-----PLKFKAM    206
STLPyV      KP---ADPTDFPAELETFLSHAVFSNKTSNCFCIYTTMEKGNELYTVI-----GPKFKSM    193
TSPyV       KSKYDSVPNDFPDMLRPFLSNAVYSNKTLSSFLIYTTNEKAEYLYKKLDKFN------PE    233
HPyV9       KPE-PNTPEDFPMCLYSFLSHAIYSNKTMNCFLIYTTVEKSKQLYRTVEKSKIKVDFKAI    234
HPyV12      KAKMDNGPSDFPCDINIFLSSAVYSNKTVNAFLIFTTVEKCQLLYQKI-----DVKFKID    263
LiPyV       RYKEGDDPIDFPNCLSEFLSHATLSNKTYSCFLIFTTAEKGELLYNKV-----SEKYKVE    296
MCPyV       KNRETPVPTDFPIDLSDYLSHAVYSNKTVSCFAIYTTSDKAIELYDKI------EKFKVD    358
NJPyV       R-KSPDSPSDLPSCLFDFVSHAIFSNKTVNAFILYSTLEKASLLYEKI------DKFKIE    263
MPyV        KAREDPAPSDFPSSLTGYLSHAIYSNKTFPAFLVYSTKEKCKQLYDTI------GKFRPE    335
HaPyV       KPPPTVSPDDFPTILRGFLSHAIFSNKTQNAFIIYSTKEKCEVLYEQI------DKYNPD    306
                     .:*  :  ::* *  .*:   .*  .:* :*   **  :

HPyV6       FTGAYKCND---NTGAILYCITPRRHRVSAMLNALSKCCTISFLLIKAVLKSAECYMALQ    254
HPyV7       FIGAYNCVD---GSGALVFFISGSRHRVSAILNACKKHCTVSFIMVKAVLKNAECYKALQ    259
QPyV        FIGNYKCND---GSGSIVYMITGSRHRPSAILNASKKYCTVSFSLVKAVLKNAECYKALQ    255
SV40        FISRHNS-----YNHNILFFLTPHRHRVSAINNYAQKLCTFSFLICKGVNKEYLMYSALT    237
BKV         FISRHMC-----AGHNIIFFLTPHRHRVSAINNFCQKLCTFSFLICKGVNKEYLLYSALT    239
JCV         FISRHGF-----GGHNILFFLTPHRHRVSAINNYCQKLCTFSFLICKGVNKEYLFYSALC    238
KIPyV       FISKHAF-----YNTVLIFFLTPHKHRVSAINNFCKGHCTVSFLFCKGVNNPYGLYSRMC    246
WUPyV       FASRHSY-----YNTALVFILTPFRHRVSAVNNFCKGYCTISFLFCKGVNNAYGLYSRMT    254
HPyV10      FYSLHEF-----DGDSLLFLLLSGKHRVSAIKNYCSNLCTVSFLLVKGCLKAYECYYALC    261
STLPyV      FISCHSY-----NTCCLLFMILAGKHRVSALKNFCSALCSISFVLVKSCLKPYECYYRMC    248
TSPyV       FKSRHSF-----QEGSMVFLMTPGKHRVSAIKNLCVTHCTVSFLLCKAVIKQVECYRCMC    288
HPyV9       FLYKDDG-----IEGGLLYFITLGKHRVSAVKHFCVAQCTFSFIHCKAVIKPLELYRALG    289
HPyV12      FKSRHEG---ENKAHGYLYILTVAKHRVSAVKNYCAKQCTISFLHCKAINKPFDCYKALC    320
LiPyV       FKSLHNYRGG----TALLFLVLLTRHRVTAIKNFACTFCSVSFLLCKAVIKSPELYSCLI    352
MCPyV       FKSRHACEL-----GCILLFITLSKHRVSAIKNFCSTFCTISFLICKGVNKMPEMYNNLC    413
NJPyV       FKSLHKLTEGANVGGGLVLVMTIAKHRVSAMKNFCQQFCTVSFLICKVVLKPLECYQCLC    323
MPyV        FKCLVHYE-E----GGMLFFLTMTKHRVSAVKNYCSKLCR-SFLMCKAVTKPMECYQVVT    389
HaPyV       YKGIFIMKQT----EAFVMFMTPGKHRVAAVKSYCCKFCTVSFLLCKAVTKPLELYNCVA    362
            :                :  :   :** :*:       *  **     * :    *  :

HPyV6       -GDEFTVIQESRAEGLHSYDFQEGSK-----KEECDWNQVASFASDTDLTDCLALLGYYI    308
HPyV7       -DSKFAVLRESKEGGLHSYDFQEASK-----KDDCDWNFVADFAADMELTDVLLIMGYYM    313
QPyV        -GPNFTVIRESREGGLHSYDFQEASK-----KDDCDWNAVAEFALANDLTDPLLIMGYYL    309
SV40        -RDPFSVIEESLPGGLKEHDFNPEEAE---ETKQVSWKLVTEYAMETKCDDVLLLLGMYL    293
BKV         -RDPYHTIEESIQGGLKEHDFSPEEPE---ETKQVSWKLITEYAVETKCEDVFLLLGMYL    295
JCV         -RQPYAVVEESIQGGLKEHDFNPEEPE---ETKQVSWKLVTQYALETKCEDVFLLMGMYL    294
KIPyV       -RQPFNLCEENIPGGLKENEFNPEDLFGEPKEPSLSWNQIANFALEFDIDDVYYLLGSYI    305
WUPyV       -RDPFTLCEENIPGGLKENDFKAEDLYGEFKD-QLNWKALSEFALELGIDDVYLLLGLYL    312
HPyV10      -KTPFKLIKQSQEHGLSKTDFCEEEK-----DKVVNWQQICEFAVEVQCEDPLLLMGMLL    315
STLPyV      -SSPFSVIKQSRPEGLSQAEFMEQEN----SKPTVNWQQICEFAVQFNCEDPLLLMGIYL    303
TSPyV       -SEPFKLLEESKP-GIFEYEFNE-EN----GKPVVNWNLLTDFAVTNRLDDPLLIMAHYL    341
HPyV9       -KPPFKLLEENKP-GVSMFDFQE-EK----E-QAVNWQEICNYAVEAKITDVLLLLGIYL    341
HPyV12      -CDPYKRIESNKD--LFQTDFEN-EN----S-QQVDWTLISTFAECNMIDDPYLIMGHYL    371
LiPyV       -KEPFCLLKENKP-GLWDHEFAE-NK----E-PSCNWNLVADFACNYNLTDWVIILAHYL    404
MCPyV       -KPPYKLLQENKP--LLNYEFQEKEK----E-ASCNWNLVAEFACEYELDDHFIILAHYL    465
NJPyV       -KPPFSQVKANKD-GLFSYDFED-KK----E-ENCNWNKVAEFAVLADIDDPLLILAHYL    375
MPyV        -AAPFQLITENKP-GLHQFEFTDEPE----EQKAVDWIMVADFALENNLDDPLLIMGYYL    443
HaPyV       KCDDFQILKENKP-GLYHFEFCDEKK----EVKQIDWNFLTSFAVENELDDPLVIMGHYL    417
                :     .    :   :*              .*  :  :*      *   ::.  :

HPyV6       EFANDPASCMKCKKGV--KV--HKHHEVHFHNAQLFKAAKNQKSIAQQACDRVAAQRRVL    364
HPyV7       EFATEPSLCPKCLKSV--KA--HQHHEKHWANAKLFKTAKNQKGIAQQAADRVLAARRVL    369
QPyV        EFAAEPSLCQKCKKGV--KA--HKCHELQWSNAKLFKSAKNQRGIATQAADRVLSARRVM    365
SV40        EFQYSFEMCLKCIKKE--QPSHYKYHEKHYANAAIFADSKNQKTICQQAVDTVLAKKRVD    351
BKV         EFQYNVEECKKCQKKD--QPYHFKYHEKHFANAIIFAESKNQKSICQQAVDTVLAKKRVD    353
JCV         DFQENPQQCKKCEKKD--QPNHFNHHEKHYYNAQIFADSKNQKSICQQAVDTVAAKQRVD    352
```

```
KIPyV       RFATKPEECEKCSKND--DATHKRVHVQNHENAVLLQESKSQKNACTQAIDRVIAERRYN    363
WUPyV       QLSIKVEECEKCNSNE--DATHNRLHMEHQKNALLFSDSKSQKNVCQQAIDVVIAKRRVD    370
HPyV10      DFAKDVEGCSKCEQKK--LKHHYKFHEAQNINSKLFKDCKNQKTICQQATDWVTAQRRLL    373
STLPyV      DFSESPDNCEKCRT-E--LKHHNQFHEKEHNNAKLFRDSKTQKTLCQQACDWVCAKRRVL    360
TSPyV       DFAEEPSICSKCTKKA--LKAHYNYHSLHHKNAKLFKECKTQKTACQQAADVVMAKQRLK    399
HPyV9       DFAVEPGTCSKCEKKS--HKFHYNYHSKHHANACLFLESKSQKNICQQAVDQVLAAKRLK    399
HPyV12      DFAS-PLPCNKCQMKV--LKVHYQFHEAHHNNAILFKNSKAQKTICQQAADVVIAKRRLH    428
LiPyV       DFANDPALCDKCSKLP---LKPHEAHRKNYENAKFFLKCKSQKTICQQAADVVIAKNRLK    461
MCPyV       DFAK-PFPCQKCENRS--RLKPHKAHEAHHSNAKLFYESKSQKTICQQAADTVLAKRRLE    522
NJPyV       DFAQ-PFPCLKCEHQK---TKAHDYHKAHHENAVLFEACKSQRSICNQASDIVLAKRRLL    431
MPyV        DFAKEVPSCIKCSKEETRLQIHWKNHRKHAENADLFLNCKAQKTICQQAAAS-LASRRLK    502
HaPyV       EFSQCESSCKKCAEALPRMKVHWANHSQHLENAELFLHCKQQKSICQQAADNVLARRRLK    477
             :      * **             *  .  *: ::  .* *:  .   **     : .*

HPyV6       MLESTRQDLVLQAFKKQFTILAEQYA--GGVEITQLLGAVAWLDCLQPSFTTKLKEILSI    422
HPyV7       MMESTRKDLMVMSFKKQFKVLAEQFG--GGVEITQLIGAVAWLDCLLPQFTIKIKEMLSY    427
QPyV        MIESTRVDLMVMAFKKQFQVLNDQFA--GGVEITQLLGAVAWLDCLMPSFTTKLKEMLTL    423
SV40        SLQLTREQMLTNRFNDLLDRMDIMFGSTGSADIEEWMAGVAWLHCLLPKMDSVVYDFLKC    411
BKV         TLHMTREEMLTERFNHILDKMDLIFGAHGNAVLEQYMAGVAWLHCLLPKMDSVIFDFLHC    413
JCV         SIHMTREEMLVERFNFLLDKMDLIFGAHGNAVLEQYMAGVAWIHCLLPQMDTVIYDFLKC    412
KIPyV       CLTLTRKKLLTKRFKKLFNEMDKIVV--GERKILLYMASIAWYTGLNKKIDELVVRFLKL    421
WUPyV       SLNMSREDLLARRFEKILDKMDKTIK--GEQDVLLYMAGVAWYLGLNGKIDELVYRYLKV    428
HPyV10      ILESTREHLLVLRFKHMFEKMEDI-C--GEVEICQYMAGVAWLSLLMPHFDEIILFIIKA    430
STLPyV      ILESTREDLLVIRFKQVLKEMQDI-A--GEVEILRYMAGVAWLSLLFNHFDDIVLEIIRT    417
TSPyV       LIESTRKELLEERFKLMFEKLTDE-F--GQIKILQYMAGVAWYSCLFENIDEVVTKILKL    456
HPyV9       LVECTRMELLEDRFIQLFDEMEDFLH--GEIEILRWMSGVAWYTILLDNSWDVFQKILQL    457
HPyV12      LIESTREELLAERFKLFLNKYKEL----DKMRVLEHMAGVMWYSVMFENIDRIVIQILKL    484
LiPyV       MLEQTREEMLREKILCKLKELQEM----KLETLYIFLAGVAWYKVMFGNFEWKVFKVLNL    517
MCPyV       MLEMTRTEMLCKKFKKHLERLRDL----DTIDLLYYMGGVAWYCCLFEEFEKKLQKIIQL    578
NJPyV       LTESTREELLAMCFQKQLKALQAL----DTLEIYDHMAGVAWYANLFENFDDILFQILKL    487
MPyV        LVECTRSQLLKERLQQSLLRLKEL----GSSDALLYLAGVAWYQCLLEDFPQTLFKMLKL    558
HaPyV       VLESTRQELLAERLNKLLDQLKDL----SPVDKHLYLAGVAWYQCMFPDFEMMLLDILKL    533
                :* .::   :   :                  :..: *   :       .   :

HPyV6       LTENIPKKRNVLFKGPINSGKTTLAAAILDLVGGVSLNVNCTPDKINFELGCAIDKFMCV    482
HPyV7       LVENTPKRRNLLFKGPINSGKTTLAAAILDLLGGVALNVNCSSDKINFELGCAIDKYMVV    487
QPyV        LVQNYAKKRNLLFKGPINSGKTTVAAGIMDLLGGVALNVNCSSDKINFELGCAIDKMLVV    483
SV40        MVYNIPKKRYWLFKGPIDSGKTTLAAALLELCGGKALNVNLPLDRLNFELGVAIDQFLVV    471
BKV         IVFNVPKRRYWLFKGPIDSGKTTLAAGLLDLCGGKALNVNLPMERLTFELGVAIDQYMVV    473
JCV         IVLNIPKKRYWLFKGPIDSGKTTLAAALLDLCGGKSLNVNMPLERLNFELGVGIDQFMVV    472
KIPyV       IVDNKPKHRYWLFKGPINSGKTTLATALLNLCGGKALNINIPSEKLPFELGVALDQYMVV    481
WUPyV       IVENVPKKRYWVFKGPINSGKTTVAAALLDLCGGKALNINIPADRLNFELGVAIDQFTVV    488
HPyV10      MTENVPKRRYVLFKGPINSGKTTVAAAILDLLGGKTLNVNCPPDKLAFEIGCAIDEYMVV    490
STLPyV      MVVNTPKRRYFLFKGPINSGKTTVAAAILDLLGGRTLNINCPPEKVNFELGCAIDEFMVV    477
TSPyV       IVENVPKKRNCLFRGPINSGKTTFAAALMNFLGGKTLNVNCPADKLPFELGCAIDQFVVI    516
HPyV9       VTTSQPKKRNILFKGPINSGKTTLASAFMHFFDGKALNINCPAEKLSFELGCAIDQFCVL    517
HPyV12      MTENIPKKRNVLFKGPINSGKTSFAAAMLDLISGKTLNINCPADKLPFELGCALDQFAVV    544
LiPyV       LTDNIPKKRNVLFKGPINSGKTSLAAAFLDLLEGKALNINCPQDRLNFELGCAQDLFMVC    577
MCPyV       LTENIPKYRNIWFKGPINSGKTSFAAALIDLLEGKALNINCPSDKLPFELGCALDKFMVV    638
NJPyV       LTQNIPKQRNILFRGPVNSGKTTFAAALVDLLGGRSLNVNCPADKLNFELGCAIDRFFVV    547
MPyV        LTENVPKRRNILFRGPVNSGKTGLAAALISLLGGKSLNINCPADKLAFELGVAQDQFVVC    618
HaPyV       FTENVPKKRNVLFRGPVNSGKTSLAAAIMNLVGGVALNVNCPADKLNFELGVAIDKFAVV    593
            .. .  * *   *:**::**** .*:.:: :  * :**:*   ::: **:* . *

HPyV6       IEDVKGTPMA-NTNLTQGCGMTNLDNLRDYLDGCVPVNMERKHLNKVSQLFPPSVITCND    541
HPyV7       IEDVKGTPLP-NTDLPSGVGMANLDNMRDYLDGCVPVNLERKHINKTSQLFPPCIITCNE    546
QPyV        FEDVKGQPLP-NTDLPAGVGMANLDNLRDHLDGCVPVNLERKHTNKVSQLFPPCIITCND    542
SV40        FEDVKGTGGE-SRDLPSGQGINNLDNLRDYLDGSVKVNLEKKHLNKRTQIFPPGIVTMNE    530
BKV         FEDVKGTGAE-SKDLPSGHGINNLDSLRDYLDGSVKVNLEKKHLNKRTQIFPPGLVTMNE    532
JCV         FEDVKGTGAE-SRDLPSGHGISNLDCLRDYLDGSVKVNLERKHQNKRTQVFPPGIVTMNE    531
KIPyV       FEDVKGQIGI-EKQLPSGNGVNNLDNLRDYLDGCVEVNLEKKHVNKRSQIFPPGIVTMNE    540
WUPyV       FEDVKGQVGD-NKLLPSGNGMSNLDNLRDYLDGSVKVNLEKKHLNKRSQIFPPGIVTMNE    547
HPyV10      FEDVKGQNEGSNSSLTPGMGMSNLDNLRDHLDGCVKVNLEKKHVNKKSQIFPPGIITMND    550
```

```
STLPyV      FEDVKGQTEGK-TNLTSGMGMNNLDSLRDHLDGCVKVNLEKKHLNKRSQIFPPGIITMNE    536
TSPyV       FEDVKGQIAL-NKKLQPGQGVSNLDNLRDHLDGSVKVNLERKHVNKRSQIFPPCLVTMNE    575
HPyV9       LDDVKGQITL-NKHLQPGQGVNNLDNLRDHLDGTIKVNLEKKHVNKRSQIFPPVIMTMNE    576
HPyV12      FEDVKGQVGN-DKTLQCGQGVNNLDNIRDHLDGSVTVNLEKKHVNKKTQIFPPCIVTMND    603
LiPyV       FEDVKGSRGQ-NKDLPSGQGMHNLDNLRDHMDGSVNVNLEKKHQNKRSQIFPPSITTCNE    636
MCPyV       FEDVKGQNSL-NKDLQPGQGINNLDNLRDHLDGAVAVSLEKKHVNKKHQIFPPCIVTAND    697
NJPyV       FEDVKGQNML-NKKLQPGQGISNLDNMRDYLDGAVPVNLEKKHMNKRSQVFPPCVMTMNE    606
MPyV        FEDVKGQIAL-NKQLQPGMGVANLDNLRTTWNGSVKVNLEKKHSNKRSQLFPPCVCTMNE    677
HaPyV       FEDVKGQTGD-KRHLQSGLGINNLDNLRDYLDGSVKVNLEKKHVNKRSQIFPPCIVTANE    652
            ::****         *  * *: *** :*   :* : *.:*:** **  *:*** : * *:

HPyV6       YIIPCTVKARIARGYYFLHKPCLQKCLKD-CVLMSKRLLQKGTTLLAALIWWEPVEDFME    600
HPyV7       YAIPTTVKARVAKGYYFLHKPGLKKSLDANPILMKKRLLQKGCTLLAALIWWEPVSDFVE    606
QPyV        YAIPRTVKARVAKGYYFIHKPNLKKCLDVNPILMQKRLLQKGVTLLAALIWWEPVSEFVE    602
SV40        YSVPKTLQARFVKQIDFRPKDYLKHCLERSEFLLEKRIIQSGIALLLMLIWYRPVAEFAQ    590
BKV         YPVPKTLQARFVRQIDFRPKIYLRKSLQNSEFLLEKRILQSGMTLLLLLIWFRPVADFAT    592
JCV         YSVPRTLQARFVRQIDFRPKAYLRKSLSCSEYLLEKRILQSGMTLLLLLIWFRPVADFAA    591
KIPyV       YCIPETVAVRFEKTVMFTIKRNLRESLEKTPQLLSQRILHSGIAMLLLLIWYRPVSDFDE    600
WUPyV       YLVPATLAPRFHKTVLFTPKRHLKESLDKTPELMVKRVLQSGMCILIMLIWCRPVSDFHP    607
HPyV10      YFIPPTLQARMIKTINFRPKLFLRNSLEKNSELLRKRIVQSGVTLLLLLCWWQPVIAFHP    610
STLPyV      YNVPLTILARMVKVINFRPKHYLKKSLEVNNELLHRRIVQSGKTLLMLLMWWQPVKVFHS    596
TSPyV       YLLPETIFTRFAYVLNFTPKHNLRSCLQVSDYLLTERILQDGVTIALLLVWYCPITMFSE    635
HPyV9       YLLPPTVGVRFALHIHFHCKTYLKQSLEKSD-LIEKRILNSGYTILLLLLWYNPVDSFTP    635
HPyV12      YKLPPTVKARFAYMVIFTHMKCLQTSLEKNDDIVKHRITHSGLTMFMILMWYCSSSAFIP    663
LiPyV       YIIPDTVMCRFAITITFAHKENLRTSLRKNIDMQKLRVLQRGCTLLLGLMWLLPKEKFDD    696
MCPyV       YFIPKTLIARFSYTLHFSPKANLRDSLDQNMEIRKRRILQSGTTLLLCLIWCLPDTTFKP    757
NJPyV       YFMPQTLFVRFSLKLDFVSRPNLQSAVDKTPGLVANRILQKGLTLFLLLIWYTPVKKFAV    666
MPyV        YLLPQTVWARFHMVLDFTCKPHLAQSLEKCEFLQRERIIQSGDTLALLLIWNFTSDVFDP    737
HaPyV       YFFPQTLYARFHKVYNFEVKDFLAKSLEENSYMGRHRVCQSPLTMLIALLWNVPTENFDK    712
            * .* *:  *.      *     *  .:      :   *: :    :   * *      *

HPyV6       ELQEDVVNWKQTFERWVSFGMYQTMKENILAGIDPFTNVLVDESFVQPQENDET-ND---    656
HPyV7       EIQEEVVNWKQTFEQWVSYGMFQTMKENILSGKDPFEGVLINDPTE---ENTRE-TQ---    659
QPyV        EIQEDVVNWKQTFERWVTYGMYQDMKQNILAGKDPFYGVIMSDIN----EIVEE-TQ---    654
SV40        SIQSRIVEWKERLDKEFSLSVYQKMKFNVAMGIGVLDWLRNSDDDDEDSQENADKNED--    648
BKV         DIQSRIVEWKERLDSEISMYTFSRMKYNICMGKCILDITREEDSETEDSG----------    642
JCV         AIHERIVQWKERLDLEISMYTFSTMKANVGMGRPILDFPREEDSEAEDSG----------    641
KIPyV       EIQSNVVYWKEVLDNYIGLTEFATMQMNVTNGKNILEKWFE-------------------    641
WUPyV       CIQAKVVYWKELLDKYIGLTEFADMQMNVTNGCNILEKHNA-------------------    648
HPyV10      EIHDNVRYWKETIEKYVPFGMYHDIRRNIESGEDPLKDILICVDADEDT------QQ---    661
STLPyV      SIHEDVKLWKDTLTKYVSIGMFHDIQKNIQNGEDPLKNILICEDTENNE------TQ---    647
TSPyV       SIKEDVKYWKDILCKYMGHTNFATLLLNVEEGKDPLDSVVIEVEDEEEEEFSE--TN---    690
HPyV9       KVQEYVVKWKEILERHVSITQFGNIQQNILDGKDPLHGIVIEEQA---------------    680
HPyV12      SLRETIEIEKKLLESICTTEIACLMKDNIKAGRDPLHDIVTEADE---------------    708
LiPyV       EIRPEVERWRDAFRGDIPQAHFEKMIQNVECGLDPLEDLFVEAPADAPPAAAPEDSEAEP    756
MCPyV       CLQEEIKNWKQILQSEISYGKFCQMIENVEAGQDPLLNILIEEEGPEETE----------    807
NJPyV       SLQEEIANWKCIIEKTVSHSDFCKMLENIEVGESPLTDLIDEGDN---------------    711
MPyV        DIQGLVKEVRDQFASECSYSLFCDILCNVQEGDDPLKDICDIAEYTVY------------    785
HaPyV       SLKEKVETEKKVLSDMCNFTTFAEMCLNIQRGADPLEAL---------------------    751
             ::  :   :  :            :  *:  *   :

HPyV6       -----------------------------STQESGIGSMHSM------------------    669
HPyV7       -----------------------------ESTESGIGSMNN-------------------    671
QPyV        -----------------------------ESTESGVGSMET-------------------    666
SV40        -------------------------GGEKNMEDSGHETGIDSQSQGSF------QAPQSS    677
BKV         -----------------------------------HGSSTESQSQCSSQVSDTSAPAEDS    667
JCV         -----------------------------------HGSSTESQSQCFSQVSEASGAD---    663
KIPyV       ------------------------------------------------------------    641
WUPyV       ------------------------------------------------------------    648
HPyV10      --------------------------------DSG----INSQ-----------------    668
STLPyV      --------------------------------DSA----FCTQDSDNE------------    659
TSPyV       --------------------------------DSG----FQTQ-----------------    697
HPyV9       ------------------------------------------------------------    680
```

```
HPyV12      ------------------------------------------------------------    708
LiPyV       PVASKVPHQHENTMEEPERQQKARPEKDLESQDSG----LFTQDSGQT------------    800
MCPyV       -----------------------------ETQDSG----TFSQ-----------------    817
NJPyV       ------------------------------------------------------------    711
MPyV        ------------------------------------------------------------    785
HaPyV       ------------------------------------------------------------    751

HPyV6       -------------------------------        669
HPyV7       -------------------------------        671
QPyV        -------------------------------        666
SV40        QSVHDHNQPYHICRGFTCFKKPPTPPPEPET        708
BKV         QRSDPHSQELHLCKGFQCFKRPKTPPPK---        695
JCV         ---TQENCTFHICKGFQCFKKPKTPPPK---        688
KIPyV       -------------------------------        641
WUPyV       -------------------------------        648
HPyV10      -------------------------------        668
STLPyV      -------------------------------        659
TSPyV       -------------------------------        697
HPyV9       -------------------------------        680
HPyV12      -------------------------------        708
LiPyV       -------------------------------        800
MCPyV       -------------------------------        817
NJPyV       -------------------------------        711
MPyV        -------------------------------        785
HaPyV       -------------------------------        751
```

Supplementary Figure S2: Alignment sT

>MPyV sT (J02288): 196 aa
MDRVLSRADKERLLELLKLPRQLWGDFGRMQQAYKQQSLLLHPDKGGSHALMQELNSLWGTFKTEVYNLRMNLGG
TGFQVRRLHADGWNLSTKDTFGDRYYQRFCRMPLTCLVNVKYSSCSCILCLLRKQHRELKDKCDARCLVLGECFC
LECYMQWFGTPTRDVLNLYADFIASMPIDWLDLDVHSVYNPTGLSP

>sT HaPyV (NC_001663): 194 aa
MDRILTKEEKQALISLLDLEPQYWGDYGRMQKCYKKKCLQLHPDKGGNEELMQQLNTLWTKLKDGLYRVRLLLGP
SQVRRLGKDQWNLSLQQTFSGTYFRRLCRLPITCLRNKGISTCNCILCLLRKQHFLLKKSWRVPCLVLGECYCID
CFALWFGLPVTNMLVPLYAQFLAPIPVDWLDLNVHEVYNPASGP

>SV40 st-ag (NC_001669): 174 aa
MDKVLNREESLQLMDLLGLERSAWGNIPLMRKAYLKKCKEFHPDKGGDEEKMKKMNTLYKKMEDGVKYAHQPDFG
GFWDATEVFASSLNPGVDAMYCKQWPECAKKMSANCICLLCLLRMKHENRKLYRKDPLVWVDCYCFDCFRMWFGL
DLCEGTLLLWCDIIGQTTYRDLKL

>BKPyV st-ag (NC_001538): 172 aa
MDKVLNREESMELMDLLGLERAAWGNLPLMRKAYLRKCKEFHPDKGGDEDKMKRMNTLYKKMEQDVKVAHQPDFG
TWSSSEVCADFPLCPDTLYCKEWPICSKKPSVHCPCMLCQLRLRHLNRKFLRKEPLVWIDCYCIDCFTQWFGLDL
TEETLQWWVQIIGETPFRDLKL

>JCPyV st-ag (NC_001699): 172 aa
MDKVLNREESMELMDLLGLDRSAWGNIPVMRKAYLKKCKELHPDKGGDEDKMKRMNFLYKKMEQGVKVAHQPDFG
TWNSSEVGCDFPPNSDTLYCKEWPNCATNPSVHCPCLMCMLKLRHRNRKFLRSSPLVWIDCYCFDCFRQWFGCDL
TQEALHCWEKVLGDTPYRDLKL

>KIPyV (NC_009238): 191 aa
MDKTLSREEAKQLMQLLCLDMSCWGNLPLMRRQYLVKCKEYHPDKGGNEESMKLLNSLYLKLQDSVSSVHDLNEE
EDNIWQSSQVYCKDLCCNKFRLVGAIYGDYYEAYIMKQWDVCIHGYNHECQCIHCILSKYHKEKYKIYRKPPVWI
ECYCYKCYREWFFFPISMQTFFFWKVIIFNTEIRAVQPLLR

>WUPyV (NC_009539): 194 aa
MDKTLSRNEAKELMQLLGLDMTCWGNLPLMRTKYLSKCKEFHPDKGGNEEKMKKLNSLYLKLQECVSTVHQLNEE
EDEVWSSSQVECTELCCNFPPRKYRLVGEVYGDVFEEYILKDWDICLKGFYYLCNCFYCFLDKRHKQKYKIFRKP
PMWIECYCYRCYREWFGFEISAETFFYWKKIIFLTTMQGVGLTR

>MCPyV (NC_010277): 196 aa
MDLVLNRKEREALCKLLEIAPNCYGNIPLMKAAFKRSCLKHHPDKGGNPVIMMELNTLWSKFQQNIHKLRSDFSM
FDEVSTKFPWEEYGTLKDYMQSGYNARFCRGPGCMLKQLRDSKCACISCKLSRQHCSLKTLKQKNCLTWGECFCY
QCFILWFGFPPTWESFDWWQKTLEETDYCLLHLHLF

>HPyV6 st-ag (NC_014406): 190 aa
MDRLLAREEVRELMDLIGLSMACWGNLPLMQQKIRLACKKYHPDKGGDPEKMQRLNVLKEKLNATLRDQMSSSPT
WCFSSEVSDDWGIPLTVGEFLGPEFHKKKVWDFRLCVQQGISSCKCLHCLLKKEHKKQVEINLGKPTIWGKCWCY
KCYCLWFGLPVEADSFMWWTHIIYQSPLDWLGITEKLIWW

>HPyV7 st-ag (NC_014407): 193 aa
MDKLLGRDEVKELMELIGLNMACWGNLPLIQHKVRLASKKYHPDKGGDPQKMQRLNVLKDKLQATLRDQRSGSPM
WHYSSDEVSFWDIELTVGEFLGPEFNRKKVWNYNLCVVQGLRACCCIHCILKRKHKKKAKEYAKDHRGPLLWGKC
WCFDCYLDWFGVERSEESFMWWSHIIFQTPMDVLNLWGQLNLL

>TSPyV st-ag (NC_014361): 198 aa
MDKFLSREESLELMDLLQIPRHCYGNFALMKINHKKMSLKYHPDKGGDPEKMSRLNQLWQKLQEGIYNARQEFPT
SFSSQVGSWYWEANLISLKEYFGKKKYDENVIKHWPQCAEKALKECKCLTCKIGLQHHVYKQMHQKKCVVWGECF
CYKCYCAWFGEDLYCLDSLWAWSCIVGEVDFHLVNLYLRVNQGFNWGK

>HPyV9 (NC_015150): 189 aa
MDQTLSLEERNELMDLLQLTRAAWGNLSLMKKAYKTVSKIYHPDKGGNPEKMQRLNELFQKLQVTLLEIRSNCGS
SSSQVAWYFWDENFRTLGAFLGEKFNQRIIGGYPDCITYNKPSCCCIVCLLKQQHKSTKINKKKPCLVWGECFCY
KCYLLWFGFPEDFTSFNYWTLLMRNMDLSLLRLWTELGF

>HPyV10 (JX262162): 206 aa
MDRVLSRDEVKELMALLSLNTAAWGNIPLMQYKYRQTCLKLHPDKGGDGEKMKRLNELFSKMYTTIEKLRREGEV
YFPAKVGYFIDDVVTLGDVLGPSFEEKIIYIWPLCASDLLRHKCGCVCCLLKKQHRNDKLAKQKQCLVWGECFCY
KCFLLWFGQEFGYTSFFWWKHIMHNTEFDLLCLLGELILWVSYFSFILGKSHLWDS

>STLPyV (NC_020106): 195 aa
MDQALSRQEAKELMGLLGLPEDSWGNVPLITYRFRQKSKIYHPDKGGNEETMKRMTELYSRMQNTLQNLRSSNEN
ENMYPPVRMLLLTDTFTLGELLGPQFESKVIFIWPTCAKCRYRTFCQCVCCILKRQHDEIKKVRNKPCVTWGECY
CFDCFLLWFGCDLTKASLHAWKHVMYNLDLDLLMFKQLNLVSFSF

>HPyV12 (NC_020890): 182 aa
MDSILTFAERQLLISLLKISGDTFGNVPAMARAYKLAAKRLHPDKGGNEAEMKKLNELWNKFKDGIYNLREVKPS
LHPVVTCTVLGARNIFNLITNSSQCMRNLLRYCRCFCCILFQQHRQLKITYRRRCNVWGQCYCFLCYYTWFGVNC
SIGAFTEWLILLKHLDWRLLKISSAELDVLGK

>NJPyV (NC_024118): 183 aa
MEKVLEKSDKEMLIELLGIPRYAYGNFPIMKTAYKRASKIYHPDKGGSSEKMMLLNSLWQKFQEGLIEVRDSEVC
QVSFSDCYDSSLLKCCSPKVFHELFLRSPQCLLKGPTSCSCITSCLYNQHRQIKLCGKKRCLTWGNCFCFSCFIL
WFGLRETWKTFEIWKHVIAQMPAALLQLSPSLF

>LiPyV (NC_034253: 179 aa
MDAVLTTPERRQLCLLLDISPQEYGNIPLMKNAFKKACLKHHPDKGGDPVLMMQLNSLWGKFTTSLTEARASTYQ
ASTLFWEIDNPLKNLLGPVIKRPFLKSPHCINSKFYNCRCIVCSLSDQHSSLKILQKKKCLIWGECYCYYCFVTW
FGLPGNSATFEDYKNLILEMDVDLLNLHC

>QPyV (BK010702): 193 aa
MDRLLSRDEVNELMQLIGLSMSNWGNLPLIQHKVREACKKHHPDKGGDPEKMQRLNVLKDKFAATMRDQSSGNPI
WHFSSEEVSFWDLQLTVGEFLGVEFNRKKLWNFELCVLQGLRACCCLHCLLRRKHKKLAKQMAKDQKGPLVWGHC
WCFQCYLQWFGEDKNKESFEWWTQIIYGTQMDVINIWGQINLL

CLUSTAL O(1.2.4) multiple sequence alignment

```
SV40        MDKVLNREESLQLMDLLGLERSAWGNIPLMRKAYLKKCKEFHPDKGGDEEKMKKMNTLYK    60
BKPYV       MDKVLNREESMELMDLLGLERAAWGNLPLMRKAYLRKCKEFHPDKGGDEDKMKRMNTLYK    60
JCPYV       MDKVLNREESMELMDLLGLDRSAWGNIPVMRKAYLKKCKELHPDKGGDEDKMKRMNFLYK    60
KIPyV       MDKTLSREEAKQLMQLLCLDMSCWGNLPLMRRQYLVKCKEYHPDKGGNEESMKLLNSLYL    60
WUPyV       MDKTLSRNEAKELMQLLGLDMTCWGNLPLMRTKYLSKCKEFHPDKGGNEEKMKKLNSLYL    60
HPyV12      MDSILTFAERQLLISLLKISGDTFGNVPAMARAYKLAAKRLHPDKGGNEAEMKKLNELWN    60
MPyV        MDRVLSRADKERLLELLKLPRQLWGDFGRMQQAYKQQSLLLHPDKGGSHALMQELNSLWG    60
sT          MDRILTKEEKQALISLLDLEPQYWGDYGRMQKCYKKKCLQLHPDKGGNEELMQQLNTLWT    60
NJPyV       MEKVLEKSDKEMLIELLGIPRYAYGNFPIMKTAYKRASKIYHPDKGGSSEKMMLLNSLWQ    60
MCPyV       MDLVLNRKEREALCKLLEIAPNCYGNIPLMKAAFKRSCLKHHPDKGGNPVIMMELNTLWS    60
LiPyV       MDAVLTTPERRQLCLLLDISPQEYGNIPLMKNAFKKACLKHHPDKGGDPVLMMQLNSLWG    60
TSPyV       MDKFLSREESLELMDLLQIPRHCYGNFALMKINHKKMSLKYHPDKGGDPEKMSRLNQLWQ    60
HPyV6       MDRLLAREEVRELMDLIGLSMACWGNLPLMQQKIRLACKKYHPDKGGDPEKMQRLNVLKE    60
HPyV7       MDKLLGRDEVKELMELIGLNMACWGNLPLIQHKVRLASKKYHPDKGGDPQKMQRLNVLKD    60
QPyV        MDRLLSRDEVNELMQLIGLSMSNWGNLPLIQHKVREACKKHHPDKGGDPEKMQRLNVLKD    60
HPyV9       MDQTLSLEERNELMDLLQLTRAAWGNLSLMKKAYKTVSKIYHPDKGGNPEKMQRLNELFQ    60
HPyV10      MDRVLSRDEVKELMALLSLNTAAWGNIPLMQYKYRQTCLKLHPDKGGDGEKMKRLNELFS    60
STLPyV      MDQALSRQEAKELMGLLGLPEDSWGNVPLITYRFRQKSKIYHPDKGGNEETMKRMTELYS    60
            *:  *   :   *  *: :    :*:   :       .   ******.   *  :. *

SV40        KMEDGVKYAHQPD--FGGFWDATEVFASS----LNPGV-------------DAMYCKQWP    101
BKPYV       KMEQDVKVAHQPD--FGT-WSSSEV-CAD----FPLCP-------------DTLYCKEWP    99
JCPYV       KMEQGVKVAHQPD--FGT-WNSSEV-GCD----FPPNS-------------DTLYCKEWP    99
KIPyV       KLQDSVSSVHDLNEEEDNIWQSSQVYCKDLCCN----KFRLVGAIYGD-YYEAYIMKQWD    115
WUPyV       KLQECVSTVHQLNEEEDEVWSSSQVECTELCCNFPPRKYRLVGEVYGD-VFEEYILKDWD    119
HPyV12      KFKDGIYNLREVKP----SLHP-----------------VVTCTVLGA-RNIFNLITNSS    98
MPyV        TFKTEVYNLRMNLG----GTGF-QVRRL-----HADGWNLSTKDTFGD-RYYQRFCRMPL    109
sT          KLKDGLYRVRLLLG----PS---QVRRL-----GKDQWNLSLQQTFSG-TYFRRLCRLPI    107
NJPyV       KFQEGLIEVRDSEV----CQV-SFSDC--------Y-DSSLLKCCSPK-VFHELFLR-SP    104
MCPyV       KFQQNIHKLRSDFS----MFD-EVSTKF-----PWE-EYGTLKDYMQS-GYNARFCR-GP    107
LiPyV       KFTTSLTEARASTY---------QASTL-----FWE-IDNPLKNLLGP-VIKRPFLK-SP    103
```

```
TSPyV       KLQEGIYNARQEFP----TSFSSQVGSW-----YWEANLISLKEYFGKKKYDENVIKHWP    111
HPyV6       KLNATLRDQMSSSP----TWCFSSEVSD-----DWG-IPLTVGEFLGP-EFHKKKVWDFR    109
HPyV7       KLQATLRDQRSGSP----MWHYSSDEVS-----FWD-IELTVGEFLGP-EFNRKKVWNYN    109
QPyV        KFAATMRDQSSGNP----IWHFSSEEVS-----FWD-LQLTVGEFLGV-EFNRKKLWNFE    109
HPyV9       KLQVTLLEIRSNCG----SSS-SQVAWY-----FWDENFRTLGAFLGE-KFNQRIIGGYP    109
HPyV10      KMYTTIEKLRRE------GEVYFPAKVG-----YFIDDVVTLGDVLGP-SFEEKIIYIWP    108
STLPyV      RMQNTLQNLRSSNE----NENMYPPVRM-----LLLTDTFTLGELLGP-QFESKVIFIWP    110
             :   :

SV40        ECAKKMS--ANCICLLCLLRMKHENRKL----YRKDPLVWVDCYCFDCFRMWFGLDLC-E    154
BKPYV       ICSKKPS--VHCPCMLCQLRLRHLNRKF----LRKEPLVWIDCYCIDCFTQWFGLDLT-E    152
JCPYV       NCATNPS--VHCPCLMCMLKLRHRNRKF----LRSSPLVWIDCYCFDCFRQWFGCDLT-Q    152
KIPyV       VCIHGYN--HECQCIHCILSKYHKEKYK----IYRKPPVWIECYCYKCYREWFFFPIS-M    168
WUPyV       ICLKGFY--YLCNCFYCFLDKRHKQKYK----IFRKPPMWIECYCYRCYREWFGFEIS-A    172
HPyV12      QCMRNLL--RYCRCFCCILFQQHRQLKI---TYRRRCNVWGQCYCFLCYYTWFGVNCS-I    152
MPyV        TCLVNV-KYSSCSCILCLLRKQHRELKD---KCDARCLVLGECFCLECYMQWFGTPTR-D    164
sT          TCLRNK-GISTCNCILCLLRKQHFLLKK---SWRVPCLVLGECYCIDCFALWFGLPVT-N    162
NJPyV       QCLLKGP--TSCSCITSCLYNQHRQIKL---CGKKRCLTWGNCFCFSCFILWFGLRET-W    158
MCPyV       GCMLKQLRDSKCACISCKLSRQHCSLKT---LKQKNCLTWGECFCYQCFILWFGFPPT-W    163
LiPyV       HCINSKF--YNCRCIVCSLSDQHSSLKI---LQKKKCLIWGECYCYYCFVTWFGLPGN-S    157
TSPyV       QCAEKAL--KECKCLTCKIGLQHHVYKQ---MHQKKCVVWGECFCYKCYCAWFGEDLYCL    166
HPyV6       LCVQQGI--SSCKCLHCLLKKEHKKQV---EINLGKPTIWGKCWCYKCYCLWFGLPVE-A    163
HPyV7       LCVVQGL--RACCCIHCILKRKHKKKAKEYAKDHRGPLLWGKCWCFDCYLDWFGVERS-E    166
QPyV        LCVLQGL--RACCCLHCLLRRKHKKLAKQMAKDQKGPLVWGHCWCFQCYLQWFGEDKN-K    166
HPyV9       DCITYN-K-PSCCCIVCLLKQQHKSTKI---NKKKPCLVWGECFCYKCYLLWFGFPED-F    163
HPyV10      LCASDLLR-HKCGCVCCLLKKQHRNDKL---AKQKQCLVWGECFCYKCFLLWFGQEFG-Y    163
STLPyV      TCAKCRYR-TFCQCVCCILKRQHDEIKK---VRNKPCVTWGECYCFDCFLLWFGCDLT-K    165
             *         * *. . :   *                  .*:*  *:  **

SV40        GTLLLWCDIIGQTTYRDLKL-----------------------    174
BKPYV       ETLQWWVQIIGETPFRDLKL-----------------------    172
JCPYV       EALHCWEKVLGDTPYRDLKL-----------------------    172
KIPyV       QTFFFWKVIIFNTEIRAVQPLLR--------------------    191
WUPyV       ETFFYWKKIIFLTTMQGVGLTR---------------------    194
HPyV12      GAFTEWLILLKHLDWRLLKISSAELDV-------LGK------    182
MPyV        V-LNLYADFIASMPIDWLDLDVHSVYNPTGLSP----------    196
sT          MLVPLYAQFLAPIPVDWLDLNVHEVYNPASGP-----------    194
NJPyV       KTFEIWKHVIAQMPAALLQLSPSLF------------------    183
MCPyV       ESFDWWQKTLEETDYCLLHLHLF--------------------    186
LiPyV       ATFEDYKNLILEMDVDLLNLHC---------------------    179
TSPyV       DSLWAWSCIVGEVDFHLVNLYLRVNQGFNWGK-----------    198
HPyV6       DSFMWWTHIIYQSPLDWLGITEKLIWW----------------    190
HPyV7       ESFMWWSHIIFQTPMDVLNLWGQLNLL----------------    193
QPyV        ESFEWWTQIIYGTQMDVINIWGQINLL----------------    193
HPyV9       TSFNYWTLLMRNMDLSLLRLWTELGF-----------------    189
HPyV10      TSFFWWKHIMHNTEFDLLCLLGELILWVSYFSFILGKSHLWDS    206
STLPyV      ASLHAWKHVMYNLDLDLLMFKQLNLVSFSF-------------    195
              .  :   :       :
```

Supplementary Figure S3: Alignment MT

```
>MPyV: 431 aa
MDRVLSRADKERLLELLKLPRQLWGDFGRMQQAYKQQSLLLHPDKGGSHALMQELNSLWGTFKTEVYNLRMNLGG
TGFQVRRLHADGWNLSTKDTFGDRYYQRFCRMPLTCLVNVKYSSCSCILCLLRKQHRELKDKCDARCLVLGECFC
LECYMQWFGTPTRDVLNLYADFIASMPIDWLDLDVHSVYNPKRRSEELRRAATVHYTMTTGHSAMEASTSQGNGM
ISSESGTPATSRRLRLPSLLSNPTYSVMRSHSYPPTRVLQQIHPHILLEEDEILVLLSPMTAYPRTPPELLYPES
DQDQLEPLEEEEEEYMPMEDLYLDILPGEQVPQLIPPPIIPRAGLSPWEGLILRDLQRAHFDPILDASQRMRATH
RAALRAHSMQRHLRRLGRTLLLVTFLAALLGICLMLFILIKRSRHF

>STLPyV: 229 aa
MDQALSRQEAKELMGLLGLPEDSWGNVPLITYRFRQKSKIYHPDKGGNEETMKRMTELYSRMQNTLQNLRSSNEN
ENMYPPVRMLLLTDTFTLGELLGPQFESKVIFIWPTCAKCRYRTFCQCVCCILKRQHDEIKKVRNKPCVTWGECY
CFDCFLLWFGCDLTKASLHAWKHVMYNLDLDLLMFKQLNLVMMKGPLHPKKENFLILLLRIQHHLRKISQQIQQI
FLLN

>TSPyV: 332 aa
MDKFLSREESLELMDLLQIPRHCYGNFALMKINHKKMSLKYHPDKGGDPEKMSRLNQLWQKLQEGIYNARQEFPT
SFSSQVGSWYWEANLISLKEYFGKKKYDENVIKHWPQCAEKALKECKCLTCKIGLQHYVYKQMHQKKCVVWGECF
CYKCYCAWFGEDLYCLDSLWAWSCIVGEVDFHLVNLYLRVNQGFNWVFPFSMMFQPRMEEIYLPMGTPPGPAGGK
ASIKNGTTCLTPCRTQTSSAMNPPFPLMNLDLQAPLRDPLLNLARRIQEEEELPRQRTPPAAPRAPSLPPPQSQK
NLSMTLSLMIFLICCGLFFLMLSIVIKLYHLF
```

```
CLUSTAL O(1.2.4) multiple sequence alignment


MPyV            MDRVLSRADKERLLELLKLPRQLWGDFGRMQQAYKQQSLLLHPDKGGSHALMQELNSLWG      60
STLPyV          MDQALSRQEAKELMGLLGLPEDSWGNVPLITYRFRQKSKIYHPDKGGNEETMKRMTELYS      60
TSPyV           MDKFLSREESLELMDLLQIPRHCYGNFALMKINHKKMSLKYHPDKGGDPEKMSRLNQLWQ      60
                **: *** :  .*: ** :*.. :*:.  :   .:: *   ******.   *..:..*:

MPyV            TFKTEVYNLRMNLGGTG-FQVRRLHADGWNLSTKDTFGDRYYQR-FCRMPLTCLVNVKYS      118
STLPyV          RMQNTLQNLRSSNENENMYPPVRMLLLTDTFTLGELLG-PQFESKVIFIWPTCAKCRYRT      119
TSPyV           KLQEGIYNARQEFPTSFSSQVGSWYWEANLISLKEYFGKKKYDENVIKHWPQCAEKAL-K      119
                 ::  : * * .                  ::  : :*   ::  .      *      .

MPyV            SCSCILCLLRKQHRELKDKCDARCLVLGECFCLECYMQWFGTPTRDVLNLYAD--FIASM      176
STLPyV          FCQCVCCILKRQHDEIKKVRNKPCVTWGECYCFDCFLLWFGCDLTK-ASLHAWKHVMYNL      178
TSPyV           ECKCLTCKIGLQHYVYKQMHQKKCVVWGECFCYKCYCAWFGEDLYCLDSLWAWSCIVGEV      179
                 *.*: * :  **   *.  :  *:. ***:* .*:  ***       .* *   .: .:

MPyV            PIDWLDLDVHSVYNPKRRSEELR--RAATVHYTMTTGHSAMEASTSQGNGMISSESGTPA      234
STLPyV          D-----LDLLMFK-----QLNLVMMK-GPLHPKKENFL----------------------      205
TSPyV           D-----FHLVNLYLRVNQGFNWVFPFSMMFQPRMEEIYLPMGTPPGPAGGKASIKNGTTC      234
                      :.:  .        :        .:

MPyV            TSRRLRLPSLLSNPTYSVMRSHSYPPTRVLQQIHPHILLEEDEILVLLSPMTAYPRTPPE      294
STLPyV          -----------------ILLLRIQHHLRK-------------------------------      217
TSPyV           LTPCRTQTSSAMNPPFPLMNLDLQAPLRD--------------------PL---------      265
                                 ::        *

MPyV            LLYPESDQDQLEPLEEEEEEYMPMEDLYLDILPGEQVPQLIPPPIIPRAGLSPWEGLILR      354
STLPyV          --------------------------ISQQIQQIFLLN----------------------      229
TSPyV           ------------------------LNLARRIQEEEELPRQRTPPAAPRAPSLPPP-----      296
                                          :   *     :

MPyV            DLQRAHFDPILDASQRMRATHRAALRAHSMQRHLRRLGRTLLLVTFLAALLGICLMLFIL      414
STLPyV          ------------------------------------------------------------      229
TSPyV           -------------------------------QSQKNLSMTLSLMIFLICCGLFFLMLSIV      325


MPyV            IKRSRHF      421
STLPyV          -------      229
TSPyV           IKLYHLF      332
```
